# Isobenzofuranones and Isochromenones from the Deep-Sea Derived Fungus *Leptosphaeria* sp. SCSIO 41005

**DOI:** 10.3390/md15070204

**Published:** 2017-06-29

**Authors:** Xiaowei Luo, Xiuping Lin, Limbadri Salendra, Xiaoyan Pang, Yu Dai, Bin Yang, Juan Liu, Junfeng Wang, Xuefeng Zhou, Yonghong Liu

**Affiliations:** 1 Chinese Academy of Sciences (CAS) Key Laboratory of Tropical Marine Bio-resources and Ecology/Guangdong Key Laboratory of Marine Materia Medica, South China Sea Institute of Oceanology, CAS, Guangzhou 510301, China; luoxiaowei14@mails.ucas.ac.cn (X.L.); xiupinglin@hotmail.com (X.L.); raj.badri202@gmail.com (L.S.); luckygirlpxy@163.com (X.P.); daiyu15@mails.ucas.ac.cn (Y.D.); bingo525@163.com (B.Y.); liujuan@scsio.ac.cn (J.L.); junfeng1982a@163.com (J.W.); 2 University of Chinese Academy of Sciences, Beijing 100049, China

**Keywords:** leptosphaerins, clearanols, deep-sea fungi, *Leptosphaeria* sp.

## Abstract

Four new isobenzofuranones, leptosphaerins J–M (**1**–**4**), including an unusual naturally-occurring centrosymmetric dimer skeleton (**1**), and two new isochromenones, clearanols I–J (**9**–**10**), were obtained from a culture of a deep-sea sediment-derived fungus *Leptosphaeria* sp. SCSIO 41005, together with four known isobenzofuranones (**5**–**8**) and six known isochromenones (**11**–**16**). These structures were elucidated by extensive spectroscopic analyses, and absolute configurations were assigned on the basis of electronic circular dichroism and optical rotations data comparison. Additionally, the absolute configurations of the new compounds **1** and **9**, together with the known one **7** with stereochemistry undetermined, were further confirmed by single crystal X-ray diffraction experiments. A plausible biosynthetic pathway of these isobenzofuranones and isochromenones was also proposed.

## 1. Introduction

Deep-sea derived microorganisms are a promising source of structurally diverse and versatile bioactive secondary metabolites, owing to the extreme conditions in the deep sea, such as the absence of light, low levels of oxygen and intensely high pressures, where a plethora of organisms abound with various biochemical and physiological adaptations to unique environments [1,2]. The species of the ascomycete fungus *Leptosphaeria* are a rich origin of new bioactive compounds, such as polyketides with cytotoxic [3,4,5] and antifungal activities [6,7], dioxopiperazine alkaloids with cytotoxic [8,9,10] and phytotoxic activities [11,12], and also phytotoxic sesquiterpenoids [13] and pentacyclic triterpenes [14].

**Figure 1:**
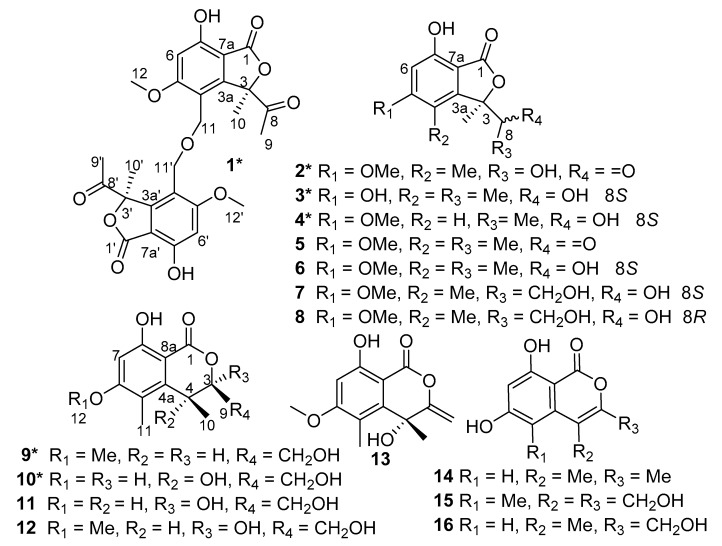
Chemical structures of compounds **1**–**16** (* new compounds).

During our continuous study in exploring new bioactive natural products from marine microorganisms [15,16,17,18], the deep-sea-derived fungus *Leptosphaeria* sp. SCSIO 41005, isolated from a marine sediment sample collected from the Indian Ocean at a depth of 3614 m, was investigated. The chemical study on the ethyl acetate extract of this fungus grown on rice medium led to the isolation of 16 compounds, including 8 isobenzofuranones, leptosphaerins J–M (**1**–**4**), (*R*)-3-acetyl-7-hydroxy-5-methoxy-3,4-dimethylisobenzofuran-1(3*H*)-one (**5**) [6], (3*R*,3^1^*S*)-7-hydroxy-3-(1-hydroxyethyl)-5-methoxy-3,4-dimethylisobenzofuran-1(3*H*)-one (**6**) [6], clearanol E (**7**) [19], clearanol D (**8**) [19] and 8 isochromenones, clearanols I–J (**9**–**10**), dothideomynone A (**11**) [20], 3,8-dihydroxy-3-hydroxymethyl-6-methoxy-4,5-dimethylisochromen-1-one (**12**) [21], (*R*)-4,8-dihydroxy-6-methoxy-4,5-dimethyl-3-methyleneisochromen-1-one (**13**) [6], 6,8-dihydroxy-3,4-dimethylisocoumarin (**14**) [22], acremonone F (**15**) [23] and acremonone G (**16**) [23] (Figure 1). The isolation and structural elucidation of all the compounds are described herein, including assignment of their absolute configurations.

## 2. Results and Discussion

The culture broth of fungus *Leptosphaeria* sp. SCSIO 41005 was extracted with ethyl acetate at room temperature. The solvent was evaporated in vacuo to afford a crude extract, which was subjected to various chromatographic methods to yield compounds **1**–**16**.

**Figure 2:**
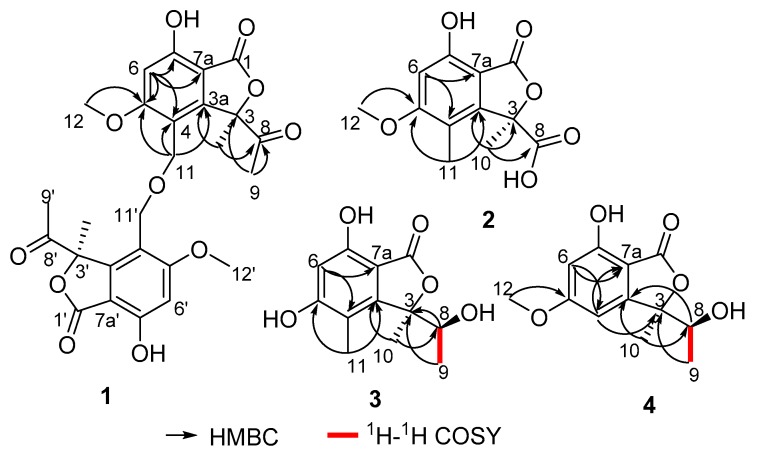
Selected HMBC and COSY correlations of compounds **1**–**4**.

**Figure 3:**
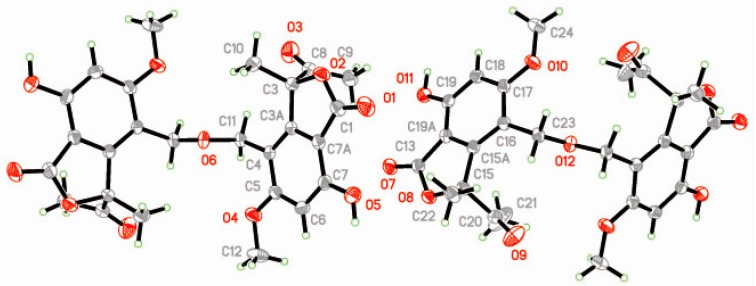
X-ray crystallographic structure of compound **1** (Gray: carbon atom; green: hydrogen atom; red: oxygen atom).

Leptosphaerin J (**1**) was isolated as colorless crystals. A molecular formula of C_26_H_26_O_11_ was derived from a sodium adduct ion peak at *m*/*z* 537.1361 [M + Na]^+^ (calcd. for 537.1373) in the (+)-HRESIMS. The ^1^H NMR spectrum (Table 1) of **1** exhibited two primary methyls (*δ*_H_ 1.69, 1.94), one oxygen-bearing methylene (*δ*_H_ 4.25, 4.43), one methoxy (*δ*_H_ 3.90), and one aromatic proton (*δ*_H_ 6.59). The analysis of the ^13^C NMR and DEPT spectra (Table 1) revealed thirteen carbons, ascribed to three methyls (one oxygenated), one oxygenated methylene, one aromatic methine and eight non-protonated carbons (one oxygenated, one carbonyl, one *α*, *β*-conjugated carbonyl and five aromatics). The HMBC spectrum of **1** showed the correlations from H-6 to C-4, C-5, C-7 and C-7a, from H_3_-9 to C-3 and C-8, from H_3_-10 to C-3, C-3a and C-8, in turn coupled with H_3_-9 (Figure 2). All the above data suggested that **1** presented an isobenzofuranone skeleton, which is similar to (*R*)-3-acetyl-7-hydroxy-5-methoxy-3,4-dimethylisobenzofuran-1(3H)-one (**5**) [6], the known compound we also obtained, except the primary methyl in **5** was replaced with an oxygenated methylene in **1**. Due to the molecular formula of C_26_H_26_O_11_ given by HRESIMS, compound **1** was supposed to be a centrosymmetric dimer skeleton of isobenzofuranone. The dimer skeleton, conjugated by an ether bond on CH_2_-11 as shown in Figure 1, was confirmed by X-ray diffraction analysis, using the anomalous scattering of Cu K*α* radiation yielding a Flack parameter of 0.00 (5) (Figure 3).

**Figure 4:**
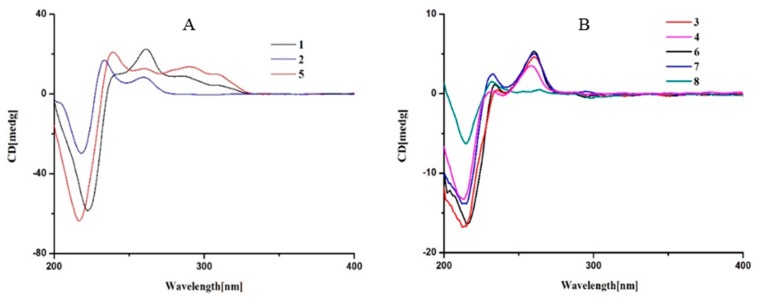
Experimental ECD spectra of compounds **1**–**8** ((**A**) **1**, **2** and **5**; (**B**) **3**, **4** and **6**–**8**).

Compounds **1** and **5** showed similar optical rotation (${\left[\alpha \right]}_{D}^{25}$ = +87.3° (*c* 0.06, MeOH) for **1**, and ${\left[\alpha \right]}_{D}^{25}$ = +108.4° (*c* 0.15, MeOH) for **5**) and similar Cotton effects in electronic circular dichroism (ECD) spectrum (Figure 4), indicating **1** and **5** possessed the same absolute configuration of *R*, which was later confirmed by the X-ray diffraction experiment (Figure 3).

Leptosphaerin K (**2**) was obtained as a colorless oil. The molecular formula was deduced to be C_12_H_12_O_6_ by the HRESIMS peak at *m*/*z* 251.0565 [M − H]^−^ (calcd. for C_12_H_11_O_6_, 251.0556). The ^1^H and ^13^C NMR signals were assigned by combination of analyses of DEPT, HSQC and HMBC spectra. The ^1^H NMR spectrum (Table 1) of **2** exhibited two primary methyls (*δ*_H_ 1.78, 2.16), one methoxy (*δ*_H_ 3.82) and one aromatic proton (*δ*_H_ 6.38). The ^13^C NMR and DEPT spectra (Table 1) revealed twelve carbons, including three methyls (one oxygenated), one aromatic methine and eight non-protonated carbons (one oxygenated, two carbonyls and five aromatics). This evidence, including UV and IR spectra (Supplementary Materials Figures S16 and S17), revealed that there was also an isobenzofuranone unit in compound **2**. Furthermore, the HMBC spectrum of **2** also showed the correlations from H-6 to C-4 and C-7a, from H_3_-10 to C-3a and C-8, from H_3_-11 to C-3a, C-4 and C-5, which implied that the isobenzofuranone structure of **2** was identical to that of **1** and **5**. The main difference was the replacement of a methyl group (C-9) in **5** by a hydroxyl group in **2**. Besides, the similar Cotton effects of **2** and **5** in their ECD spectra (Figure 4) indicated their same absolute configuration at C-3. Thus, it was unambiguous to elucidate the structure of **2** as shown in Figure 1.

Leptosphaerin L (**3**) was obtained as colorless oil. The HRESIMS ion peak at *m*/*z* 237.0775 [M − H]^−^ (calcd. for C_12_H_13_O_5_, 237.0763) and the NMR spectroscopy revealed a molecular formula of C_12_H_14_O_5_. The ^1^H NMR spectrum (Table 1) of **3** exhibited three primary methyls (*δ*_H_ 0.85, 1.71, 2.09), one oxymethine (*δ*_H_ 4.22) and one aromatic proton (*δ*_H_ 6.36). The ^13^C NMR and DEPT spectra (Table 1) revealed twelve carbons, assigned to three methyls, one oxymethine, one aromatic methine and seven non-protonated carbons (one oxygenated, one carbonyl and five aromatics). All these data, as well as UV and IR spectra (Supplementary Materials Figures S24 and S25), suggested that **3** also had an isobenzofuranone moiety. The HMBC spectrum of **3** showed the correlations from H-6 to C-4 and C-7a, from H_3_-9 to C-3 and C-8, from H_3_-10 to C-3a and C-8, and from H_3_-11 to C-3a, C-4 and C-5, which was in accordance with the structure of **6** (Figure 2). The main difference was the replacement of a methoxy group (C-5) in **6** by a hydroxyl group in **3**. Hence, the planar structure of **3** was deduced as shown in Figure 1. Moreover, the similar optical rotation (${\left[\alpha \right]}_{D}^{25}$ = −44.7° (*c* 0.15, MeOH) for **3**, and ${\left[\alpha \right]}_{D}^{25}$ = −35.4° (*c* 0.68, MeOH) for **6**) and almost identical curves in ECD spectrum (Figure 4) indicated compounds **3** and **6** had the same absolute configuration (3*R*, 8*S)* at the position C-3 and C-8.

Leptosphaerin M (**4**) was obtained as a colorless oil. Its HRESIMS ([M − H]^−^, 237.0775; calcd. for C_12_H_13_O_5_^−^, 237.0763) data were in agreement with the molecular formula C_12_H_14_O_5_. The ^1^H NMR spectrum (Table 1) of **4** exhibited two primary methyls (*δ*_H_ 1.05, 1.59), one methoxy (*δ*_H_ 3.84), one oxymethine (*δ*_H_ 3.90) and two aromatic protons (*δ*_H_ 6.39, 6.51). The ^13^C NMR and DEPT spectra (Table 1) revealed the presence of twelve carbon resonances, involving three methyls (one oxygenated), one oxymethine, two aromatic methines and six non-protonated carbons (one oxygenated, one carbonyl and four aromatics). Analyses of the 2D NMR (HSQC and HMBC) data established an isobenzofuranone nucleus. This assignment was evident from the HMBC correlations from H-4 to C-3, C-6 and C-7a, from H-6 to C-4 and C-7a, from H_3_-9 to C-3 and C-8, from H_3_-10 to C-3, C-3a and C-8, and from H_3_-12 to C-5 (Figure 2). The above NMR data of **4** showed that it shared the same isobenzofuranone skeleton as **3**. The main distinctions were the replacement of a hydroxyl group (OH-5) and a methyl group (C-4) in **3** by a methoxy and a proton in **4**, respectively. Consequently, the planar structure of **4** was established as shown in Figure 1. In the same way, the similar optical rotation value (${\left[\alpha \right]}_{D}^{25}$ = −32.6° (*c* 0.10, MeOH) for **4**) and almost identical curves in ECD spectrum (Figure 4) indicated that **4** displayed the same absolute configuration of position C-3 and C-8 (3*R*, 8*S*) with compounds **3** and **6**.

Compounds **7** and **8**, obtained as a pair of structurally-known epimers, were elucidated as clearanol E (**7**) [19] and clearanol D (**8**) [19], by comparison of their NMR and MS data with reported literature. They were reported as a mixture with undetermined stereochemistry. In a comparison between the ECD spectra of **7** and **8**, the main difference was that the positive Cotton effect at 260 nm in **7** was absent in **8** (Figure 4), which was identical to the reported calculated CD spectra of their congeners (*R*,3^1^*S*)-7-hydroxy-3-(1-hydroxyethyl)-5-methoxy-3,4-dimethylisobenzofuran-1(3*H*)-one and (*R*,3^1^*R*)-7-hydroxy-3-(1-hydroxyethyl)-5-methoxy-3,4-dimethylisobenzofuran-1(3*H*)-one, respectively [24]. Thus, the absolute configuration of clearanol E (**7**) and clearanol D (**8**) was determined as 3*R*, 8*S* and 3*R*, 8*R*, respectively, and that of the former was also confirmed on the basis of X-ray crystallographic experiment with a Flack parameter of 0.04(8) by using the anomalous scattering of Cu K*α* radiation (Supplementary Materials Figure S61).

**Figure 5:**
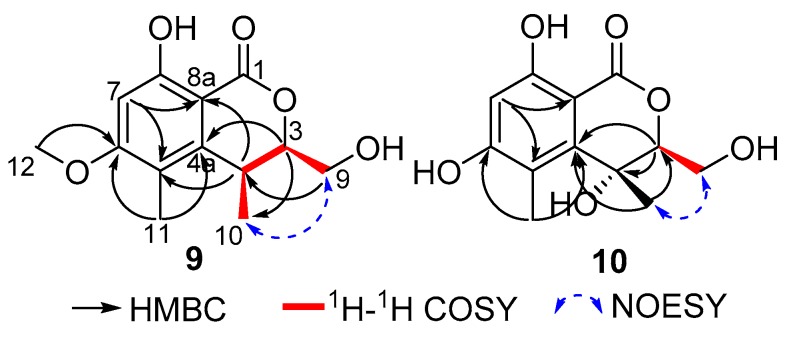
Selected COSY, HMBC, NOSEY correlations of compounds **9** and **10**.

Clearanol I (**9**) was obtained as colorless crystals. It showed a [M + H]^+^ ion peak at *m*/*z* 253.1077 in the positive-ion HRESIMS (calcd. for C_13_H_17_O_5_, 253.1076), appropriate for a molecular formula of C_13_H_16_O_5_. The ^1^H NMR spectrum (Table 2) of **9** exhibited signals for two primary methyl groups (*δ*_H_ 1.07, 2.07), one methoxy group (*δ*_H_ 3.86), one methylene (*δ*_H_ 3.90, 3.80), two methines (*δ*_H_ 3.29, 4.50) and one aromatic proton (*δ*_H_ 6.43). The ^13^C NMR and DEPT spectra (Table 2) exhibited thirteen carbon resonances that consisted of three methyls (one oxygenated), one oxymethylene, three methines (one oxygenated and one aromatic) and six non-protonated carbons (one carbonyl and five olefinics). The above assignment suggested that **9** presented an isochromenone unit, with structural similarities to clearanol F [25], which was further evidenced by COSY (H_2_-9/H-3/H-4/H_3_-10) and HMBC correlations (Figure 5) from H-7 to C-5, C-6, C-8 and C-8a, from H-3 to C-4a, C-9 and C-10, H-4 to C-4a, C-5, C-8a and C-10, from H_2_-9 to C-3 and C-4, from H_3_-10 to C-3, C-4 and C-4a, in turn coupled with H_2_-9. The above NMR data of **9** showed that it shared the same isochromenone skeleton as clearanol F [25]. The main difference was the replacement of a hydroxyl group (OH-4) by a proton in **9**. Therefore, the planar structure of **9**, named as clearanol I, was elucidated as shown in Figure 1.

In the NOESY experiment of **9** (Figure 5), the correlation between H_2_-9 and H_3_-10 suggested that CH_2_-9 and CH_3_-10 are positioned on the same face. Besides, compound **9** (${\left[\alpha \right]}_{D}^{25}$ = +18.2° (*c* 0.19, MeOH)) and clearanol F (${\left[\alpha \right]}_{D}^{25}$ = +18° (*c* 0.05, CHCl_3_)) shared perfectly identical optical rotation value. Furthermore, in analyzing the ECD spectroscopic data, **9** and clearanol F showed almost identical curves (Cotton effect was negative at 224, 250 nm (**9**) and 228, 261 nm (clearanol F), and positive at 237, 270 nm (**9**) and 242, 273 nm (clearanol F), respectively). These data indicated **9** and clearanol F possibly possessed similar stereochemistry, which was further determined as 3*R*, 4*S* on the basis of X-ray crystallographic study with a Flack parameter of −0.03 (5) by using the anomalous scattering of Cu K*α* radiation (Supplementary Materials Figure S62).

Clearanol J (**10**) was obtained as colorless crystals and had the molecular formula C_12_H_14_O_6_, as evidenced by HRESIMS ([M − H]^−^, 253.0721, calcd. for C_12_H_13_O_6_^−^, 253.0712) and the NMR data (Table 2). The ^1^H NMR spectrum (Table 2) of **10** exhibited two primary methyls (*δ*_H_ 1.37, 2.29), one methylene (*δ*_H_ 3.85, 4.06), one methine (*δ*_H_ 4.26) and one aromatic proton (*δ*_H_ 6.28). The analysis of the ^13^C NMR and DEPT spectra (Table 2) revealed twelve carbons, including two methyls, one oxymethylene, two methines (one oxygenated and one aromatic) and seven non-protonated carbons (one oxygenated, one carbonyl and five aromatics). These data suggested that **10** presented an isochromenone moiety, which was similar to that of **9** and clearanol F [25], with the replacement of CH_3_O-6 in clearanol F by a hydroxyl group in **10**, and this was further confirmed by COSY and HMBC correlations (Figure 5). 

In the NOESY spectra of **10** and **9**, there were same correlations between H_2_-9 and H_3_-10 (Figure 5), suggesting that CH_2_-9 and CH_3_-10 are positioned on the same face, both in **10** and **9**. Meanwhile, the perfectly identical optical rotation value (${\left[\alpha \right]}_{D}^{25}$ = +17.4° (*c* 0.10, MeOH)) and similar ECD spectroscopic data (Cotton effect was negative at 230, 258 nm, and positive at 242, 276 nm) of **10** with those of **9** and clearanol F, illustrated **10** possessed the same absolute configuration of 3*R*, 4*R* with **9** and clearanol F.

Other eight known isobenzofuranones or isochromenones were elucidated by comparison of their NMR and MS data with reported literature, determined to be (*R*)-3-acetyl-7-hydroxy-5-methoxy-3,4-dimethylisobenzofuran-1(3*H*)-one (**5**) [6], (3*R*,3^1^*S*)-7-hydroxy-3-(1-hydroxyethyl)-5-methoxy-3,4-dimethylisobenzofuran-1(3*H*)-one (**6**) [6], dothideomynone A (**11**) [20], 3,8-dihydroxy-3-hydroxymethyl-6-methoxy-4,5-dimethylisochromen-l-one (**12**) [21], (*R*)-4,8-dihydroxy-6-methoxy-4,5-dimethyl-3-methyleneisochromen-1-one (**13**) [6], 6,8-dihydroxy-3,4-dimethylisocoumarin (**14**) [22], acremonone F (**15**) [23], acremonone G (**16**) [23], whose absolute configurations were assigned by comparison of optical rotation data and ECD with the reported data.

**Scheme 1:**
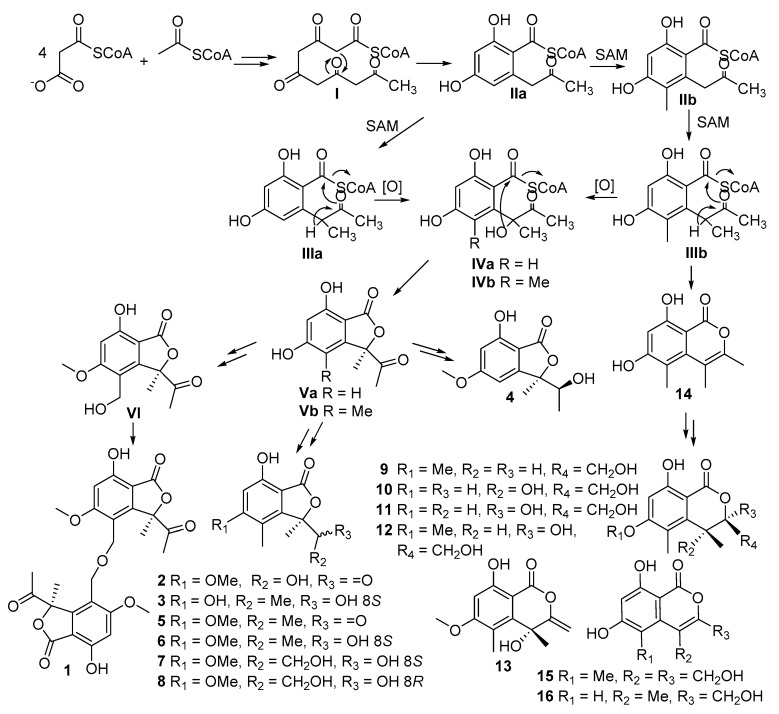
Plausible biosynthetic pathways for compounds **1**–**16**.

Fungal polyketides with structural diversity derived from the polymerization of short-chain carboxylic acids units are mainly synthesized by iterative type I polyketide synthases (IPKSs) via a series of decarboxylative condensations of malonyl-CoA extender units and *β*-keto modification [26,27,28]. Isobenzofuranones and isochromenones, suggested to share the same biogenetic pathway, could be hypothesized as derivatives from the pentaketide intermediate **I**. In brief, other intermediates (**IIa**–**VI**) originated from intermediate **I** via several steps as shown in Scheme 1 [29,30,31]. Compound **14** was obtained as an intermediate derived from intermediate **IIIb** by cyclization and dehydration, which then yielded a series of isochromenones (**9**–**13**, **15**–**16**) after several reaction steps such as methylation, reduction, oxidation, etc. Methylation and reduction of the ketone carbonyl of intermediate **Va** offered compound **4**. Besides, a succession of isobenzofuranones (**2**–**3**, **5**–**8**) was produced by derivatization of intermediate **Vb**. Interestingly, methylation and oxidation of the phenolic ring in intermediate **Vb** gave one important intermediate **VI**, which afterward formed a dimer, leptosphaerin J (**1**), via intermolecular condensation of alcoholic hydroxyls. To the best of our knowledge, leptosphaerin J (**1**) was isolated as a novel, rare, naturally-occurring isobenzofuranone dimer.

All of the obtained compounds (**1**–**16**) were evaluated for their cytotoxic and antiviral activities, according to previously reported methods [18,32]. The results showed that none of them displayed obvious cytotoxicity against three cancer cell lines, K562, MCF-7 and SGC7901 (IC_50_ > 50 μM). Meanwhile, all of the isobenzofuranones (**1**–**8**) and isochromenones (**9**–**16**) were inactive (IC_50_ > 100 μM) against H3N2, EV71 and HIV viruses.

Natural isobenzofuranones and isochromenones have been mostly reported from fungal sources, with modest or weak antimicrobial activities [6,19,23,25,29,33,34,35], and weak or no cytotoxicities [24,30,36]. Nevertheless, in those reported isochromenones, (*R*)-4,6,8-trihydroxy-4,5-dimethyl-3-methyleneisochromen-1-one and (*R*)-4,8-dihydroxy-6-methoxy-4,5-dimethyl-3-methyleneisochromen-1-one (**13**) were reported with antifungal activity against *Cochliobolus miyabeanus* with IC_50_ values of 10 and 0.5 μM [6], respectively. Additionally, **13** and clearanol C showed weak inhibitory activity against *Candida albicans* biofilm formation with MIC (minimum inhibitory concentration) values of 86 ± 3 and 101 ± 3 μM, respectively [19]. However, the three bioactive isochromenones mentioned above share the presence of an exocyclic olefinic group anchored at C-3, which probably plays a role in improving the antimicrobial activities.

## 3. Materials and Methods

### 3.1. General Experimental Procedures

Optical rotations were measured with a Perkin Elmer MPC 500 (Waltham, MA, USA) polarimeter. UV spectra were measured on a Shimadzu UV-2600 PC spectrometer (Shimadzu, Beijing, China). IR spectra were measured on an IR Affinity-1 spectrometer (Shimadzu, Beijing, China). Circular dichroism spectra were measured with a Chirascan circular dichroism spectrometer (Applied Photophysics, Ltd., Leatherhead, UK). 1D and 2D NMR spectra were recorded on a Bruker Avance 500 MHz and 700 MHZ NMR (Bruker, Fällanden, Switzerland) spectrometer using TMS as an internal standard, and chemical shifts were recorded as *δ*-values. HRESIMS were recorded on a Bruker miXis TOF-QII mass spectrometer (Bruker, Fällanden, Switzerland). X-ray diffraction intensity data were collected on an Agilent Xcalibur Nova single-crystal diffractometer (Santa Clara, CA, USA) using Cu K*α* radiation. TLC and column chromatography (CC) were performed on plates precoated with silica gel GF254 (10–40 μm) and over silica gel (200–300 mesh) (Qingdao Marine Chemical Factory, Qingdao, China), and Sephadex LH-20 (Amersham Biosciences, Uppsala, Sweden), respectively. Spots were detected on TLC (Qingdao Marine Chemical Ltd., Qingdao, China) under 254 nm UV light or by heating after spraying with 5% H_2_SO_4_ in EtOH. All solvents used were of analytical grade (Tianjin Fuyu Chemical and Industry Factory, Tianjin, China). Semi-preparative HPLC was performed using an ODS column (YMC-pack ODS-A, YMC Co. Ltd., Kyoto, Japan, 10 × 250 mm, 5 μm, 2.5 mL/min).

### 3.2. Fungal Material

The fungal strain SCSIO 41005 was isolated from deep-sea sediments, which were collected from the Indian Ocean (Lat: 7°9.43716667′ N, Long: 89°4.4266667′ E) at a depth of 3614 m in 2013. The isolates were stored on Muller Hinton broth (MB) agar (malt extract 15 g, sea salt 15 g and agar 15 g) slants at 4 °C, and a voucher specimen was deposited in the CAS Key Laboratory of Tropical Marine Bio-resources and Ecology, South China Sea Institute of Oceanology, Chinese Academy of Sciences, Guangzhou, China. Fungal identification was performed by analysis of its ITS region of the rDNA as described in the Supplementary Materials. The resulting sequence data, which were most similar (99%) to the sequence of *Leptosphaeria* sp. EIODSF012 (accession no. KJ173535), have been deposited in GenBank (accession no. KY290887). Thus, the strain was identified as *Leptosphaeria* sp. SCSIO 41005.

### 3.3. Fermentation, Extraction and Isolation

The strain *Leptosphaeria* sp. SCSIO 41005 was cultured on MB-agar plates at 25 °C for 7 days. The seed medium (malt extract 15 g and sea salt 2.5 g in 1.0 L tap distilled water, pH 7.4–7.8) was inoculated with strain SCSIO 41005 and incubated at 25 °C for 3 days on a rotating shaker (180 rpm). Then, large-scale fermentation of fungal isolate SCSIO 41005 was incubated for 90 days at room temperature in 1 L × 48 conical flasks with solid rice medium (each flask contained 200 g rice, 3 g sea salt and 200 mL naturally sourced water). The whole fermented cultures were harvested and extracted with acetone and then filtered through cheesecloth to separate them into filtrate and mycelia. The filtrate was concentrated under vacuum to remove the organic solvents and then extracted three times with EtOAc, while the mycelia were also extracted three times with EtOAc. Both EtOAc solutions were combined and concentrated under vacuum to yield a brown-colored EtOAc extract (180 g).

### 3.4. Purification

The crude EtOAc extract was subjected to silica gel column chromatography eluted with CH_2_Cl_2_/MeOH in a gradient eluent (*v*/*v*, 100:0, 98:2, 97:3, 95:5, 90:10, 80:20, 50:50) to obtain six fractions (fractions 1–6) based on TLC properties. Fraction 2 was purified by further Sephadex LH-20 (CH_2_Cl_2_/MeOH, 1:1) and then by semi-preparative reversed-phase HPLC (2 mL/min, detector UV λ_max_ 220 nm, MeOH/H_2_O 45:55) to yield **5** (19.3 mg) and **13** (15.9 mg). Fraction 3 was purified by further Sephadex LH-20 (CH_2_Cl_2_/MeOH, 1:1) and then by semi-preparative reverse-phase HPLC (2 mL/min, detector UV λ_max_ 220 nm, MeOH/H_2_O 39:61) to yield **1** (12.0 mg). Fraction 4 was applied to Sephadex LH-20 (CH_2_Cl_2_/MeOH, 1:1) to yield three subfractions. Fraction 4.2 was purified by semi-preparative reverse-phase HPLC (2 mL/min, detector UV λ_max_ 220 nm, MeOH/H_2_O 57:43) to yield **6** (508 mg) and **9** (4 mg). Fraction 4.3 was purified by semi-preparative reverse-phase HPLC (2 mL/min, detector UV λ_max_ 220 nm, MeOH/H_2_O 57:43) to yield **14** (3.0 mg). Fraction 5 was chromatographed on a silica gel column with a CH_2_Cl_2_/MeOH gradient system (from 1:0 to 0:1) to afford three subfractions. Fraction 5.1 was purified by semi-preparative reverse-phase HPLC (2 mL/min, detector UV λ_max_ 220 nm, MeCN/H_2_O 15:85) to yield **4** (22.0 mg). Fraction 5.2 was purified by semi-preparative reverse-phase HPLC (2 mL/min, detector UV λ_max_ 220 nm, MeCN/H_2_O 23:77) to yield **8** (41.0 mg), **12** (329 mg) and **16** (5.0 mg). Fraction 5.3 was purified by semi-preparative reverse-phase HPLC (2 mL/min, detector UV λ_max_ 220 nm, MeCN/H_2_O 22:78) to yield **10** (10.2 mg), **11** (104 mg) and **15** (2.8 mg). Fraction 6 was chromatographed on a silica gel column with a CH_2_Cl_2_/MeOH gradient system (from 1:0 to 0:1) to afford three subfractions. Fraction 6.1 was purified by semi-preparative reverse-phase HPLC (2 mL/min, detector UV λ_max_ 220 nm, MeCN/H_2_O 21:79) to yield **2** (4.0 mg). Fraction 6.2 was purified by semi-preparative reverse-phase HPLC (2 mL/min, detector UV λ_max_ 220 nm, MeOH/H_2_O 26:74) to yield **3** (59.1 mg) and **7** (1.12 g).

### 3.5. Spectral Data

*Leptosphaerin J* (**1**). Colorless crystals; ${\left[\alpha \right]}_{D}^{25}$ = +87.3° (*c* 0.06, MeOH); UV (MeOH) λ_max_ (log *ε*) 299 (2.79), 260 (3.19), 222 (3.63) nm; CD (4.61 × 10^−4^ M, MeOH) λ_max_ (Δ*ε*) 261 (+14.79), 222 (−38.61) nm; IR (film) ν_max_ 3400, 2937, 1708, 1562, 1217, 1051 cm^−1^; HRESIMS (positive) [M + Na]^+^ *m*/*z* 537.1361, calcd. for C_26_H_26_O_11_Na^+^. ^1^H and ^13^C NMR spectrum data are seen in Table 1.

*Leptosphaerin K* (**2**). Colorless oil; ${\left[\alpha \right]}_{D}^{25}$ = +21.0° (*c* 0.10, MeOH); UV (MeOH) λ_max_ (log *ε*) 300 (3.51), 259 (3.81), 220 (4.20) nm; CD (5.67 × 10^−4^ M, MeOH) λ_max_ (Δ*ε*) 259 (+4.50), 234 (+9.00), 221 (−14.33) nm; IR (film) ν_max_ 3448, 2972, 1714, 1645, 1205, 1028 cm^−1^; HRESIMS (negative) [M − H]^−^ *m*/*z* 251.0565, calcd. for C_12_H_11_O_6_^−^, 251.0556; [2M − H]^−^ *m*/*z* 503.1204, calcd. for C_24_H_23_O_12_^−^, 503.1190. ^1^H and ^13^C NMR spectrum data are seen in Table 1.

*Leptosphaerin L* (**3**). Colorless oil; ${\left[\alpha \right]}_{D}^{25}$ = −44.7° (*c* 0.15, MeOH); UV (MeOH) λ_max_ (log *ε*) 299 (3.76), 259 (4.02), 220 (4.28) nm; CD (9.15 × 10^−4^ M, MeOH) λ_max_ (Δ*ε*) 260 (+1.53), 212 (−5.56) nm; IR (film) ν_max_ 3302, 2981, 1708, 1562, 1244, 1055 cm^−1^; HRESIMS (negative) [M − H]^−^ *m*/*z* 237.0775, calcd. for C_12_H_13_O_5_^−^, 237.0763; [2M − H]^−^ *m*/*z* 475.1605, calcd. for C_24_H_27_O_10_^−^, 475.1604. ^1^H and ^13^C NMR spectrum data are seen in Table 1.

*Leptosphaerin M* (**4**). Colorless oil; ${\left[\alpha \right]}_{D}^{25}$ = −32.6° (*c* 0.10, MeOH); UV (MeOH) λ_max_ (log *ε*) 291 (3.58), 257 (4.06), 220 (4.24) nm; CD (6.30 × 10^−4^ M, MeOH) λ_max_ (Δ*ε*) 258 (+1.69), 213 (−6.41) nm; IR (film) ν_max_ 3358, 2943, 1726, 1610, 1209, 1020 cm^−1^; HRESIMS (negative) [M − H]^−^ *m*/*z* 237.0775, calcd. for C_12_H_13_O_5_^−^, 237.0763; [M + Cl]^−^ *m*/*z* 273.0530, calcd. for C_12_H_14_O_5_Cl^−^, 273.0530; [2M − H]^−^ *m*/*z* 475.1605, calcd. for C_24_H_27_O_10_^−^, 475.1604. ^1^H and ^13^C NMR spectrum data are seen in Table 1.

*Clearanol I* (**9**). Colorless crystals; ${\left[\alpha \right]}_{D}^{25}$ = +18.2° (*c* 0.19, MeOH); UV (MeOH) λ_max_ (log *ε*) 308 (3.86), 265 (4.11), 229 (4.10) nm; CD (1.10 × 10^−3^ M, MeOH) λ_max_ (Δ*ε*) 309 (−0.94), 270 (+4.71), 250 (+0.60), 237 (+4.56), 224 (−2.85) nm; IR (film) ν_max_ 3419, 2924, 1651, 1616, 1244, 1165 cm^−1^; HRESIMS (positive) [M + Na]^+^ *m*/*z* 275.0890, calcd. for C_13_H_16_O_5_Na^+^, 275.0895; [M + H]^+^ *m*/*z* 253.1077; calcd. for C_13_H_17_O_5_^+^, 253.1076. ^1^H and ^13^C NMR spectrum data are seen in Table 2.

*Clearanol J* (**10**). Colorless crystals; ${\left[\alpha \right]}_{D}^{25}$ = + 17.4° (*c* 0.10, MeOH); UV (MeOH) λ_max_ (log *ε*) 314 (3.74), 271 (3.92), 229 (3.97), 216 (4.20) nm; CD (7.71 × 10^−4^ M, MeOH) λ_max_ (Δ*ε*) 276 (+0.07), 258 (−0.12), 242 (+0.29), 230 (−0.62), 211 (+1.64) nm; IR (film) ν_max_ 3334, 2937, 1645, 1367, 1253, 1014 cm^−1^; HRESIMS (negative) [M − H]^−^ *m*/*z* 253.0721, calcd. for C_12_H_13_O_6_^−^, 253.0712; [M + Cl]^−^ *m*/*z* 289.0486, calcd. for C_12_H_14_O_6_Cl^−^, 289.0479. ^1^H and ^13^C NMR spectrum data are seen in Table 2.

### 3.6. X-ray Crystal Structure Analysis

Crystallographic data for compounds leptosphaerin J (**1**), clearanol E (**7**) and clearanol I (**9**) were collected on an Agilent Xcalibur Nova single-crystal diffractometer using Cu K*α* radiation. The structures of these compounds were solved by direct methods (SHELXS97), expanded using difference Fourier technniques, and refined by full-matrix least-squares calculation. The non-hydrogen atoms were refined anisotropically, and hydrogen atoms were fixed at calculated positions. Crystallographic data for the structures of leptosphaerin J (**1**), clearanol E (**7**) and clearanol I (**9**) have been deposited in the Cambridge Crystallographic Data Centre database (deposition number CCDC 1539529, CCDC 1539538, CCDC 1539537). Copies of the data can be obtained free of charge from the CCDC at www.ccdc.cam.ac.uk.

Crystal data for **1**: C_26_H_26_O_11_, *M* = 514.47, Orthorhombic, a = 23.08124(16) Å, b = 10.49438(8) Å, c = 10.03167(10) Å, a = 90°, b = 90°, g = 90°, V = 2429.90 (3) Å^3^, T = 150(2) K, space group *P*2_1_2_1_2_1_, Z = 4, *μ*(Cu K*α*) = 0.938 mm^−1^, 23,207 reflections measured, 4841 independent reflections (Rint = 0.0236). The final R_1_ values were 0.0289 (I > 2σ(I)). The final *wR* (F^2^) values were 0.0297 (all data). The goodness of fit on F^2^ was 1.031. Flack parameter = 0.00 (5).

Crystal data for **7**: C_13_H_16_O_6_, *M* = 268.26, Orthorhombic, a = 12.50776(10) Å, b = 13.73220(10) Å, c = 14.93010(10) Å, a = 90°, b = 90°, g = 90°, V = 2564.38 (3) Å^3^, T = 150(2) K, space group *P*2_1_2_1_2_1_, Z = 8, *μ*(Cu K*α*) = 0.937 mm^−1^, 19,111 reflections measured, 5109 independent reflections (Rint = 0.0366). The final R_1_ values were 0.0312 (I > 2σ (I)). The final *wR* (F^2^) values were 0.0321 (all data). The goodness of fit on F^2^ was 1.052. Flack parameter = 0.04 (8).

Crystal data for **9**: C_13_H_16_O_5_, *M* = 252.26, Orthorhombic, a = 4.91205(6) Å, b = 13.99641(14) Å, c = 17.18834(19) Å, a = 90°, b = 90°, g = 90°, V= 1181.72 (2) Å^3^, T= 150(2) K, space group *P*2_1_2_1_2_1_, Z = 4, *μ*(Cu K*α*) = 0.914 mm^−1^, 8470 reflections measured, 2216 independent reflections (Rint = 0.0181). The final R_1_ values were 0.0256 (I > 2σ (I)). The final *wR* (F^2^) values were 0.0259 (all data). The goodness of fit on F^2^ was 1.059. Flack parameter = −0.03 (5).

### 3.7. Cytotoxic Bioassay

All isolated compounds were evaluated against the three human tumor cell lines (K562, MCF-7 and SGC7901) guided by previously reported methods [17,18]. Taxol was used as the positive control with IC_50_ values of 3.67, 7.56 and 3.41 nM against K562, MCF-7 and SGC7901, respectively.

### 3.8. Antiviral Assay against H3N2, EV71 and HIV Viruses

The antiviral assay against H3N2 was evaluated by the cytopathic effect (CPE) inhibition assay, on Madin–Darby canine kidney (MDCK) cells with CCK-8 assay, while EV71 virus was measured by the ability to inhibit the CPE induced by EV71 virus on Vero cells with a CCK-8 assay, according to previously reported methods [18]. The 50% inhibitory concentration (IC_50_) of the testing compound was calculated using the GraphPad Prism software. Oseltamivir was used as the positive control in an anti-H3N2 assay with IC_50_ values of 36.8 nM, and ribavirin was used as the positive control in an anti-EV71 assay with an IC_50_ value of 0.60 μM. In addition, all isolated compounds were evaluated for their inhibitory activities against infection with HIV-1 SF162 in TZM-bl cells, according to previously reported methods [32]. A nucleoside reverse transcriptase inhibitor, abacavir, was used as the positive control with an IC_50_ value of 0.8 μM.

## 4. Conclusions

The chemical investigation of the deep-sea-derived fungus *Leptosphaeria* sp. SCSIO 41005 has led to four new isobenzofuranones and two new isochromenones, together with other ten known analogues. Leptosphaerin J (**1**) presents a rare naturally-occurring centrosymmetric isobenzofuranone dimer skeleton with an ether bond. The absolute configurations of these compounds were determined by ECD and optical rotation comparison with those reported data, some of which were further confirmed by single crystal X-ray diffraction experiments, including one pair of epimers (**7** and **8**) with known planar structures. However, no compounds exhibited any significant activities in the bioassay screening of cytotoxicity towards K562, MCF-7 and SGC7901 and anti-virusal activity against EV71, H3N2 and HIV.

## Figures and Tables

**Table 1:** The ^1^H and ^13^C NMR data of compounds **1**–**4** (MeOD, *δ* in ppm, *J* in Hz).

Position	1		2		3		4	
	*δ*_C_^b^, Type	*δ*_H_^a^, Mult.	*δ*_C_^d^, Type	*δ*_H_^c^, Mult.	*δ*_c_^b^, type	*δ*_H_^a^, Mult.	*δ*_C_^b^, Type	*δ*_H_^a^, Mult.
**1**	170.5, C	-	173.2, C	-	172.3, C	-	171.5, C	-
**3**	90.5, C	-	90.0, C	-	91.5, C	-	90.0, C	-
**4**	114.5, C	-	113.1, C	-	111.4, C	-	100.1, CH	6.51, s
**5**	166.7, C	-	166.2, C	-	165.1, C	-	168.4, C	-
**6**	100.8, CH	6.59, s	99.4, CH	6.38, s	103.1, CH	6.36, s	102.5, CH	6.39, s
**7**	160.3, C	-	158.1, C	-	157.5, C	-	160.4, C	-
**8**	202.9, C	-	174.5, C	-	71.0, CH	4.22, q (6.5)	72.4, CH	3.90, q (6.5)
**9**	23.8, CH_3_	1.94, s		-	17.3, CH_3_	0.85, d (6.5)	17.4, CH_3_	1.05, d (6.5)
**10**	21.1, CH_3_	1.69, s	22.8, CH_3_	1.78, s	21.5, CH_3_	1.71, s	22.0, CH_3_	1.59, s
**11**	65.1, CH_2_	4.43, d (10.9)	10.6, CH_3_	2.16, s	11.3, CH_3_	2.09, s	-	-
		4.25, d (10.9)						
**12**	57.0, CH_3_	3.90, s	56.5, CH_3_	3.82, s	-	-	56.4, CH_3_	3.84, s
**3a**	152.1, C	-	153.3, C	-	153.7, C	-	153.7, C	-
**7a**	104.8, C	-	104.9, C	-	104.1, C	-	104.1, C	-

^a^ Recorded at 500 MHz; ^b^ Recorded at 125 MHz; ^c^ Recorded at 700 MHz; ^d^ Recorded at 175 MHz; Mult.: multiple.

**Table 2:** The ^1^H and ^13^C NMR data of compounds **9** and **10** (MeOD, *δ* in ppm, *J* in Hz).

Position	9		10	
	*δ*_C_^b^, Type	*δ*_H_^a^, Mult.	*δ*_C_^d^, Type	*δ*_H_^c^, Mult.
**1**	171.6, C	-	171.5, C	-
**3**	82.7, CH	4.50, overlapped	86.1, CH	4.26, dd (2.1, 7.7)
**4**	31.7, CH	3.29, overlapped	71.7, C	-
**5**	115.3, C	-	116.2, C	-
**6**	166.1, C	-	165.9, C	-
**7**	98.3, CH	6.43, s	102.2, CH	6.28, s
**8**	164.5, C	-	163.9, C	-
**9**	62.5, CH_2_	3.90, dd (7.0, 11.5)	60.9, CH_2_	4.06, dd (2.1, 11.9)
		3.80, dd (5.5, 11.5)		3.85, dd (7.7, 11.9)
**10**	12.8, CH_3_	1.07, d (7.0)	20.6, CH_3_	1.37, s
**11**	10.1, CH_3_	2.07, s	12.2, CH_3_	2.29, s
**12**	56.4, CH_3_	3.86, s	-	-
**4a**	146.1, C	-	147.9, C	-
**8a**	100.7, C	-	99.5, C	-

^a^ Recorded at 500 MHz; ^b^ Recorded at 125 MHz; ^c^ Recorded at 700 MHz; ^d^ Recorded at 175 MHz; Mult.: multiple.

## Supplementary Materials

The following are available online at www.mdpi.com/1660-3397/15/7/204/s1. This section includes 1D, 2D NMR spectra and other spectroscopic and spectrometric data for new compounds (**1**–**4**, **9**–**10**), the ECD and UV curves of the most important compounds, X-ray crystallographic files of compounds **1**, **7** and **9**, the physicochemical data of these known compounds and the strain’s ITS sequence of the rDNA. Supplementary data associated with this article can be found in the online version.

## References

[1] Blunt J.W., Copp B.R., Keyzers R.A., Munro M.H.G., Prinsep M.R. (2017). Marine natural products. Nat. Prod. Rep..

[2] Skropeta D., Wei L. (2014). Recent advances in deep-sea natural products. Nat. Prod. Rep..

[3] Lin J., Wang R., Xu G., Ding Z., Zhu X., Li E., Liu L. (2017). Two new polyketides from the ascomycete fungus *Leptosphaeria* sp.. J. Antibiot..

[4] Hashimoto M., Tsushima T., Murakami T., Nomiya M., Takada N., Tanaka K. (2008). Spiroleptosphol isolated from *Leptosphaeria doliolum*. Bioorg. Med. Chem. Lett..

[5] Murakami T., Tsushima T., Takada N., Tanaka K., Nihei K., Miura T., Hashimoto M. (2009). Four analogues of spiroleptosphol isolated from *Leptosphaeria doliolum*. Bioorg. Med. Chem..

[6] Tayone W.C., Honma M., Kanamaru S., Noguchi S., Tanaka K., Nehira T., Hashimoto M. (2011). Stereochemical investigations of isochromenones and isobenzofuranones isolated from *Leptosphaeria* sp. KTC 727. J. Nat. Prod..

[7] Lin J., Liu S., Sun B., Niu S., Li E., Liu X., Che Y. (2010). Polyketides from the Ascomycete Fungus *Leptosphaeria* sp.. J. Nat. Prod..

[8] Yamada T., Iwamoto C., Yamagaki N., Yamanouchi T., Minoura K., Yamori T., Uehara Y., Andoh T., Umemura K., Numata A. (2002). Leptosins M–N1, cytotoxic metabolites from a *Leptosphaeria* species separated from a marine alga. Structure determination and biological activities. Tetrahedron.

[9] Takahashi C., Numata A., Ito Y., Matsumura E., Araki H., Iwaki H., Kushida K. (1994). Leptosins, antitumour metabolites of a fungus isolated from a marine alga. J. Chem. Soc. Perkin Trans. 1.

[10] Takahashi C., Minoura K., Yamada T., Numata A., Kushida K., Shingu T., Hagishita S., Nakai H., Sato T., Harada H. (1995). Potent cytotoxic metabolites from a *Leptosphaeria* species. Structure determination and conformational analysis. Tetrahedron.

[11] Pedras M.S.C., Yu Y. (2008). Stress-driven discovery of metabolites from the phytopathogenic fungus *Leptosphaeria maculans*: Structure and activity of leptomaculins A–E. Bioorg. Med. Chem..

[12] Pedras M.S.C., Yu Y. (2008). Structure and biological activity of maculansin A, a phytotoxin from the phytopathogenic fungus *Leptosphaeria maculans*. Phytochemistry.

[13] Soledade M., Pedras M.S.C., Chumala P.B., Venkatesham U. (2005). New sesquiterpenic phytotoxins establish unprecedented relationship between different groups of blackleg fungal isolates. Bioorg. Med. Chem..

[14] Dahiya J.S. (1991). Pentacyclic triterpenes from the fungus, *Leptosphaeria maculans*. Phytochemistry.

[15] Chen S., Wang J., Lin X., Zhao B., Wei X., Li G., Kaliaperumal K., Liao S., Yang B., Zhou X. (2016). Chrysamides A-C, three dimeric nitrophenyl trans-Epoxyamides produced by the deep-sea-derived fungus *Penicillium chrysogenum* SCSIO 41001. Org. Lett..

[16] Tian Y., Lin X., Wang Z., Zhou X., Qin X., Kaliyaperumal K., Zhang T., Tu Z., Liu Y. (2015). Asteltoxins with antiviral activities from the marine sponge-derived fungus *Aspergillus* sp. SCSIO XWS02F40. Molecules.

[17] Luo X., Zhou X., Lin X., Qin X., Zhang T., Wang J., Tu Z., Yang B., Liao S., Tian Y. (2017). Antituberculosis compounds from a deep-sea-derived fungus *Aspergillus* sp. SCSIO Ind09F01. Nat. Prod. Res..

[18] Fang W., Lin X., Zhou X., Wan J., Lu X., Yang B., Ai W., Lin J., Zhang T., Tu Z. (2014). Cytotoxic and antiviral nitrobenzoyl sesquiterpenoids from the marine-derived fungus *Aspergillus ochraceus* Jcma1F17. MedChemComm.

[19] Gerea A.L., Branscum K.M., King J.B., You J., Powell D.R., Miller A.N., Spear J.R., Cichewicz R.H. (2012). Secondary metabolites produced by fungi derived from a microbial mat encountered in an iron-rich natural spring. Tetrahedron Lett..

[20] Hewage R.T., Aree T., Mahidol C., Ruchirawat S., Kittakoop P. (2014). One strain-many compounds (OSMAC) method for production of polyketides, azaphilones, and an isochromanone using the endophytic fungus *Dothideomycete* sp.. Phytochemistry.

[21] Tayone W.C., Kanamaru S., Honma M., Tanaka K., Nehira T., Hashimoto M. (2011). Absolute stereochemistry of novel isochromanone derivatives from *Leptosphaeria* sp. KTC 727. Biosci. Biotechnol. Biochem..

[22] Furuta T., Fukuyama Y., Asakawa Y. (1986). Polygonolide, an isocoumarin from *polygonum hydropiper* possessing antiinflammatory activity. Phytochemistry.

[23] Rukachaisirikul V., Rodglin A., Sukpondma Y., Phongpaichit S., Buatong J., Sakayaroj J. (2012). Phthalide and isocoumarin derivatives produced by an *Acremonium* sp. isolated from a mangrove *Rhizophora apiculata*. J. Nat. Prod..

[24] Song Y., Wang J., Li S., Cheng B., Li L., Chen B., Liu L., Lin Y., Gu Y. (2012). Metabolites of the mangrove fungus *Xylaria* sp. BL321 from the South China Sea. Planta Med..

[25] El-Elimat T., Raja H.A., Figueroa M., Falkinham J.O., Oberlies N.H. (2014). Isochromenones, isobenzofuranone, and tetrahydronaphthalenes produced by *Paraphoma radicina*, a fungus isolated from a freshwater habitat. Phytochemistry.

[26] Chooi Y., Tang Y. (2012). Navigating the fungal polyketide chemical space: from genes to molecules. J. Org. Chem..

[27] Zabala A.O., Chooi Y., Choi M.S., Lin H.C., Tang Y. (2014). Fungal polyketide synthase product chain-length control by partnering thiohydrolase. ACS Chem. Biol..

[28] Zhou H., Li Y., Tang Y. (2010). Cyclization of aromatic polyketides from bacteria and fungi. Nat. Prod. Rep..

[29] Prompanya C., Dethoup T., Gales L., Lee M., Pereira J.A., Silva A.M., Pinto M.M., Kijjoa A. (2016). New polyketides and new benzoic acid derivatives from the marine sponge-associated fungus *Neosartorya quadricincta* KUFA 0081. Mar. Drugs.

[30] Pérez Hemphill C.F., Daletos G., Liu Z., Lin W., Proksch P. (2016). Polyketides from the mangrove-derived fungal endophyte *Pestalotiopsis clavispora*. Tetrahedron Lett..

[31] Chinworrungsee M., Kittakoop P., Isaka M., Chanphen R., Tanticharoen M., Thebtaranonth Y. (2002). Halorosellins A and B, unique isocoumarin glucosides from the marine fungus *Halorosellinia oceanica*. J. Chem. Soc. Perkin Trans. 1.

[32] Zhou X., Fang W., Tan S., Lin X., Xun T., Yang B., Liu S., Liu Y. (2016). Aspernigrins with anti-HIV-1 activities from the marine-derived fungus *Aspergillus niger* SCSIO Jcsw6F30. Bioorg. Med. Chem. Lett..

[33] Saito T., Itabashi T., Wakana D., Takeda H., Yaguchi T., Kawai K., Hosoe T. (2016). Isolation and structure elucidation of new phthalide and phthalane derivatives, isolated as antimicrobial agents from *Emericella* sp. IFM57991. J. Antibiot..

[34] Qi J., Shao C., Li Z., Gan L., Fu X., Bian W., Zhao H., Wang C. (2013). Isocoumarin derivatives and benzofurans from a sponge-derived *Penicillium* sp. fungus. J. Nat. Prod..

[35] Ding G., Liu S., Guo L., Zhou Y., Che Y. (2008). Antifungal metabolites from the plant endophytic fungus *Pestalotiopsis foedan*. J. Nat. Prod..

[36] Boonyaketgoson S., Trisuwan K., Bussaban B., Rukachaisirikul V., Phongpaichit S. (2015). Isochromanone derivatives from the endophytic fungus *Fusarium* sp. PDB51F5. Tetrahedron Lett..

