# Supplementary material for: Isobenzofuranones and Isochromenones from the Deep-Sea Derived Fungus Leptosphaeria sp. SCSIO 41005
Source: Mar Drugs. 2017 Jun 29;15(7):204. doi: 10.3390/md15070204 (PMC5532646; doi:10.3390/md15070204)
Supplement: Supplementary file 1 [file marinedrugs-15-00204-s001.pdf]

Supplementary Materials

Isobenzofuranones and Isochromenones from the Deep-Sea Derived Fungus *Leptosphaeria* sp. SCSIO 41005

Xiaowei Luo [1,2], Xiuping Lin [1], Limbadri Salendra [1,2] , Xiaoyan Pang [1,2], Yu Dai [1,2], Bin Yang [1], Juan Liu [1], Junfeng Wang [1], Xuefeng Zhou [1,*], and Yonghong Liu [1,*]

[1] Chinese Academy of Sciences (CAS) Key Laboratory of Tropical Marine Bio-resources and Ecology / Guangdong Key Laboratory of Marine Materia Medica, South China Sea Institute of Oceanology, CAS, Guangzhou 510301, China
Emails: luoxiaowei14@mails.ucas.ac.cn (X.L.); xiupinglin@hotmail.com (X.L.); raj.badri202@gmail.com (L.S.); luckygirlpxy@163.com (X.P.); daiyu15@mails.ucas.ac.cn (Y.D.); bingo525@163.com (B.Y.); liujuan@scsio.ac.cn (J.L.); junfeng1982a@163.com (J.W.)

[2] University of Chinese Academy of Sciences, Beijing 100049, China

* Correspondence: xfzhou@scsio.ac.cn (X.Z.); yonghongliu@scsio.ac.cn (Y.L.); Tel: +86-020-89023174 (X.Z.)

Contents of Supporting Information

No.	Contents	page
1	**Figure S1**. ^{1}H NMR spectrum of leptosphaerin J (**1**) (MeOD)	5
2	**Figure S2.** ^{13}C NMR and DEPT spectrum of leptosphaerin J (**1**) (MeOD)	5-6
3	**Figure S3**. HSQC spectrum of leptosphaerin J (**1**) (MeOD)	6
4	**Figure S4.** HMBC spectrum of leptosphaerin J (**1**) (MeOD)	7
5	**Figure S5.** ^{1}H-^{1}H COSY spectrum of leptosphaerin J (**1**) (MeOD)	7
6	**Figure S6.** Negative HRESIMS spectrum of leptosphaerin J (**1**)	8
7	**Figure S7.** Positive HRESIMS spectrum of leptosphaerin J (**1**)	8
8	**Figure S8.** UV spectrum of leptosphaerin J (**1**)	9
9	**Figure S9.** IR spectrum of leptosphaerin J (**1**)	10
10	**Figure S10.** UV and CD spectrum of leptosphaerin J (**1**)	10-11
11	**Figure S11.** ^{1}H NMR spectrum of leptosphaerin K (**2**) (MeOD)	11
12	**Figure S12.** ^{13}C NMR and DEPT spectrum of leptosphaerin K (**2**) (MeOD)	12
13	**Figure S13.** HSQC spectrum of leptosphaerin K (**2**) (MeOD)	13
14	**Figure S14.** HMBC spectrum of leptosphaerin K (**2**) (MeOD)	13
15	**Figure S15.** HRESIMS spectrum of leptosphaerin K (**2**)	14
16	**Figure S16.** UV spectrum of leptosphaerin K (**2**)	15
17	**Figure S17.** IR spectrum of leptosphaerin K (**2**)	16
18	**Figure S18.** UV and CD spectrum of leptosphaerin K (**2**)	16-17
19	**Figure S19.** ^{1}H NMR spectrum of leptosphaerin L (**3**) (MeOD)	17
20	**Figure S20.** ^{13}C NMR and DEPT spectrum of leptosphaerin L (**3**) (MeOD)	18
21	**Figure S21.** HSQC spectrum of leptosphaerin L (**3**) (MeOD)	19
22	**Figure S22.** HMBC spectrum of leptosphaerin L (**3**) (MeOD)	19
23	**Figure S23.** HRESIMS spectrum of leptosphaerin L (**3**)	20
24	**Figure S24.** UV spectrum of leptosphaerin L (**3**)	20
25	**Figure S25.** IR spectrum of leptosphaerin L (**3**)	21
26	**Figure S26.** UV and CD spectrum of leptosphaerin L (**3**)	21-22
27	**Figure S27.** ^{1}H NMR spectrum of leptosphaerin M (**4**) (MeOD)	22
28	**Figure S28.** ^{13}C NMR and DEPT spectrum of leptosphaerin M (**4**) (MeOD)	23
29	**Figure S29.** HSQC spectrum of leptosphaerin M (**4**) (MeOD)	24
30	**Figure S30.** HMBC spectrum of leptosphaerin M (**4**) (MeOD)	24
31	**Figure S31.** HRESIMS spectrum of leptosphaerin M (**4**)	25
32	**Figure S32.** UV spectrum of leptosphaerin M (**4**)	26
33	**Figure S33.** IR spectrum of leptosphaerin M (**4**)	27
34	**Figure S34.** UV and CD spectrum of leptosphaerin M (**4**)	27-28
35	**Figure S35.** ^{1}H NMR spectrum of clearanol I (**9**) (MeOD)	28
36	**Figure S36.** ^{13}C NMR and DEPT spectrum of clearanol I (**9**) (MeOD)	29
37	**Figure S37.** HSQC spectrum of clearanol I (**9**) (MeOD)	30
38	**Figure S38.** HMBC spectrum of clearanol I (**9**) (MeOD)	30
39	**Figure S39.** ^{1}H-^{1}H COSY spectrum of clearanol I (**9**) (MeOD)	31

40	**Figure S40.** NOESY spectrum of clearanol I (**9**) (MeOD)	31
41	**Figure S41.** HRESIMS spectrum of clearanol I (**9**)	32
**No.**	**Contents**	**page**
42	**Figure S42.** UV spectrum of clearanol I (**9**)	32
43	**Figure S43.** IR spectrum of clearanol I (**9**)	33
44	**Figure S44.** UV and CD spectrum of clearanol I (**9**)	33-34
45	**Figure S45.** ^{1}H NMR spectrum of clearanol J (**10**) (MeOD)	34
46	**Figure S46.** ^{13}C NMR and DEPT spectrum of clearanol J (**10**) (MeOD)	35
47	**Figure S47.** HSQC spectrum of clearanol J (**10**) (MeOD)	36
48	**Figure S48.** HMBC spectrum of clearanol J (**10**) (MeOD)	36
49	**Figure S49.** ^{1}H-^{1}H COSY spectrum of clearanol J (**10**) (MeOD)	37
50	**Figure S50.** NOESY spectrum of clearanol J (**10**) (MeOD)	37
51	**Figure S51.** HRESIMS spectrum of clearanol J (**10**)	38
52	**Figure S52.** UV spectrum of clearanol J (**10**)	38
53	**Figure S53.** IR spectrum of clearanol J (**10**)	39
54	**Figure S54.** UV and CD spectrum of clearanol J (**10**)	39-40
55	**Figure S55.** UV and CD spectrum of (*R*)-3-acetyl-7-hydroxy-5-methoxy-3,4-dimethylisobenzofuran-1(3*H*)-one (**5**)	40-41
56	**Figure S56.** UV and CD spectrum of (3*R*,3^1*S*)-7-hydroxy-3-(1-hydroxyethyl)-5-methoxy-3,4-dimethylisobenzofuran-1(3*H*)-one (**6**)	41-42
57	**Figure S57.** UV and CD spectrum of clearanol E (**7**)	43
58	**Figure S58.** UV and CD spectrum of clearanol D (**8**)	44
59	**Figure S59.** UV and CD spectrum of dothideomynone A (**11**)	45
60	**Figure S60.** X-Ray structure of leptosphaerin J (**1**)	46-47
61	**Table S1.** Crystal data and structure refinement for leptosphaerin J (**1**)	47-48
62	**Table S2.** Atomic coordinates (x 10^4) and equivalent isotropic displacement parameters (Å^{2}x 10^3) for leptosphaerin J (**1**). U (eq) is defined as one third of the trace of the orthogonalized U^{ij} tensor.	48-49
63	**Table S3**. Bond lengths [Å] and angles [°] for leptosphaerin J (**1**)	50-54
64	**Table S4**. Anisotropic displacement parameters (Å^{2}x 10^3) for leptosphaerin J (**1**). The anisotropic displacement factor exponent takes the form: $-2\pi^2$[h^2 $a^{*2}U^{11}$ + ... + 2 h k a* b* U^{12}]	55-56
65	**Table S5**. Hydrogen coordinates (x 10^4) and isotropic displacement parameters (Å^{2}x 10 ^{3}) for leptosphaerin J(**1**).	56
66	**Table S6**. Torsion angles [°] for leptosphaerin J (**1**).	57-59
67	**Table S7**. Hydrogen bonds for leptosphaerin J (**1**) [Å and °].	59
68	**Figure S61.** X-Ray structure of clearanol E (**7**)	60-61
69	**Table S8**. Crystal data and structure refinement for clearanol E (**7**)	61-62
70	**Table S9**. Atomic coordinates (x 10^4) and equivalent isotropic displacement parameters (Å^{2}x 10^3) for clearanol E (**7**). U(eq) is defined as one third of the trace of the orthogonalized U^{ij} tensor.	63-64
71	**Table S10**. Bond lengths [Å] and angles [°] for clearanol E (**7**)	64-69
72	**Table S11**. Anisotropic displacement parameters (A^2 x 10^3) for clearanol E	69-70

(**7**).The anisotropic displacement factor exponent takes the form: -2 pi^2 [h^2 a*^2 U11 + ... + 2 h k a* b* U12]

No.	Contents	page
73	**Table S12**. Hydrogen coordinates (x 10^4) and isotropic displacement parameters (A^2 x 10^3) for clearanol E (**7**)	70-71
74	**Table S13**. Torsion angles [°] for clearanol E (**7**)	71-73
75	**Table S14**. Hydrogen bonds for clearanol E (**7**) [Å and °].	74
76	**Figure S62.** X-Ray structure of clearanol I (**9**)	74-75
77	**Table S15**. Crystal data and structure refinement for clearanol I (**9**)	75-76
78	**Table S16**. Atomic coordinates (x 10^4) and equivalent isotropic displacement parameters (A^2 x 10^3) for clearanol I (**9**). U(eq) is defined as one third of the trace of the orthogonalized Uij tensor.	77
78	**Table S17**. Bond lengths [Å] and angles [°] for clearanol I (**9**).	77-80
80	**Table S18**. Anisotropic displacement parameters (A^2 x 10^3) for clearanol I (**9**). The anisotropic displacement factor exponent takes the form: -2 pi^2 [h^2 a*^2 U11 + ... + 2 h k a* b* U12]	81
81	**Table S19**. Hydrogen coordinates (x10^4) and isotropic displacement parameters (A^2 x 10^3) for clearanol I (**9**).	81-82
82	**Table S20**. Torsion angles [°] for clearanol I (**9**).	82-83
83	**Table S21**. Hydrogen bonds for clearanol I (**9**) [Å and °].	83
84	The physicochemical data of the known compounds	84-85
85	The strain's (*Leptosphaeria* sp. SCSIO 41005) ITS sequence of the rDNA	85-86

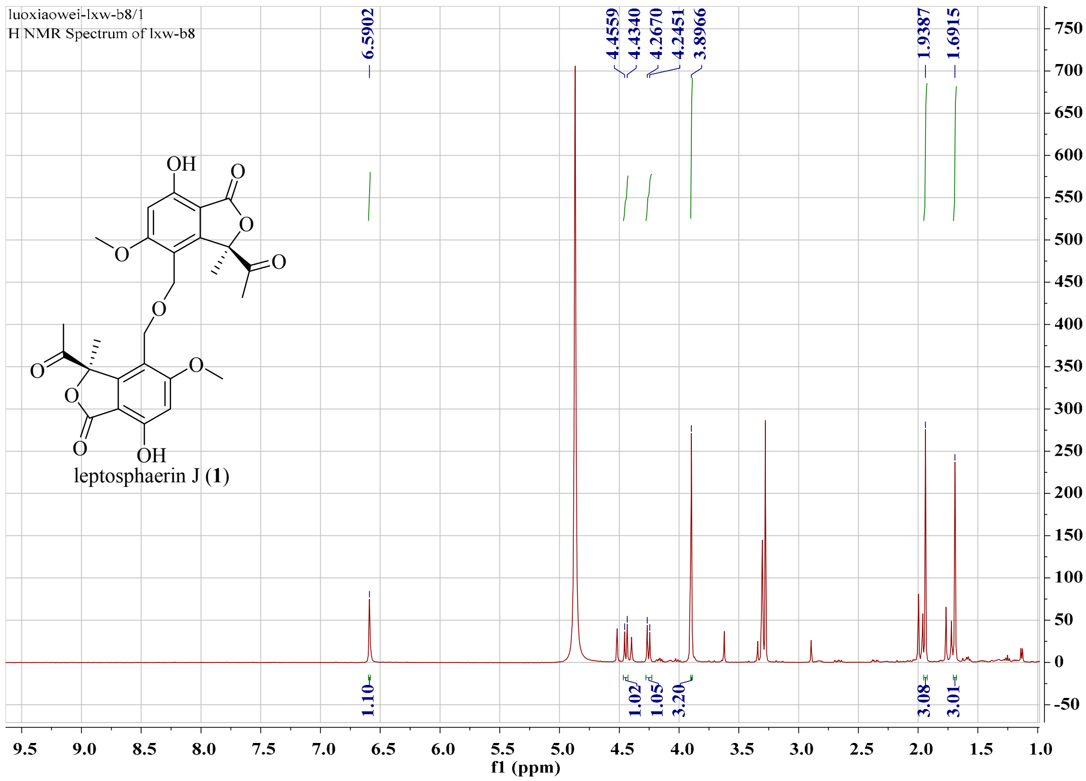

Figure S1. ^{1}H NMR spectrum of leptosphaerin J (**1**) (MeOD)

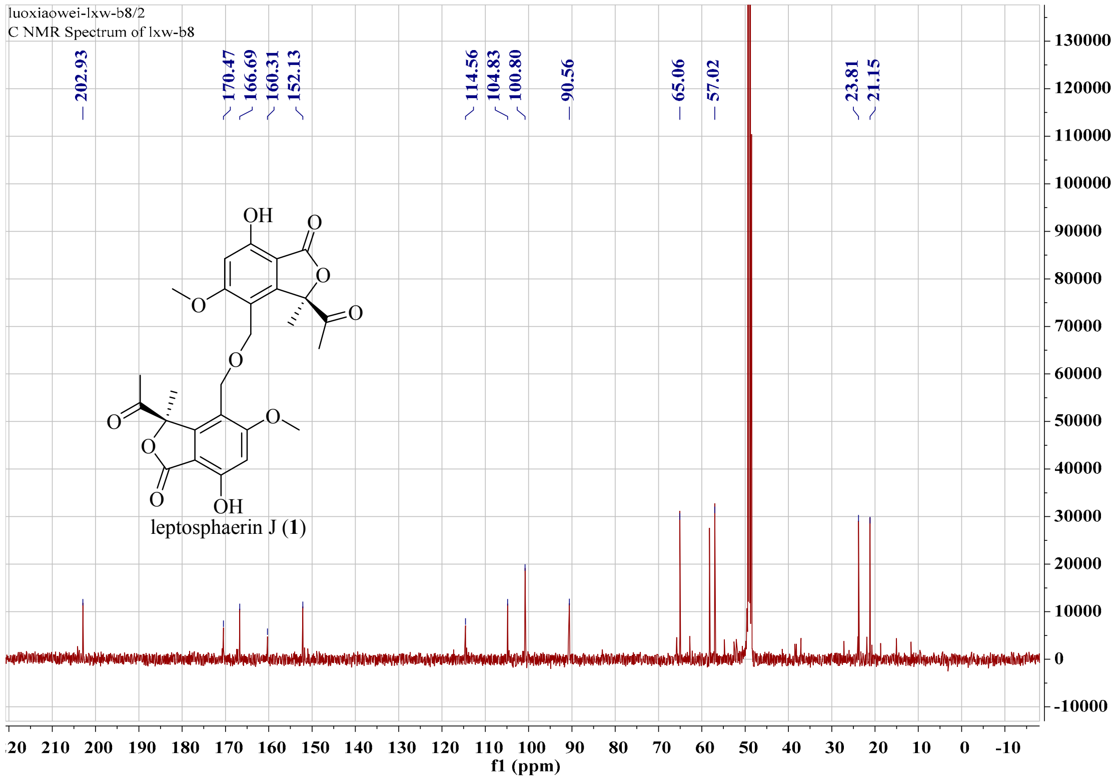

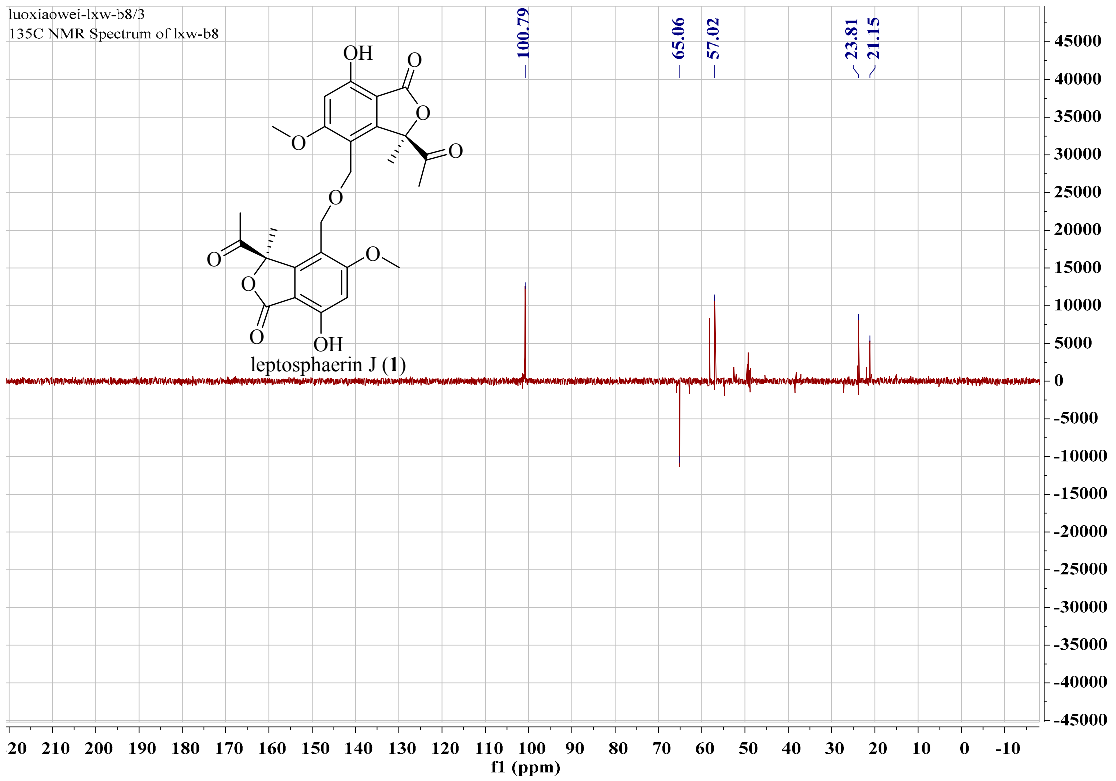

Figure S2. ^{13}C NMR and DEPT spectrum of leptosphaerin J (**1**) (MeOD)

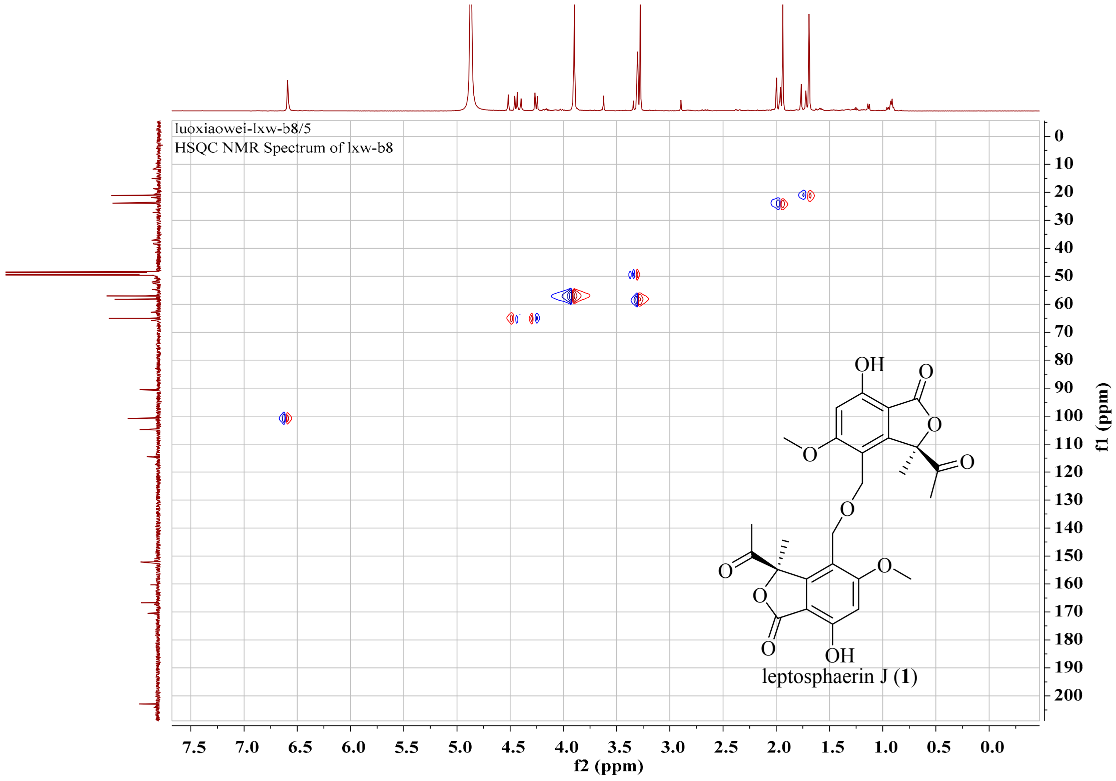

Figure S3. HSQC spectrum of leptosphaerin J (**1**) (MeOD)

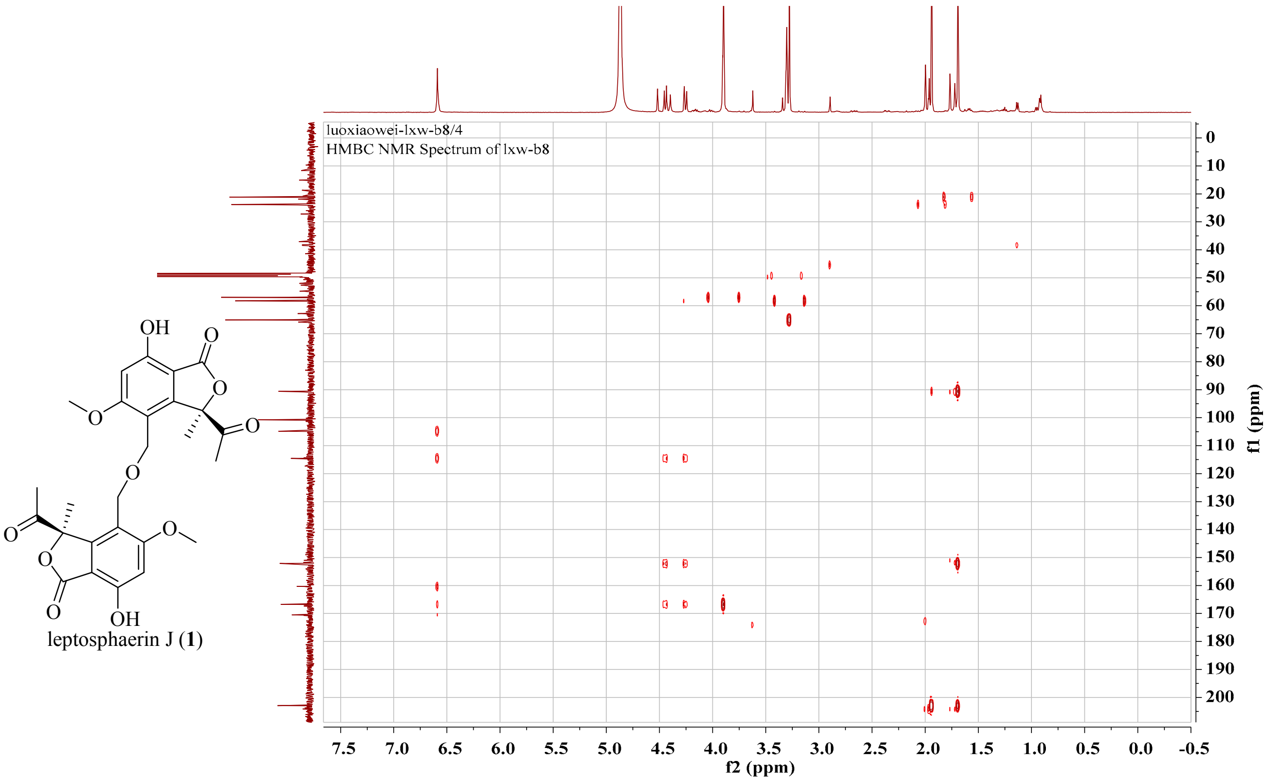

Figure S4. HMBC spectrum of leptosphaerin J (**1**) (MeOD)

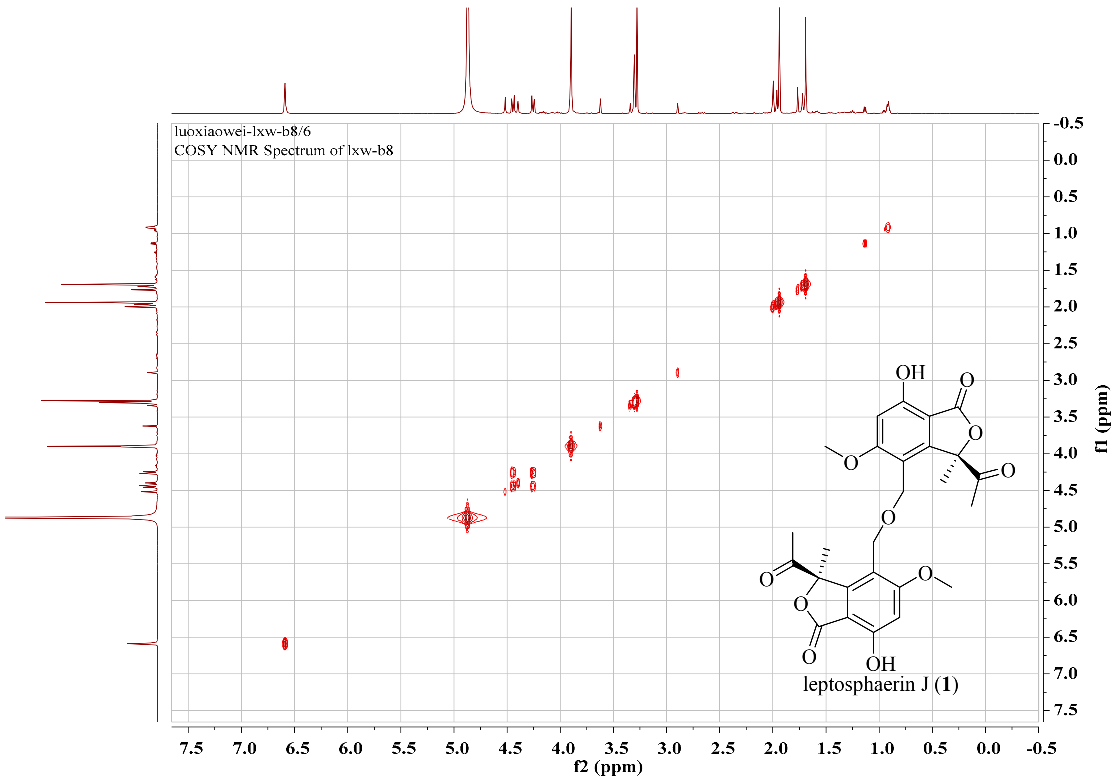

Figure S5. ^{1}H-^{1}H COSY spectrum of leptosphaerin J (**1**) (MeOD)

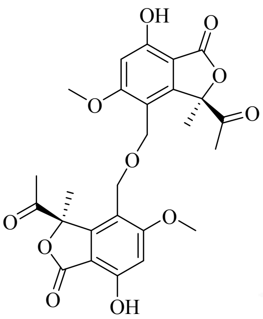

leptosphaerin J (**1**)

Mass Spectrum SmartFormula Report

Analysis Info

Analysis Name	D:\Data\MS\data\201607\luoxiaowei_lxw-b8_pos_21_01_2096.d	Acquisition Date	7/7/2016 9:56:38 AM
Method	LC_Direct Infusion_pos_100-1000mz.m	Operator	SCSIO
Sample Name	luoxiaowei_lxw-b8_pos	Instrument / Ser#	maXis 29
Comment			

Acquisition Parameter

Source Type	ESI	Ion Polarity	Positive	Set Nebulizer	0.4 Bar
Focus	Active	Set Capillary	4500 V	Set Dry Heater	180 °C
Scan Begin	100 m/z	Set End Plate Offset	-500 V	Set Dry Gas	4.0 l/min
Scan End	2000 m/z	Set Collision Cell RF	800.0 Vpp	Set Divert Valve	Waste

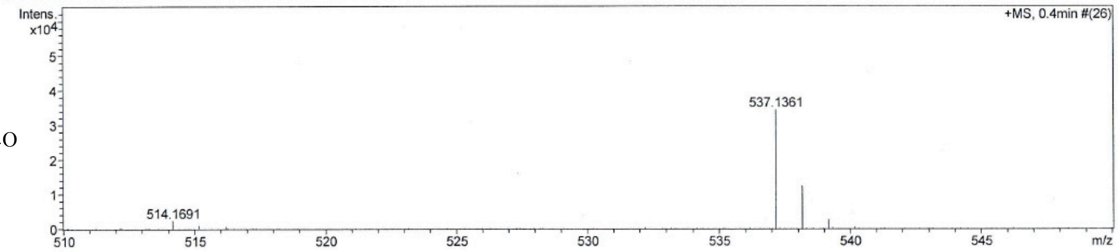

Meas. m/z	#	Formula	Score	m/z	err [ppm]	err [mDa]	mSigma	rdb	e⁻ Conf	N-Rule
537.1361	1	C 26 H 26 Na O 11	71.54	537.1367	1.2	0.7	38.5	13.5	even	ok
1051.2844	1	C 52 H 52 Na O 22	88.35	1051.2842	-0.1	-0.1	34.9	26.5	even	ok

Figure S6. Positive HRESIMS spectrum of leptosphaerin J (**1**)

Mass Spectrum SmartFormula Report

Analysis Info

Analysis Name	D:\Data\MS\data\201607\luoxiaowei_lxw-b8_neg_21_01_2101.d	Acquisition Date	7/7/2016 11:56:04 AM
Method	LC_Direct Infusion_neg_100-1000mz.m	Operator	SCSIO
Sample Name	luoxiaowei_lxw-b8_neg	Instrument / Ser#	maXis 29
Comment			

Acquisition Parameter

Source Type	ESI	Ion Polarity	Negative	Set Nebulizer	0.4 Bar
Focus	Active	Set Capillary	4000 V	Set Dry Heater	180 °C
Scan Begin	100 m/z	Set End Plate Offset	-500 V	Set Dry Gas	4.0 l/min
Scan End	2000 m/z	Set Collision Cell RF	550.0 Vpp	Set Divert Valve	Waste

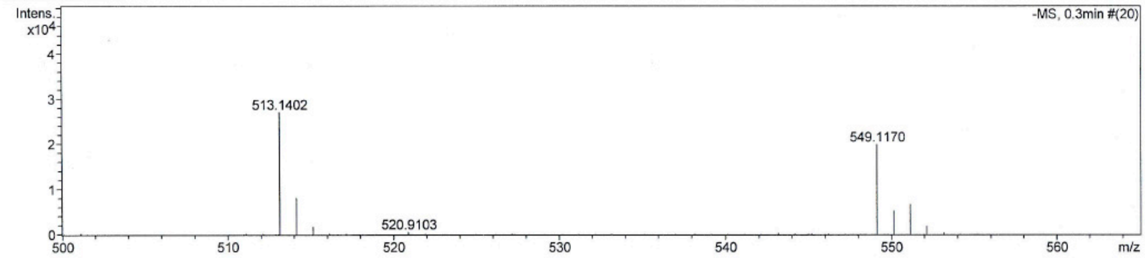

Meas. m/z	#	Formula	Score	m/z	err [ppm]	err [mDa]	mSigma	rdb	e⁻ Conf	N-Rule
513.1402	1	C 26 H 25 O 11	100.00	513.1402	0.0	0.0	8.0	14.5	even	ok
549.1170	1	C 26 H 26 Cl O 11	100.00	549.1169	-0.1	-0.1	22.0	13.5	even	ok

Figure S7. Negative HRESIMS spectrum of leptosphaerin J (**1**)

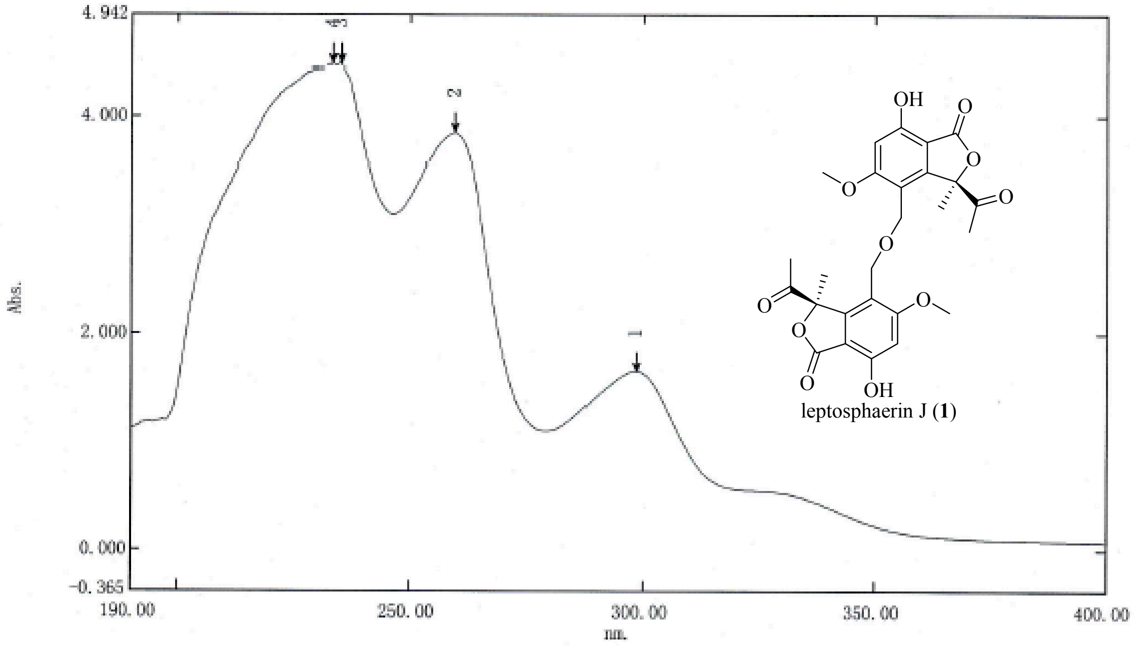

[测定属性]
波长范围 (nm.): 190.00 到 400.00
扫描速度: 中速
采样间隔: 0.2
自动采样间隔: 启用
扫描模式: 单个

[仪器属性]
仪器类型: UV-2600 系列
测定方式: 吸收值
狭缝宽: 2.0
积分时间: 0.1 秒.
光源转换波长: 323.0 nm
检测器单元: 直接
S/R 转换: 标准
阶梯校正: OFF

No.	波长(nm)	吸收值	描述
1	331.40	0.516	
2	298.40	1.645	
3	259.40	3.848	
4	234.20	4.469	
5			

Figure S8. UV spectrum of leptosphaerin J (**1**)

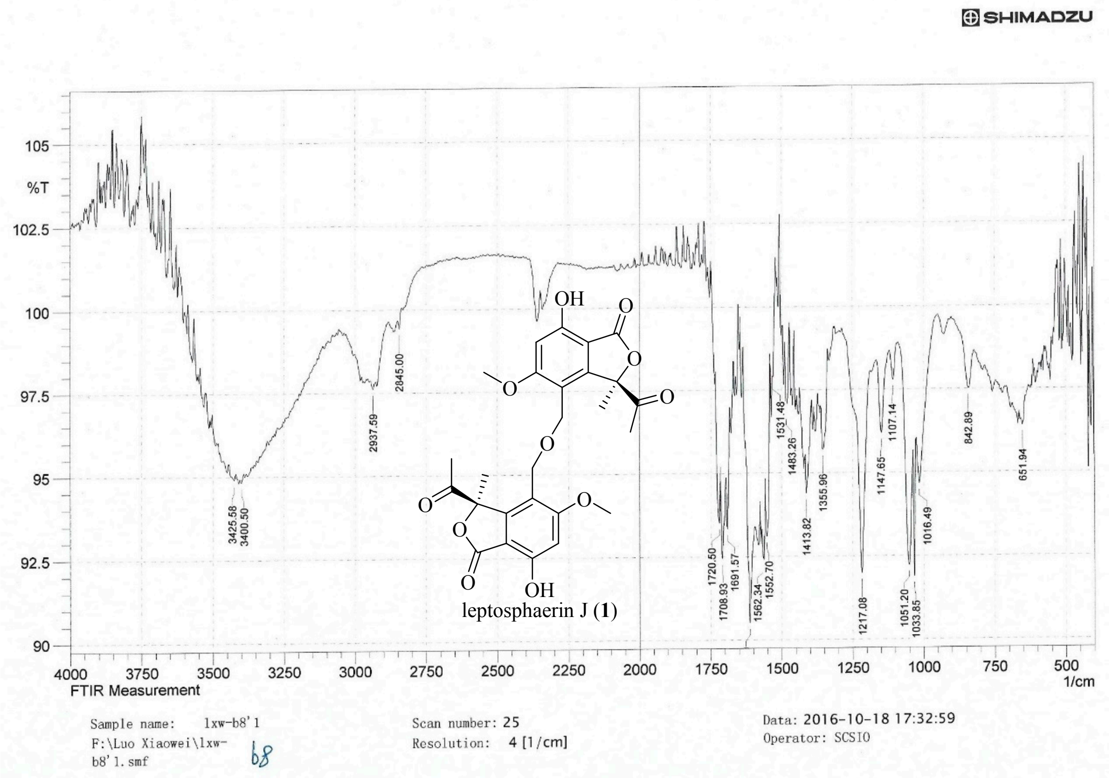

Figure S9. IR spectrum of leptosphaerin J (**1**)

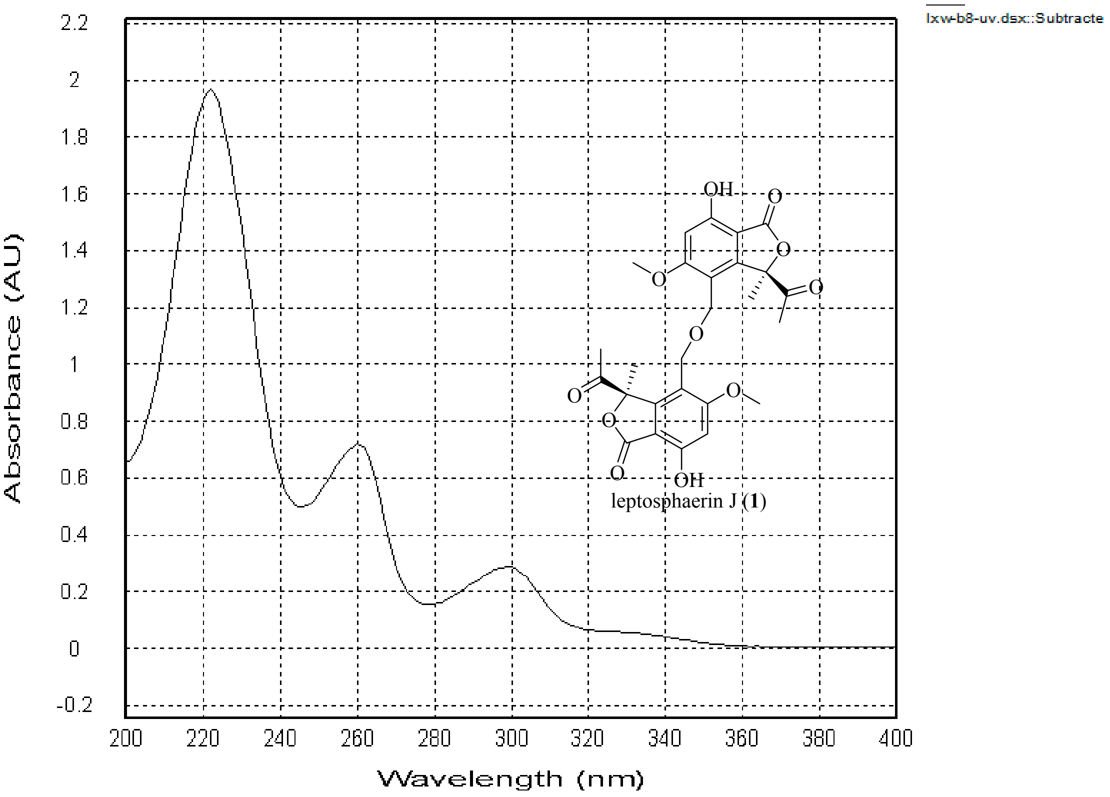

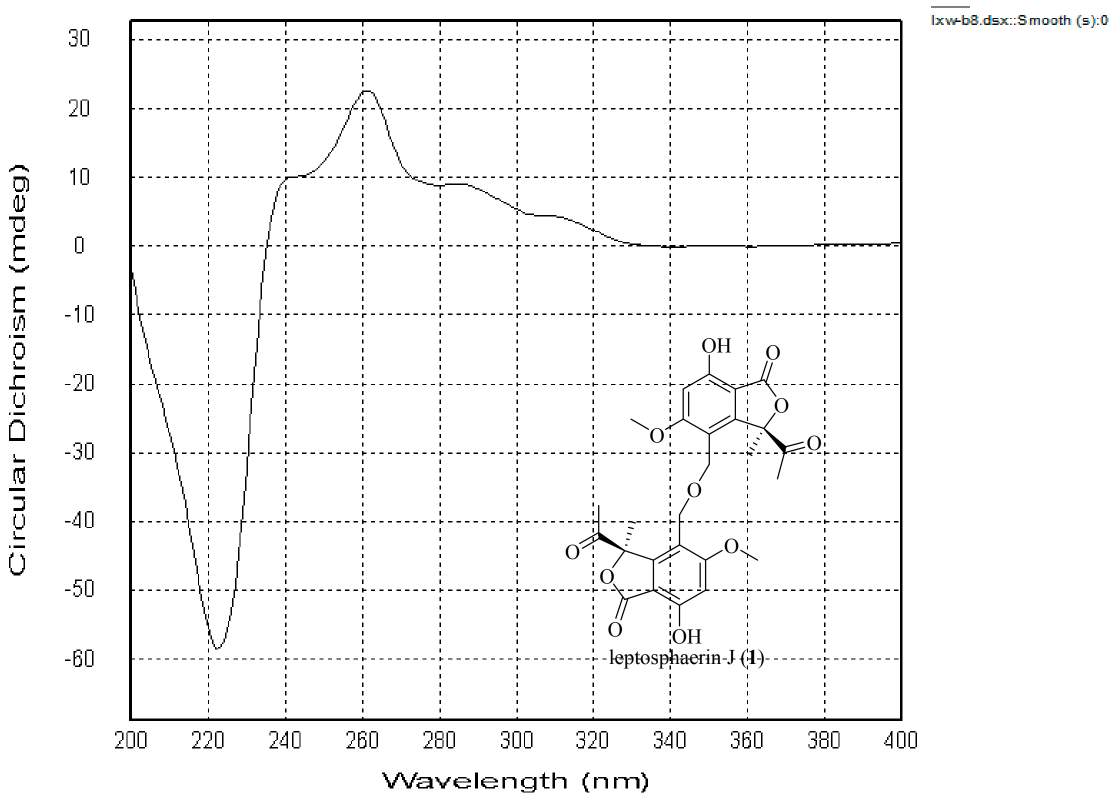

Figure S10. UV and CD spectrum of leptosphaerin J (**1**)

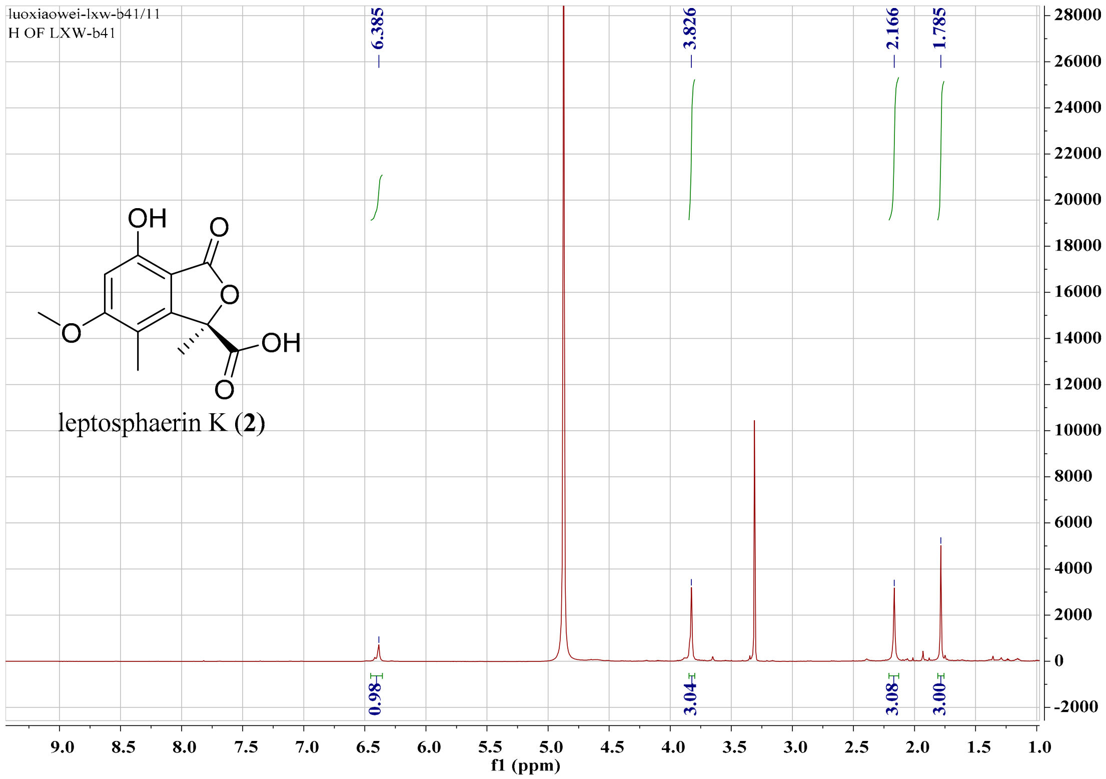

Figure S11. ^{1}H NMR spectrum of leptosphaerin K (**2**) (MeOD)

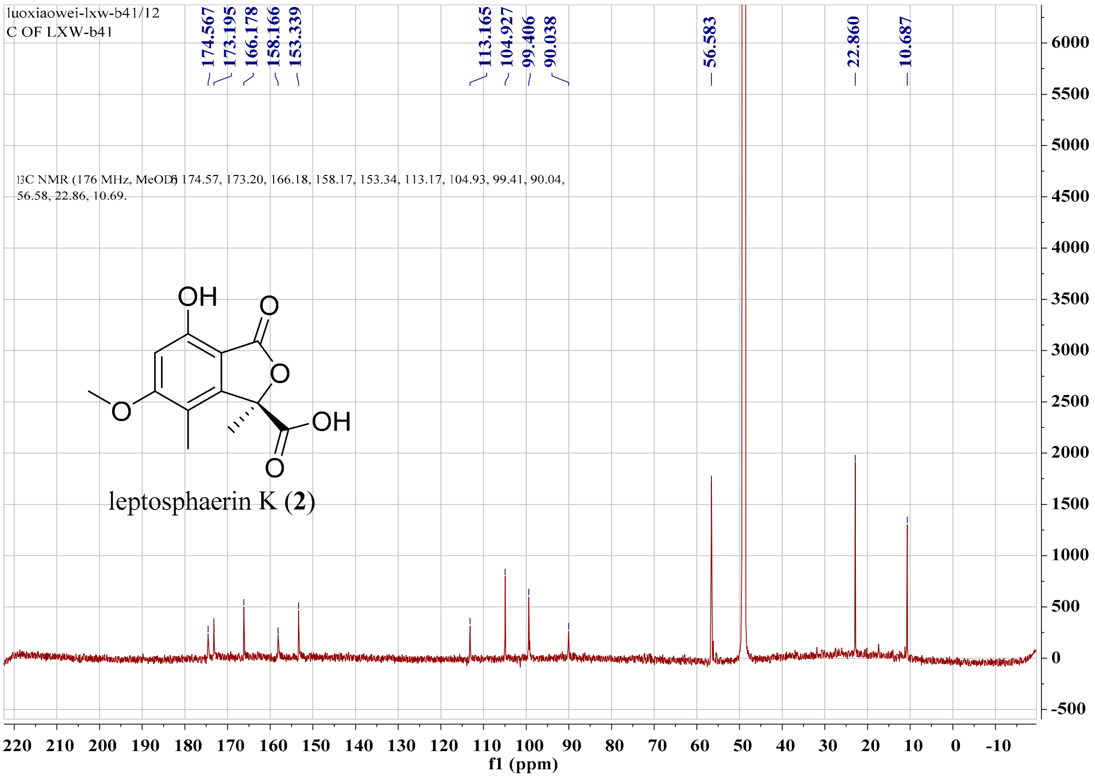

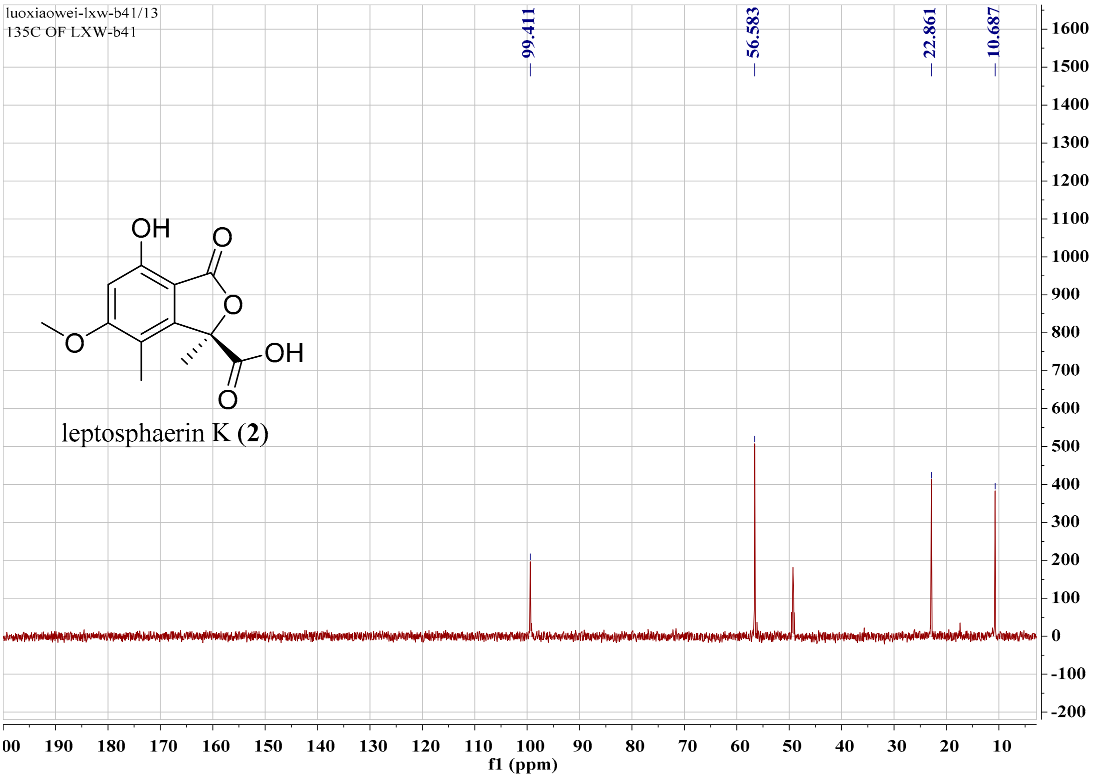

Figure S12. ^{13}C NMR and DEPT spectrum of leptosphaerin K (**2**) (MeOD)

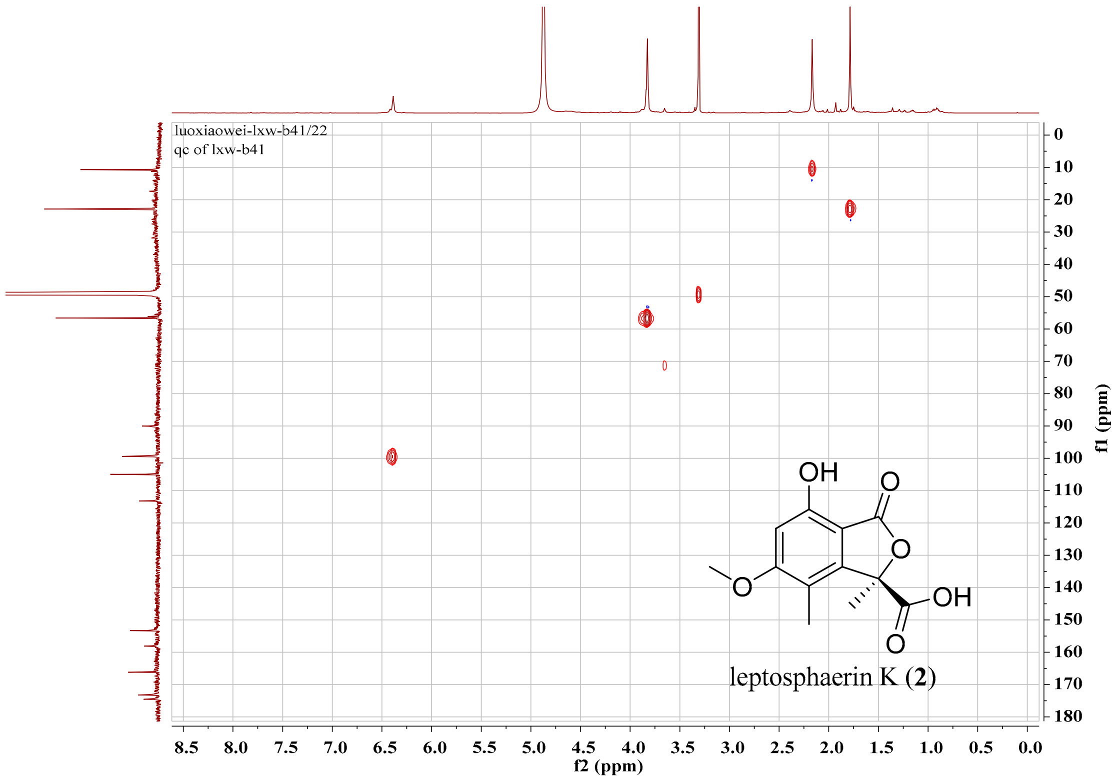

Figure S13. HSQC spectrum of leptosphaerin K (**2**) (MeOD)

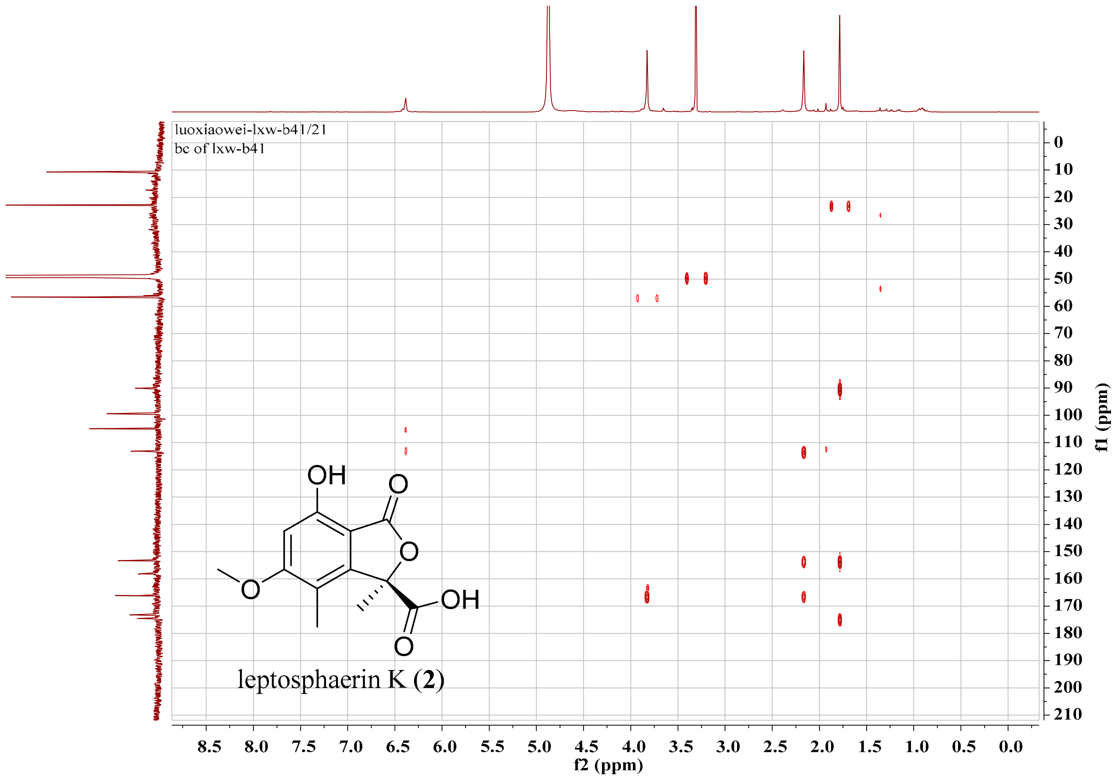

Figure S14. HMBC spectrum of leptosphaerin K (**2**) (MeOD)

Mass Spectrum SmartFormula Report

Analysis Info

Analysis Name	D:\Data\MS\data\201607\luoxiaowei_lxw-b41_neg_21_01_2107.d	Acquisition Date	7/7/2016 4:34:13 PM
Method	LC_Direct Infusion_neg_100-1000mz.m	Operator	SCSIO
Sample Name	luoxiaowei_lxw-b41_neg	Instrument / Ser#	maXis 29
Comment			

Acquisition Parameter

Source Type	ESI	Ion Polarity	Negative	Set Nebulizer	0.4 Bar
Focus	Active	Set Capillary	4000 V	Set Dry Heater	180 °C
Scan Begin	100 m/z	Set End Plate Offset	-500 V	Set Dry Gas	4.0 l/min
Scan End	2000 m/z	Set Collision Cell RF	550.0 Vpp	Set Divert Valve	Waste

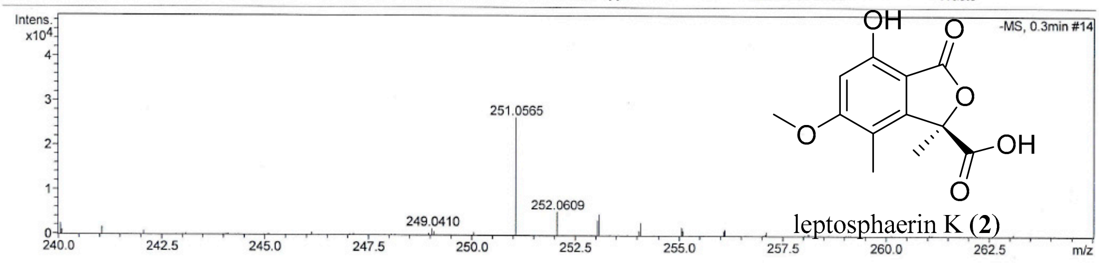

Meas. m/z	#	Formula	Score	m/z	err [ppm]	err [mDa]	mSigma	rdb	e⁻ Conf	N-Rule
251.0565	1	C 12 H 11 O 6	89.15	251.0561	-1.6	-0.4	40.7	7.5	even	ok
503.1204	1	C 24 H 23 O 12	51.55	503.1195	-1.8	-0.9	103.2	13.5	even	ok

Figure S15. HRESIMS spectrum of leptosphaerin K (**2**)

光谱峰值检测报告

2016-10-17 15:38:46

数据集: lxw-b41 - RawData

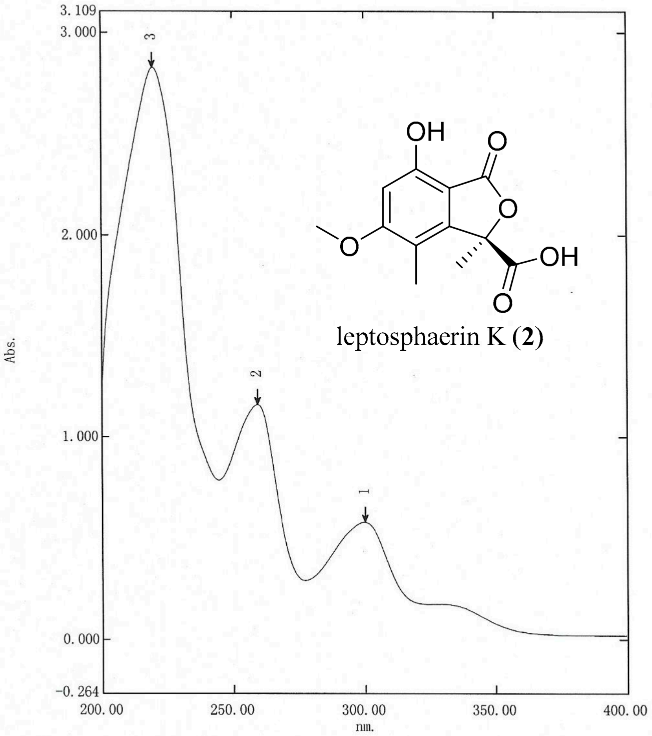

[测定属性]
波长范围 (nm.): 200.00 到 400.00
扫描速度: 中速
采样间隔: 0.2
自动采样间隔: 启用
扫描模式: 单个

[仪器属性]
仪器类型: UV-2600 系列
测定方式: 吸收值
狭缝宽: 2.0
积分时间: 0.1 秒.
光源转换波长: 323.0 nm
检测器单元: 直接
S/R 转换: 标准
阶梯校正: OFF

[附件属性]
附件: 无

[数据处理参数]
阈值: 0.0100000
点: 4
内插: 停用
平均: 停用

[样品准备属性]
重量:
体积:
稀释:
光程长:
附加信息: 45 μg/ml
252

No.	P/V	波长(nm)	吸收值	描述 logε
1	↑	299.80	0.578	3.510
2	↑	259.20	1.161	3.813
3	↑	219.80	2.828	4.200

页 1 / 1

Figure S16. UV spectrum of leptosphaerin K (**2**)

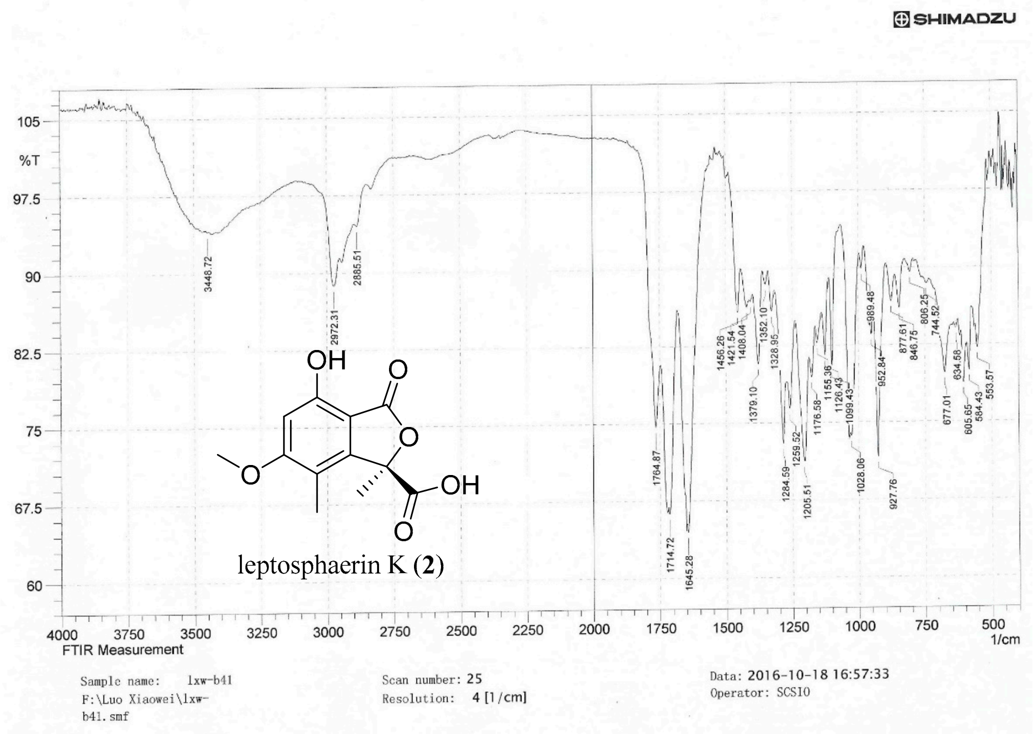

Figure S17. IR spectrum of leptosphaerin K (**2**)

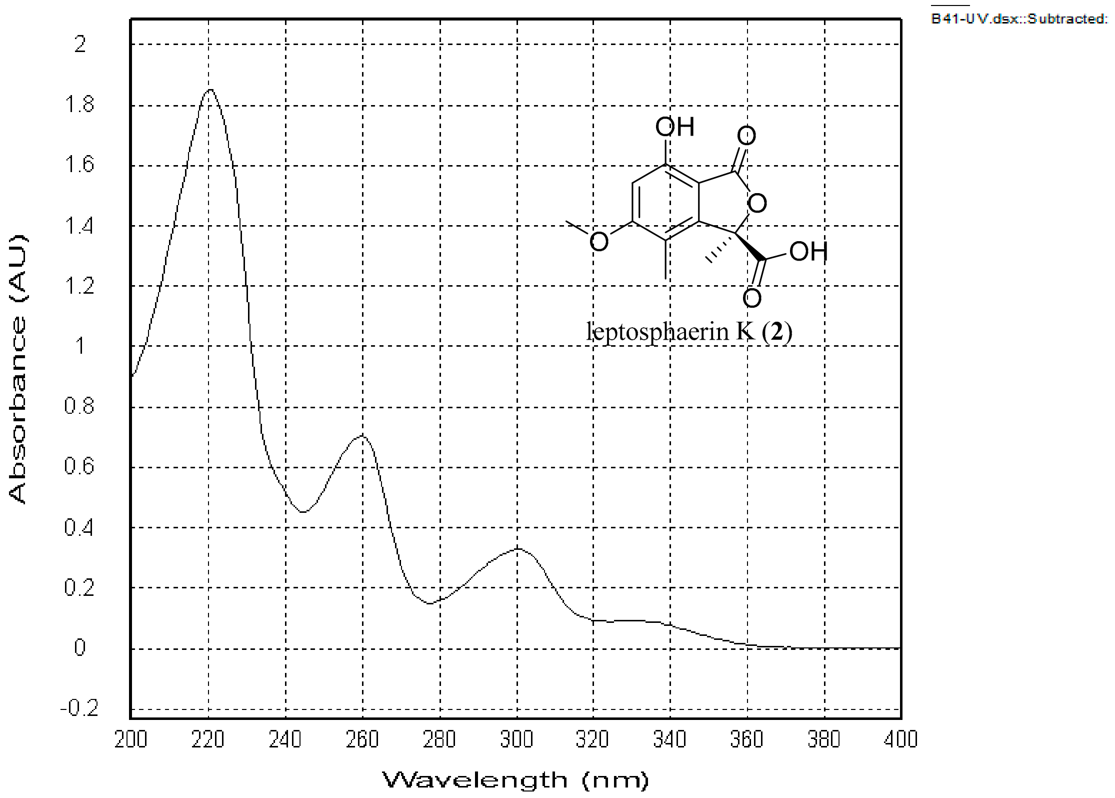

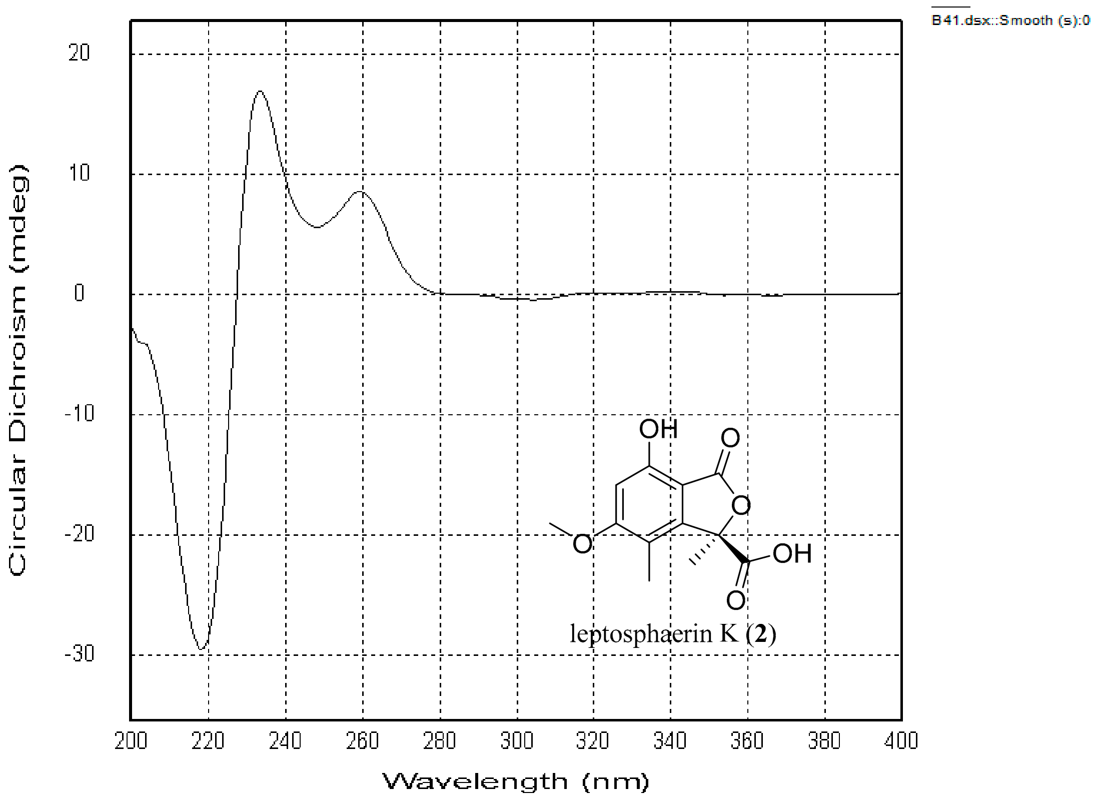

Figure S18. UV and CD spectrum of leptosphaerin K (**2**)

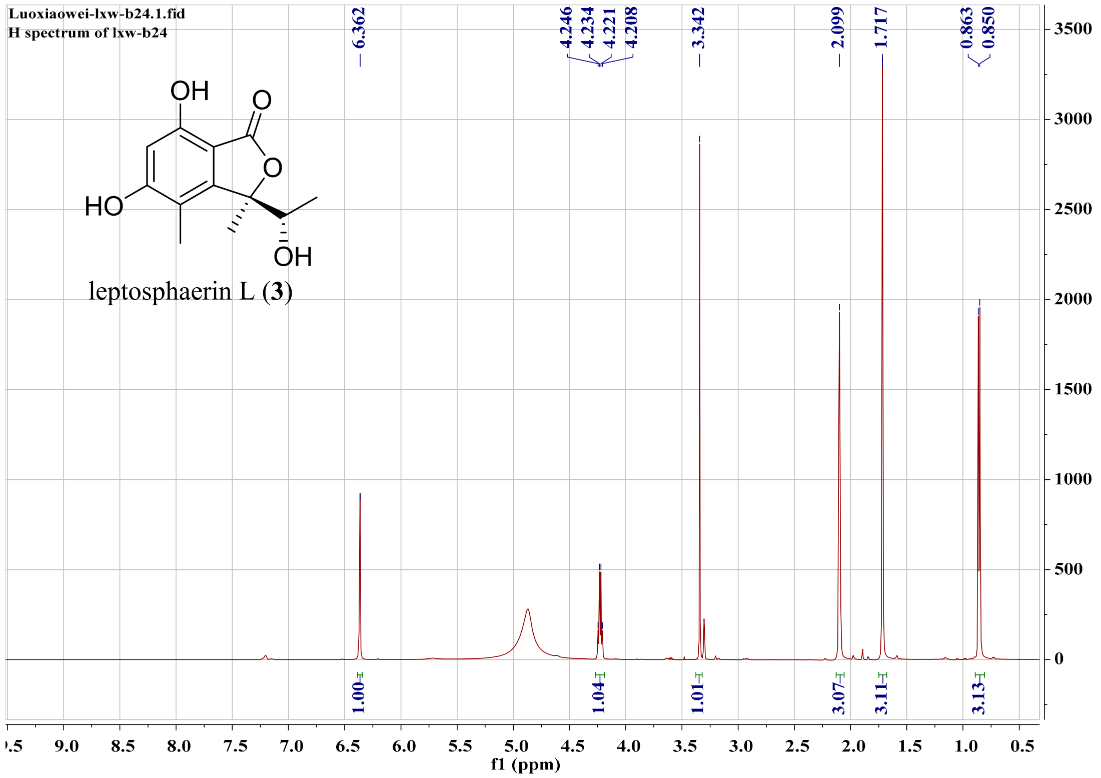

Figure S19. ^{1}H NMR spectrum of leptosphaerin L (**3**) (MeOD)

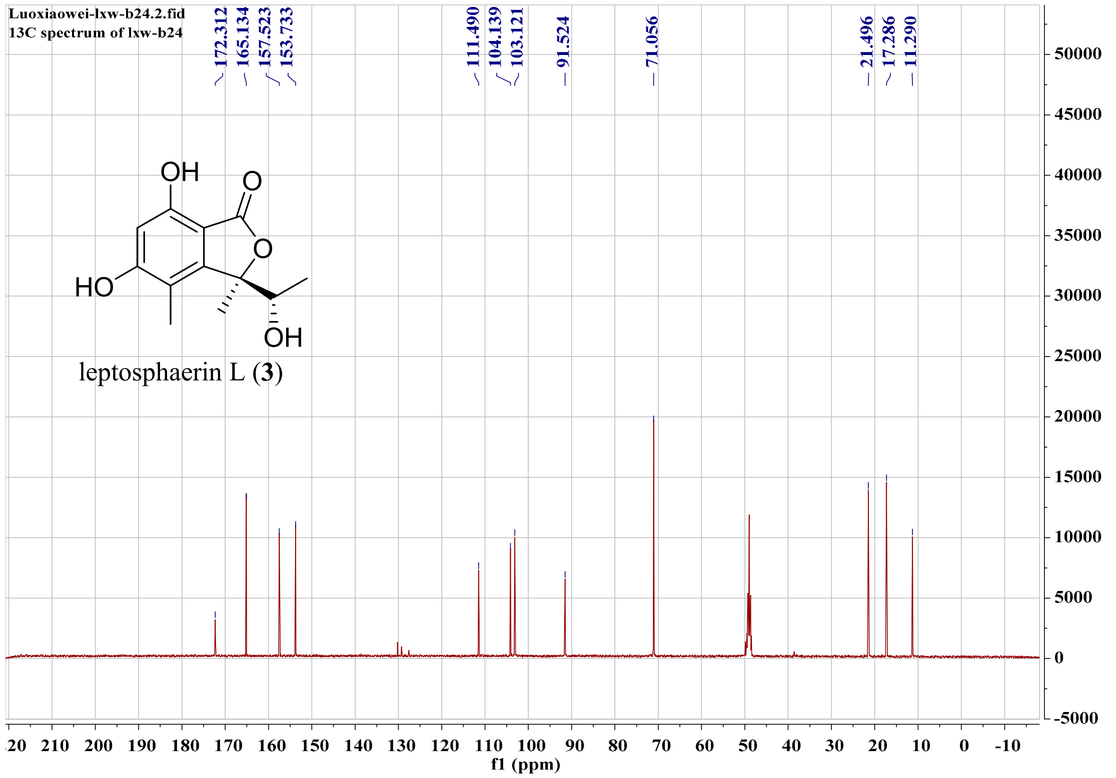

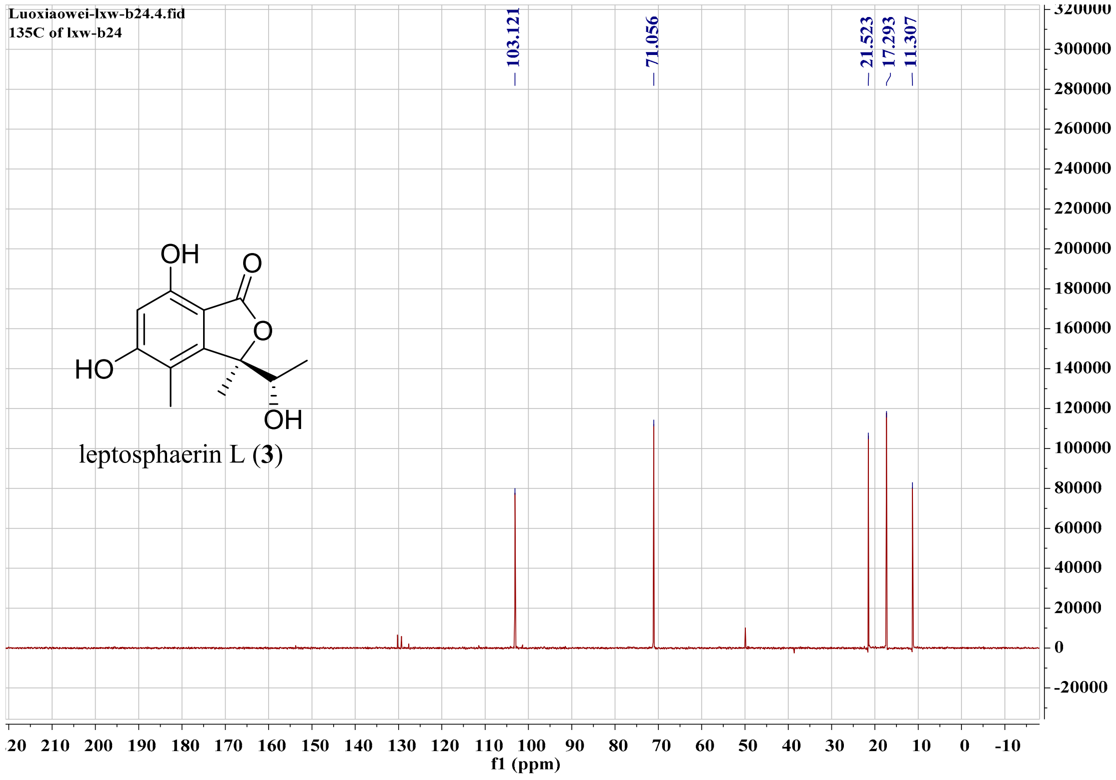

Figure S20. ^{13}C NMR and DEPT spectrum of leptosphaerin L (**3**) (MeOD)

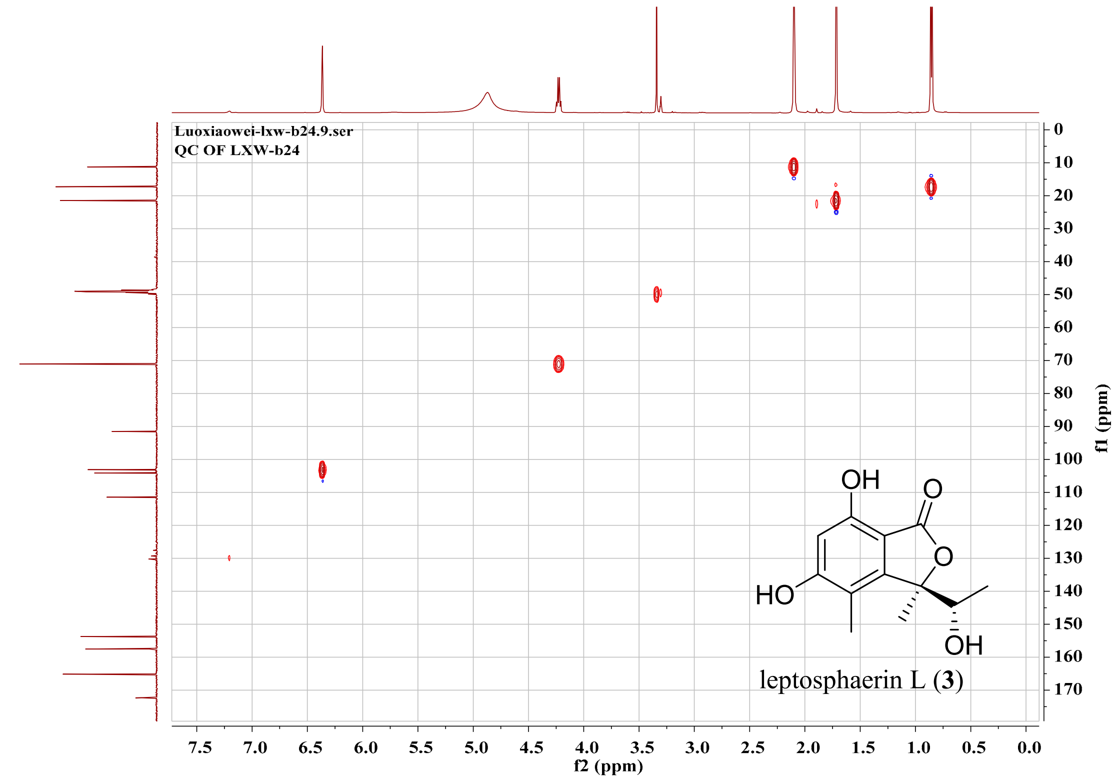

Figure S21. HSQC spectrum of leptosphaerin L (**3**) (MeOD)

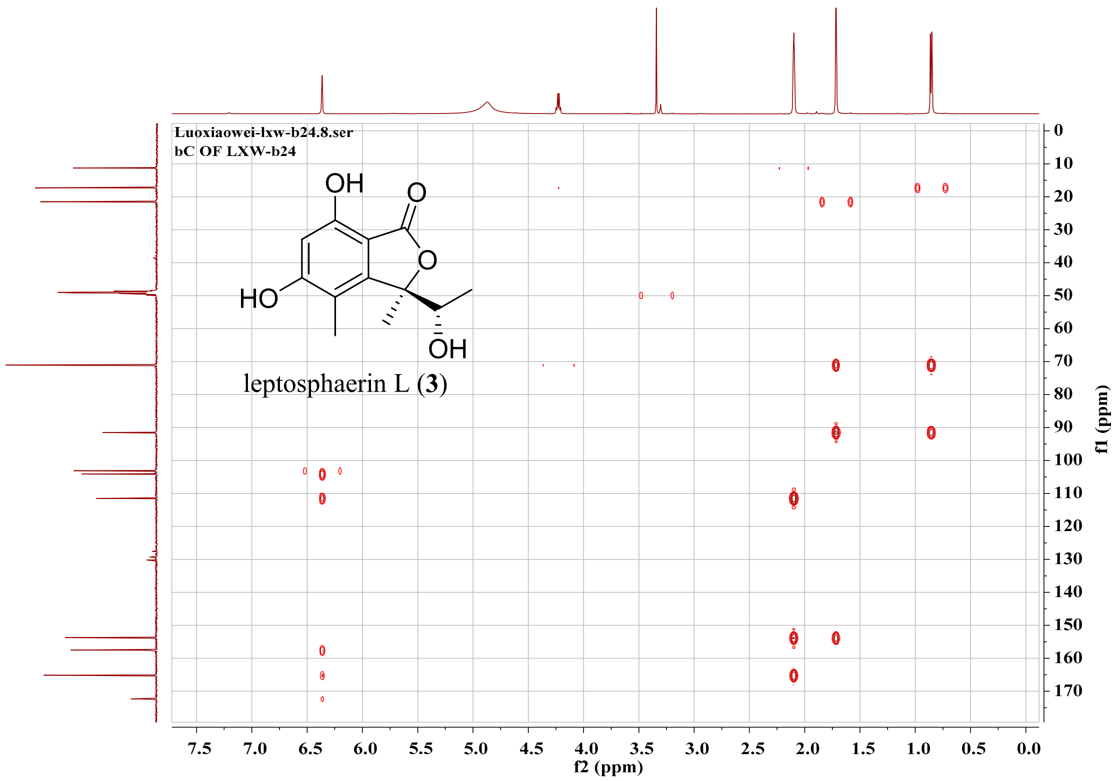

Figure S22. HMBC spectrum of leptosphaerin L (**3**) (MeOD)

Mass Spectrum SmartFormula Report

Analysis Info

Analysis Name	D:\Data\MS\data\201609\luoxiaowei_lxw-b24_neg_32_01_2378.d	Acquisition Date	9/13/2016 9:31:09 AM
Method	LC_Direct Infusion_neg_100-1000mz.m	Operator	SCSIO
Sample Name	luoxiaowei_lxw-b24_neg	Instrument / Ser#	maXis 29
Comment			

Acquisition Parameter

Source Type	ESI	Ion Polarity	Negative	Set Nebulizer	0.4 Bar
Focus	Active	Set Capillary	4000 V	Set Dry Heater	180 °C
Scan Begin	100 m/z	Set End Plate Offset	-500 V	Set Dry Gas	4.0 l/min
Scan End	2000 m/z	Set Collision Cell RF	550.0 Vpp	Set Divert Valve	Waste

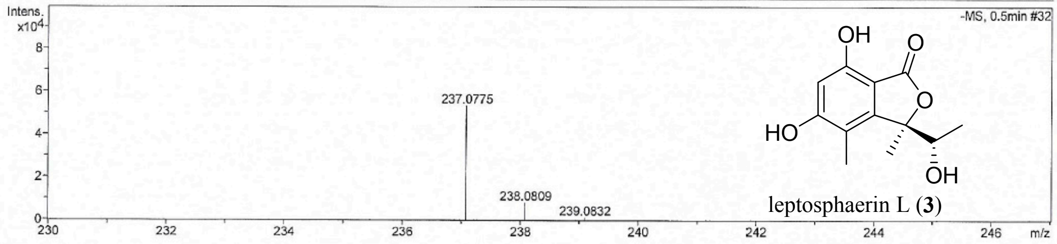

Meas. m/z	#	Formula	Score	m/z	err [ppm]	err [mDa]	mSigma	rdb	e⁻ Conf	N-Rule
237.0775	1	C 12 H 13 O 5	90.27	237.0768	-2.7	-0.6	12.5	6.5	even	ok
475.1605	1	C 24 H 27 O 10	100.00	475.1610	1.0	0.5	3.5	11.5	even	ok

Figure S23. HRESIMS spectrum of leptosphaerin L (**3**)

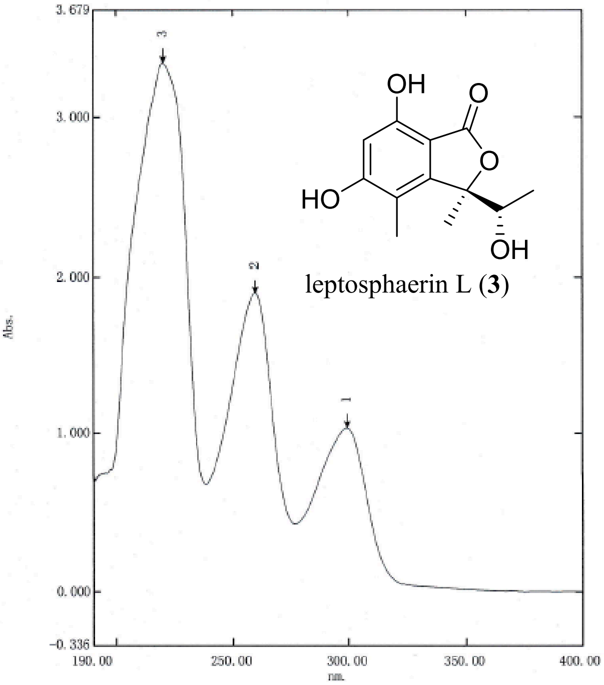

[测定属性]
波长范围（nm.）：190.00 到 400.00
扫描速度：中速
采样间隔：0.2
自动采样间隔：启用
扫描模式：单个

[仪器属性]
仪器类型：UV-2600 系列
测定方式：吸收值
狭缝宽：2.0
积分时间：0.1 秒.
光源转换波长：323.0 nm
检测器单元：直接
S/R 转换：标准
阶梯校正：OFF

[附件属性]
附件：无

[数据处理参数]
阈值：0.0100000
点：4
内插：停用
平均：停用

[样品准备属性]
重量：
体积：
稀释：
光程长：
附加信息：

No.	P/V	波长(nm)	吸收值	描述 log ε
峰 1	↑	299.20	1.034	3.76
2	↑	259.80	1.890	4.03
3	↑	220.20	3.345	4.28
谷 4	↓	322.60	0.047	
5	↓	276.80	0.429	
6	↓	238.60	0.681	

Figure S24. UV spectrum of leptosphaerin L (**3**)

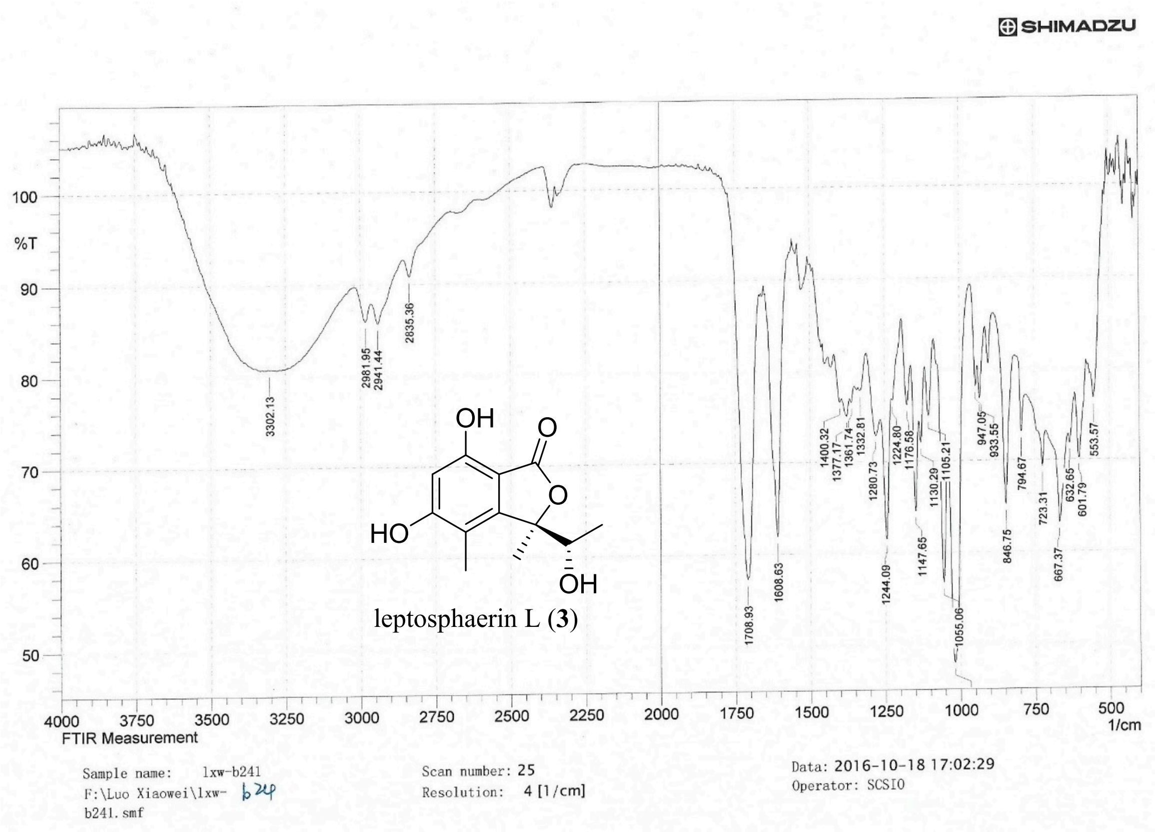

Figure S25. IR spectrum of leptosphaerin L (**3**)

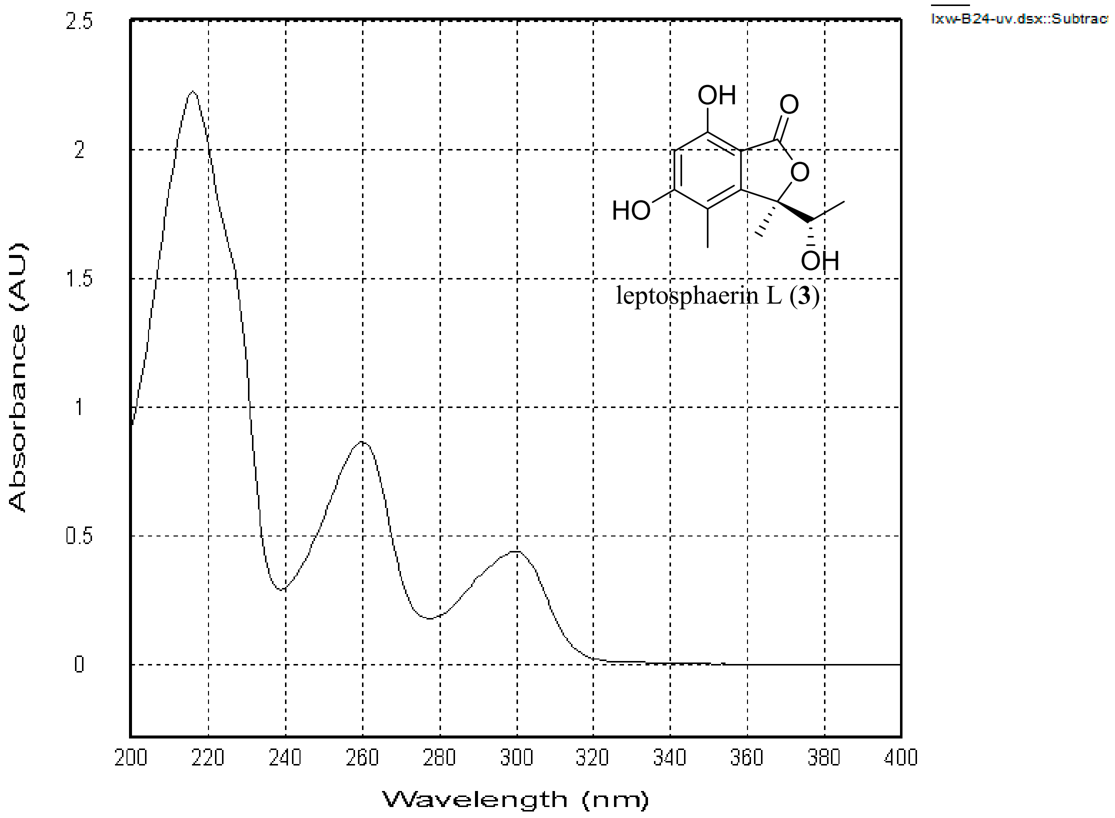

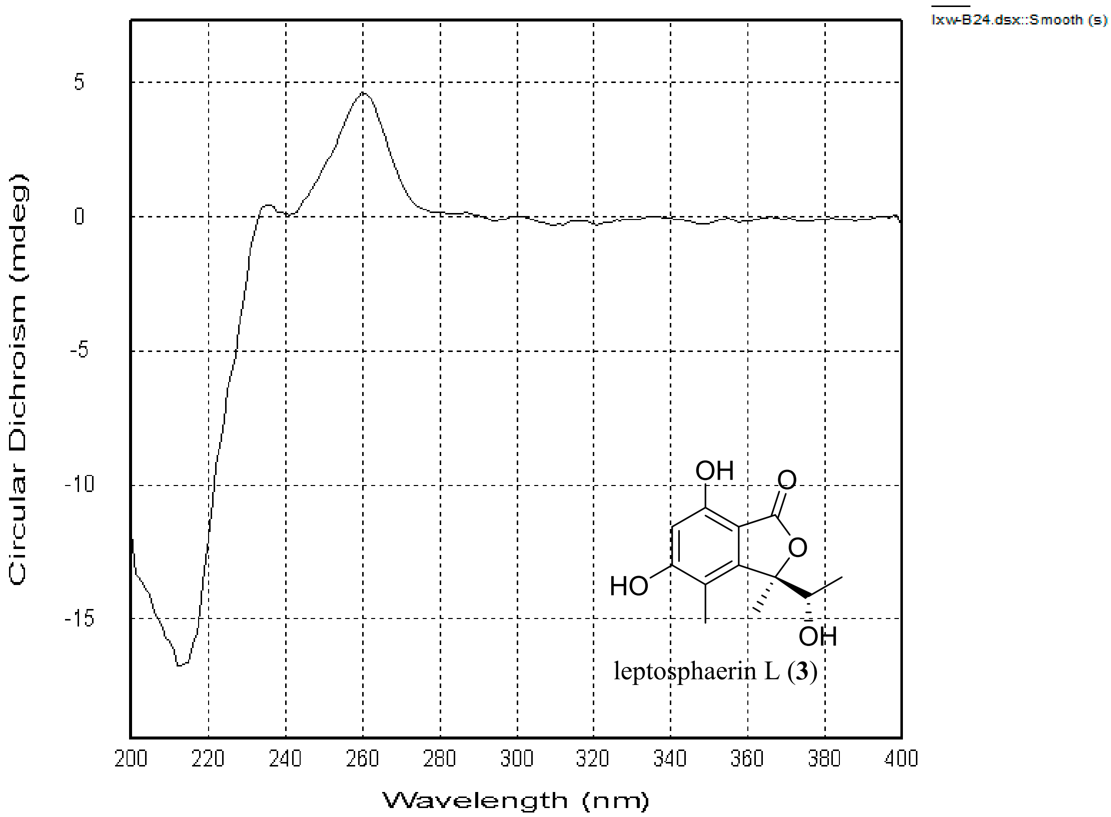

Figure S26. UV and CD spectrum of leptosphaerin L (**3**)

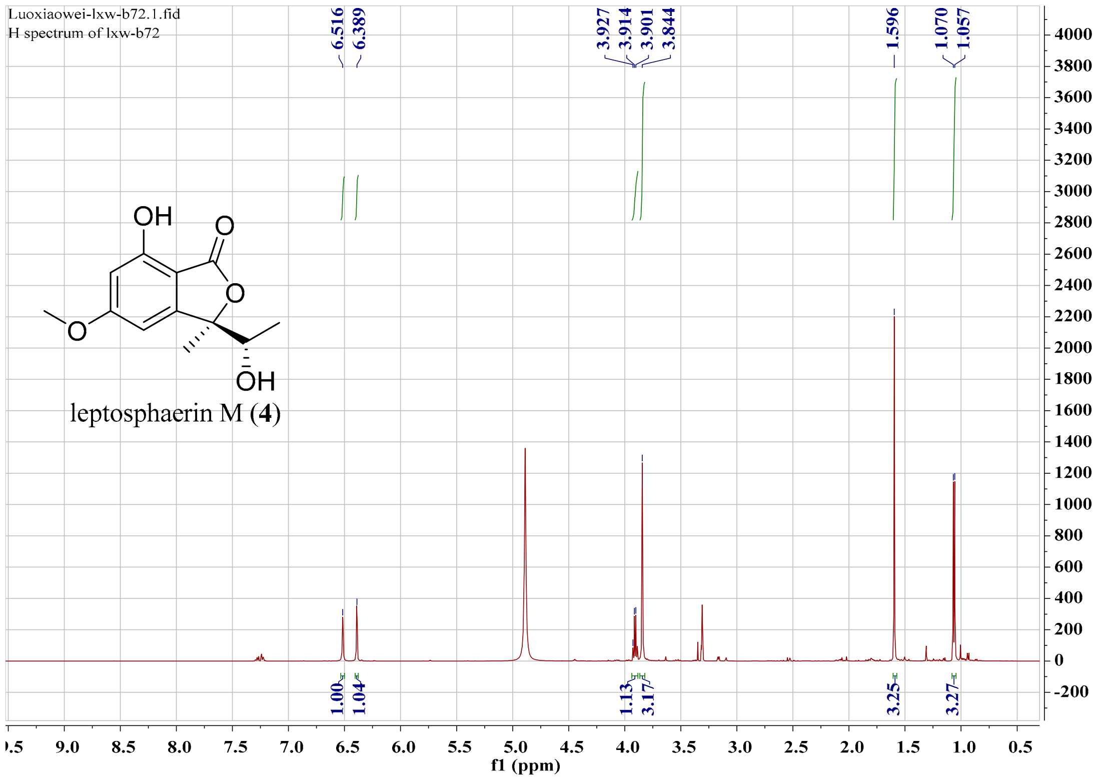

Figure S27. ^{1}H NMR spectrum of leptosphaerin M (**4**) (MeOD)

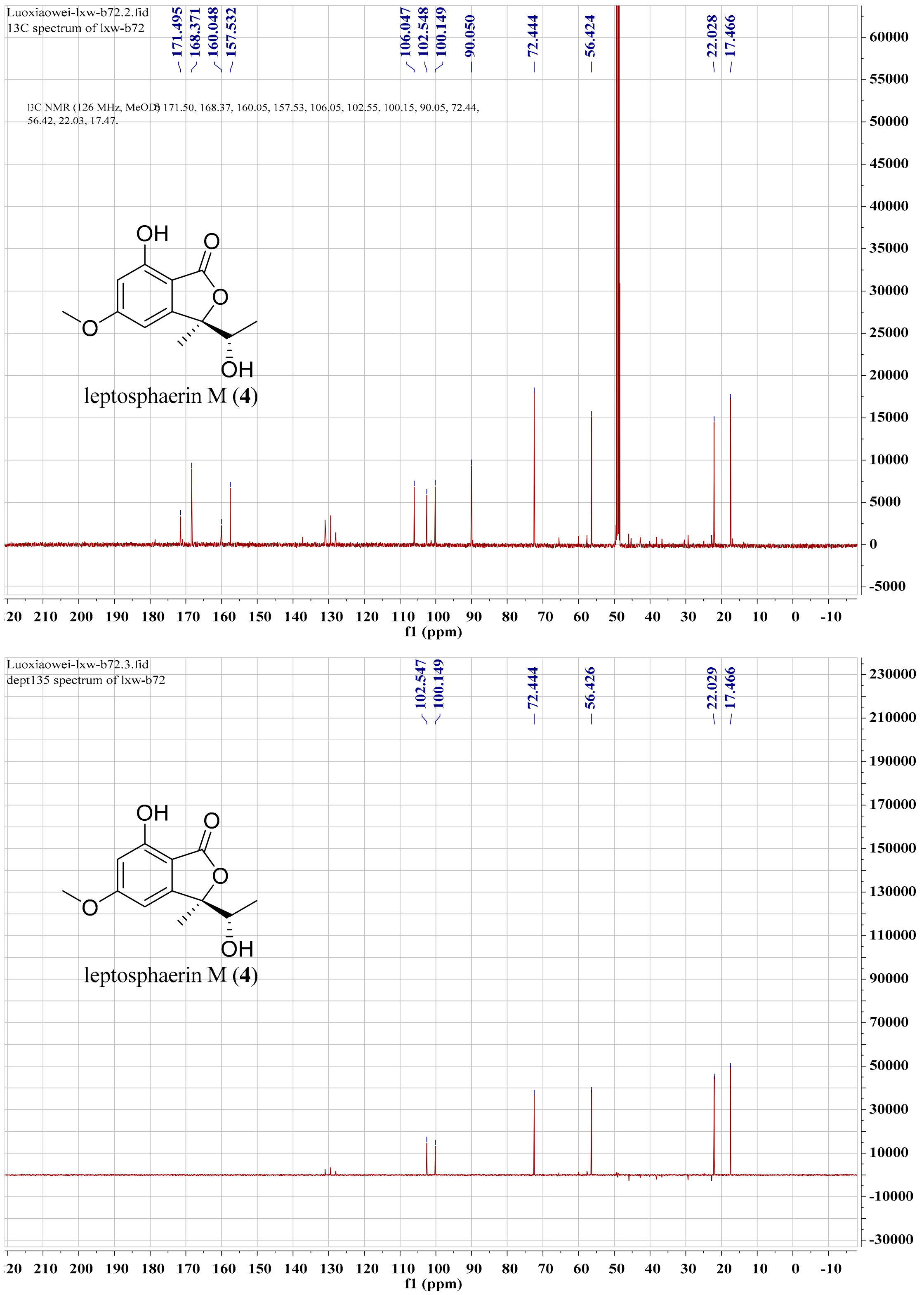

Figure S28. ^{13}C NMR and DEPT spectrum of leptosphaerin M (4) (MeOD)

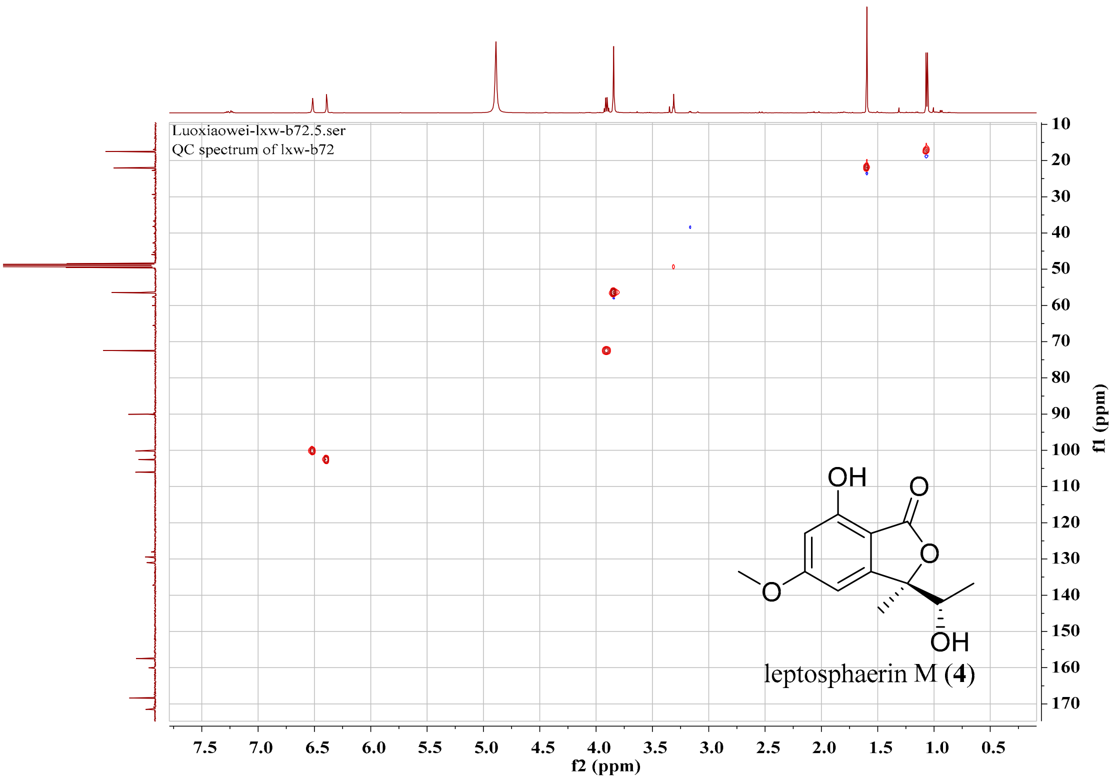

Figure S29. HSQC spectrum of leptosphaerin M (**4**) (MeOD)

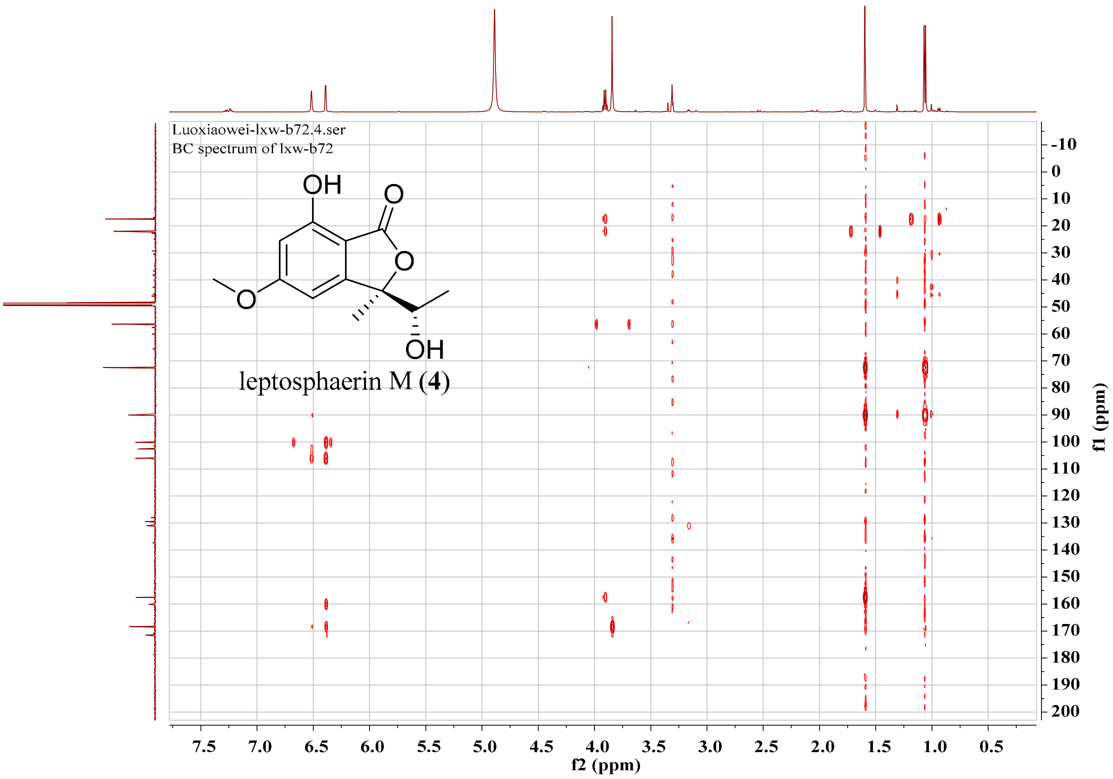

Figure S30. HMBC spectrum of leptosphaerin M (**4**) (MeOD)

Mass Spectrum SmartFormula Report

Analysis Info		Acquisition Date	10/12/2016 11:41:07 AM
Analysis Name	D:\Data\MS\data\201610\luoxiaowei_lxw-b72_neg_81_01_2457.d		
Method	LC_Direct Infusion_neg_100-1000mz.m	Operator	SCSIO
Sample Name	luoxiaowei_lxw-b72_neg	Instrument / Ser#	maXis 29
Comment			

Acquisition Parameter

Source Type	ESI	Ion Polarity	Negative	Set Nebulizer	0.4 Bar
Focus	Active	Set Capillary	4000 V	Set Dry Heater	180 °C
Scan Begin	100 m/z	Set End Plate Offset	-500 V	Set Dry Gas	4.0 l/min
Scan End	2000 m/z	Set Collision Cell RF	550.0 Vpp	Set Divert Valve	Waste

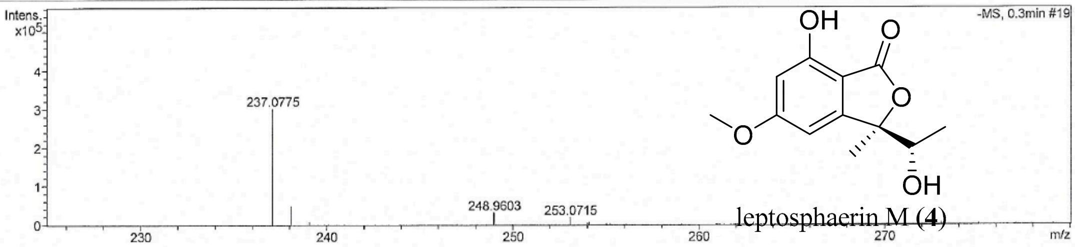

Meas. m/z	#	Formula	Score	m/z	err [ppm]	err [mDa]	mSigma	rdb	e⁻ Conf	N-Rule
237.0775	1	C 12 H 13 O 5	100.00	237.0768	-2.7	-0.6	17.0	6.5	even	ok
273.0530	1	C 12 H 14 Cl O 5	100.00	273.0535	2.0	0.5	22.9	5.5	even	ok
475.1593	1	C 24 H 27 O 10	52.50	475.1610	3.5	1.6	20.6	11.5	even	ok

Figure S31. HRESIMS spectrum of leptosphaerin M (**4**)

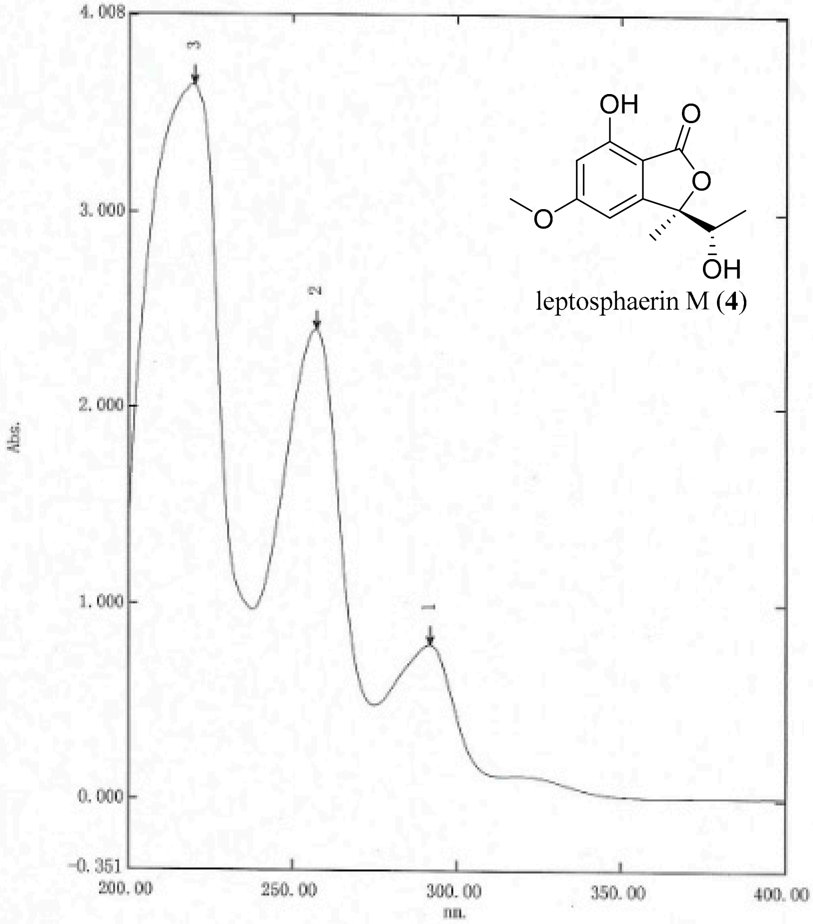

[测定属性]
波长范围 (nm.): 200.00 到 400.00
扫描速度: 中速
采样间隔: 0.2
自动采样间隔: 启用
扫描模式: 单个

[仪器属性]
仪器类型: UV-2600 系列
测定方式: 吸收值
狭缝宽: 2.0
积分时间: 0.1 秒.
光源转换波长: 323.0 nm
检测器单元: 直接
S/R 转换: 标准
阶梯校正: OFF

[附件属性]
附件: 无

[数据处理参数]
阈值: 0.0100000
点: 4
内插: 停用
平均: 停用

[样品准备属性]
重量:
体积:
稀释:
光程长:
附加信息:

50ug/ml
228

No.	P/V	波长(nm)	吸收值	描述 logε
1	↑	291.60	0.793	3.577
2	↑	257.00	2.399	4.058
3	↑	220.00	3.645	4.239

Figure S32. UV spectrum of leptosphaerin M (**4**)

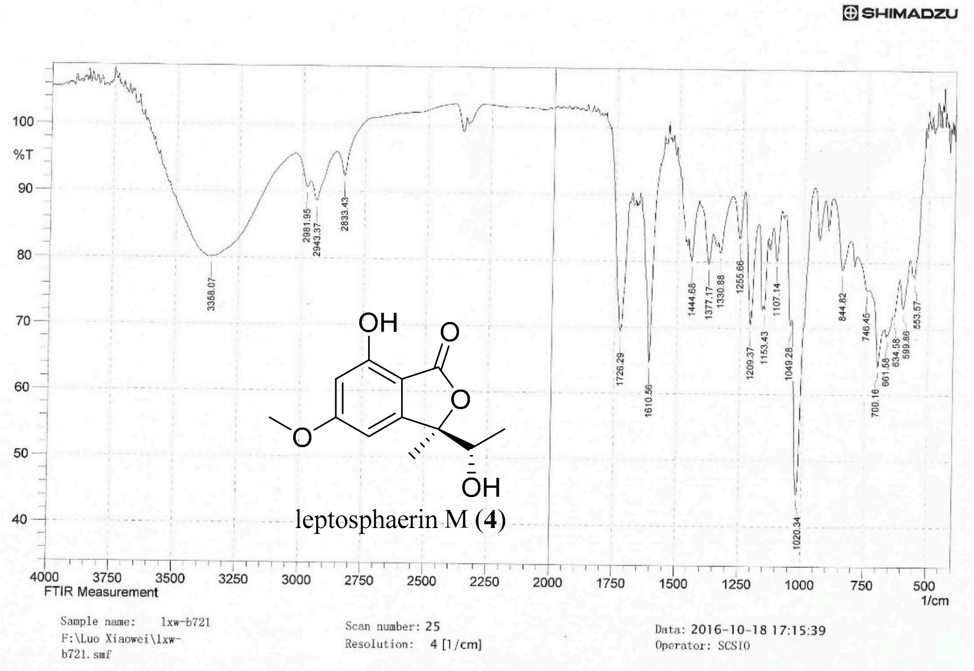

Figure S33. IR spectrum of leptosphaerin M (**4**)

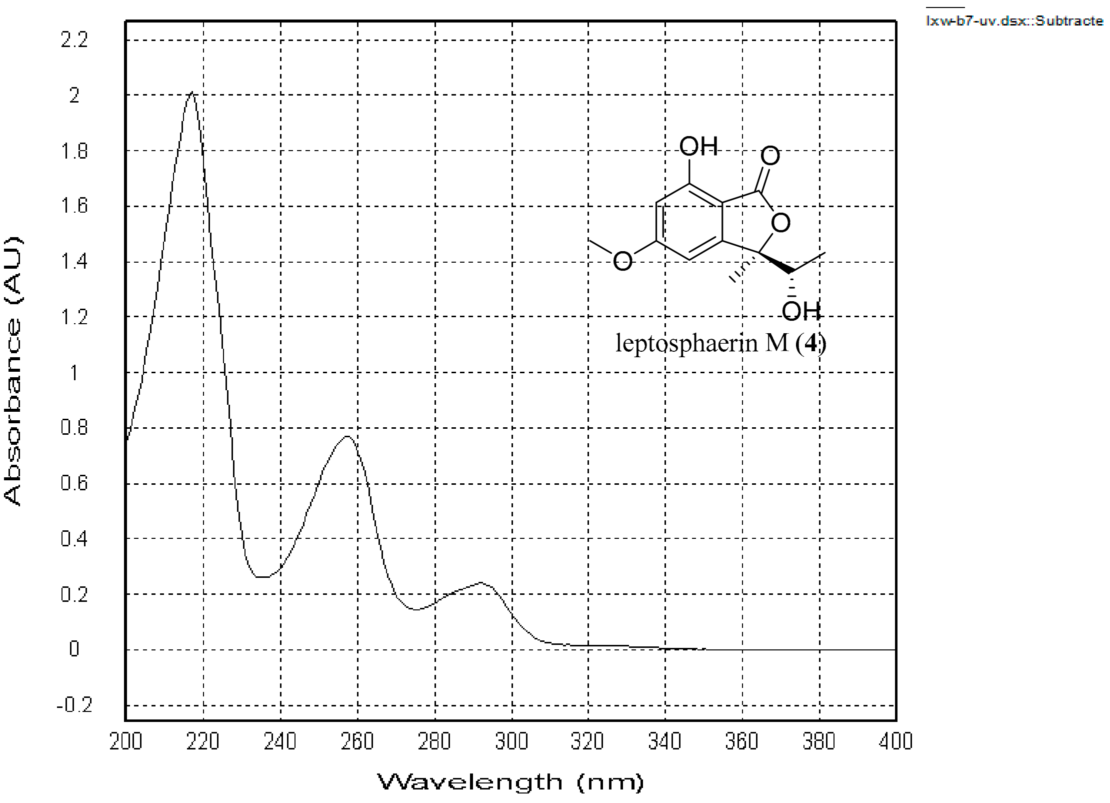

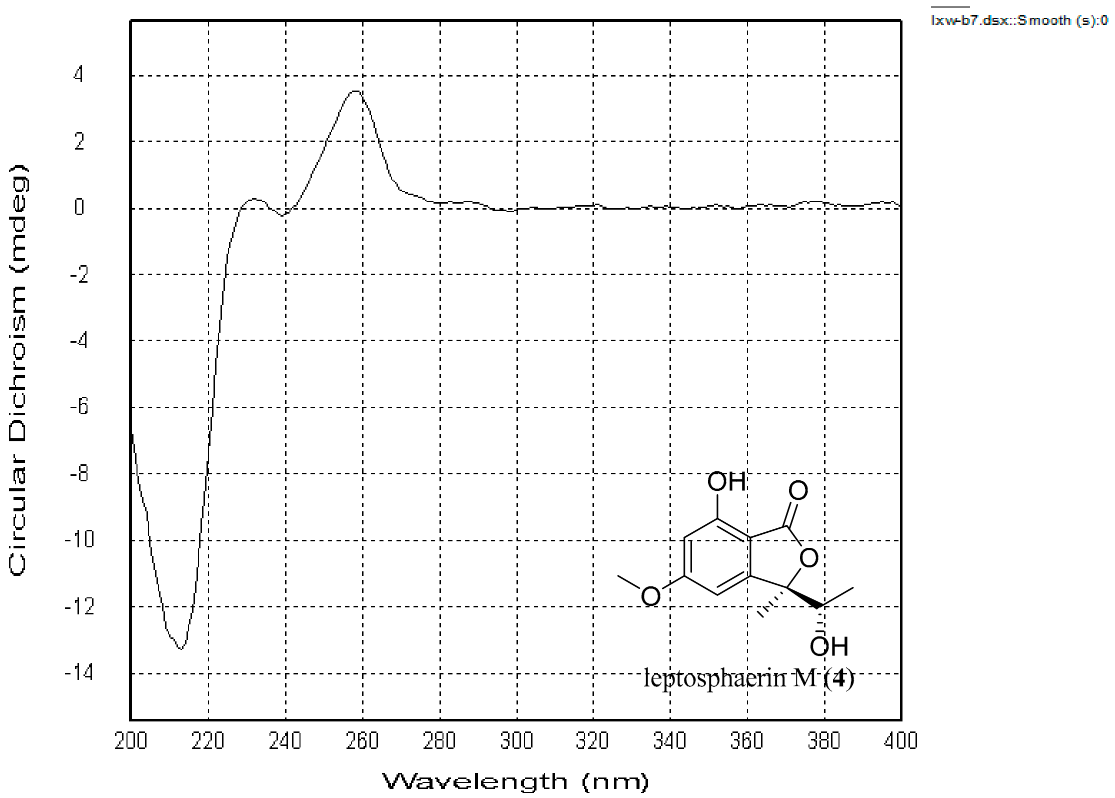

Figure S34. UV and CD spectrum of leptosphaerin M (**4**)

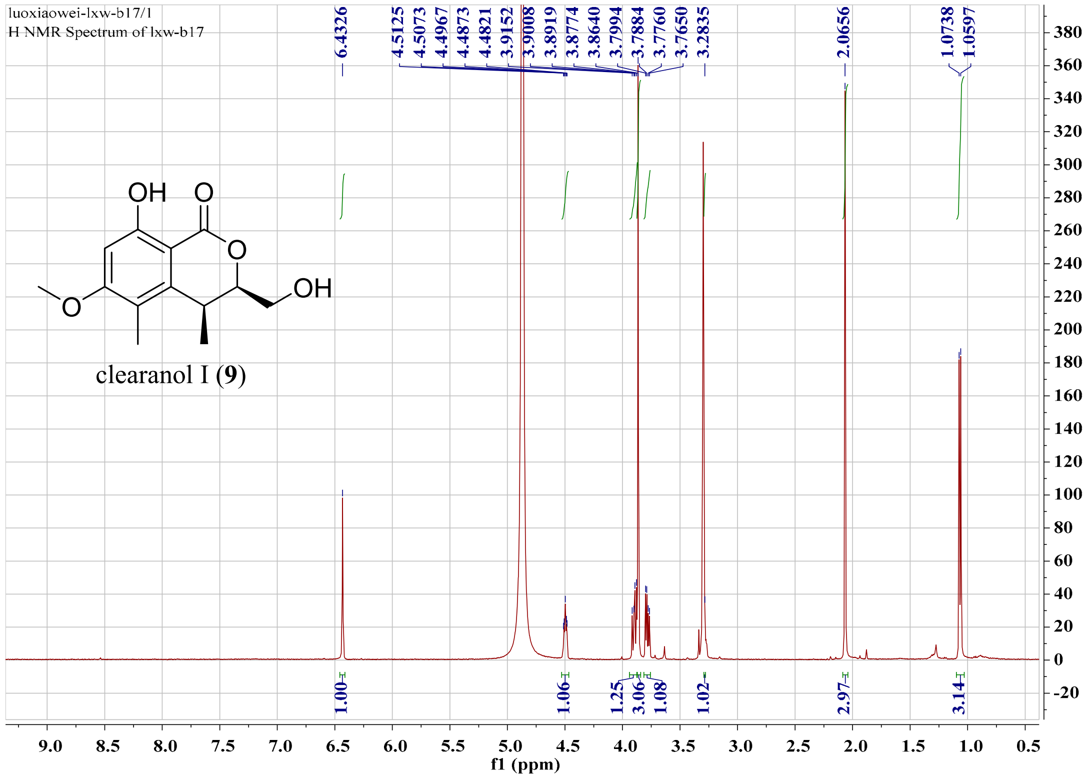

Figure S35. ^{1}H NMR spectrum of clearanol I (**9**) (MeOD)

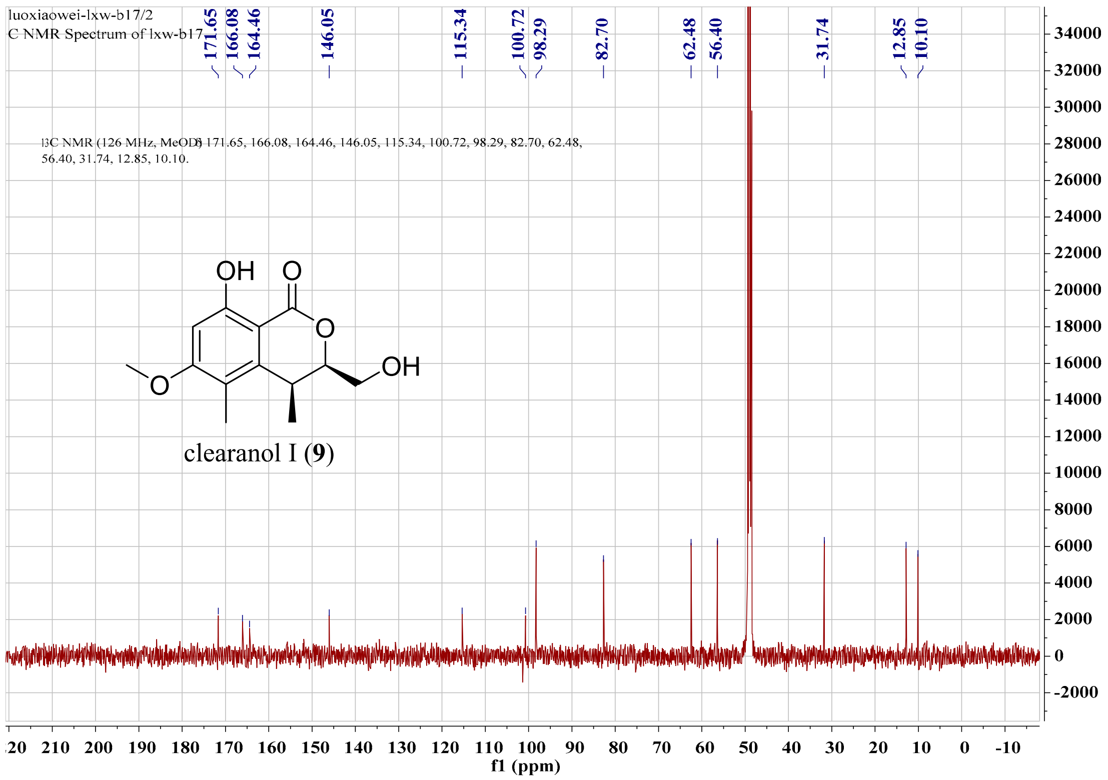

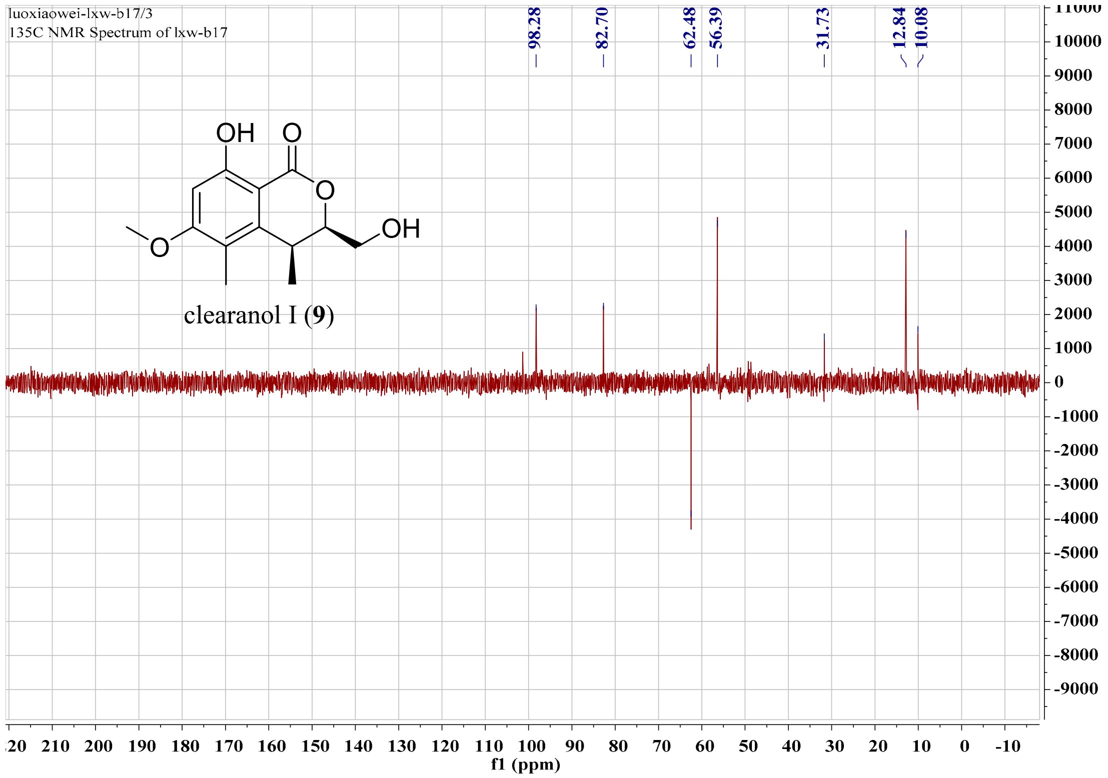

Figure S36. ^{13}C NMR and DEPT spectrum of clearanol I (**9**) (MeOD)

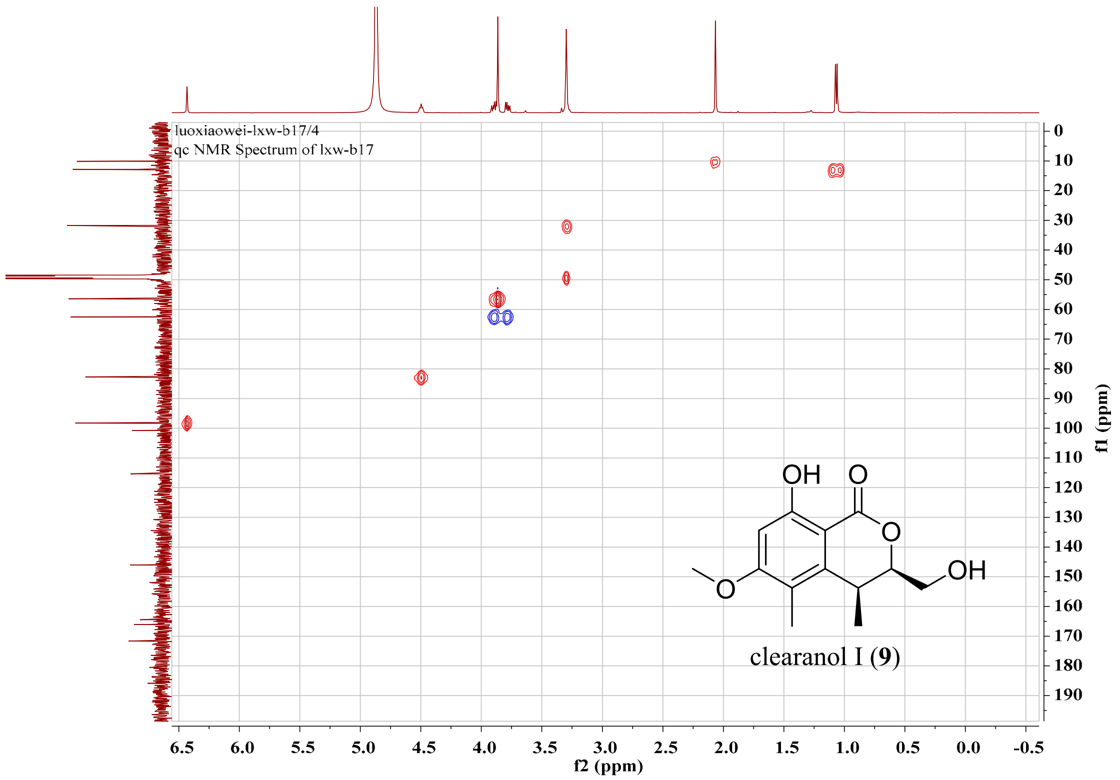

Figure S37. HSQC spectrum of clearanol I (**9**) (MeOD)

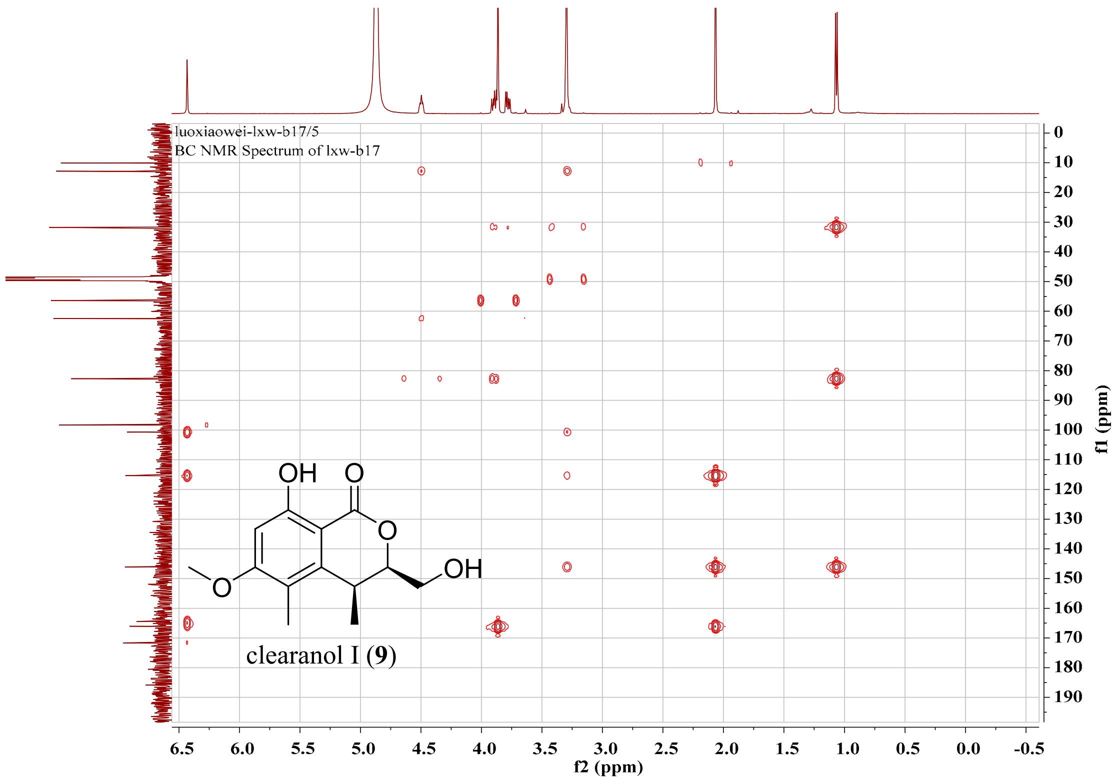

Figure S38. HMBC spectrum of clearanol I (**9**) (MeOD)

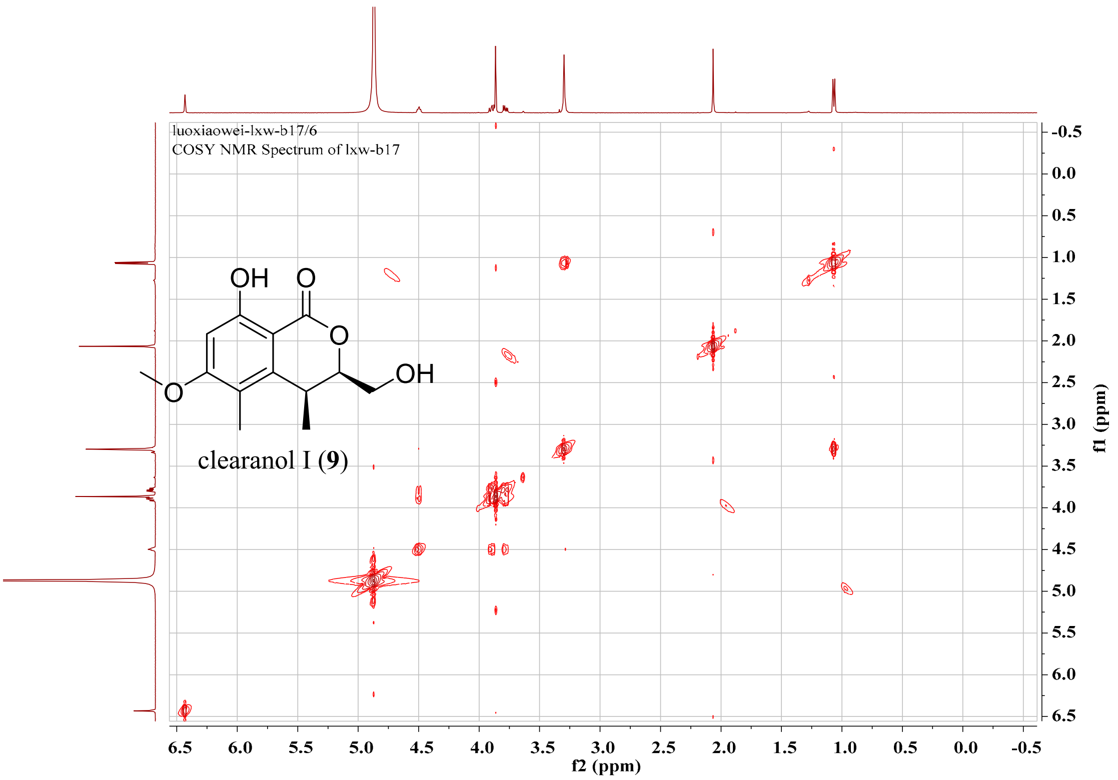

Figure S39. ^{1}H-^{1}H COSY spectrum of clearanol I (**9**) (MeOD)

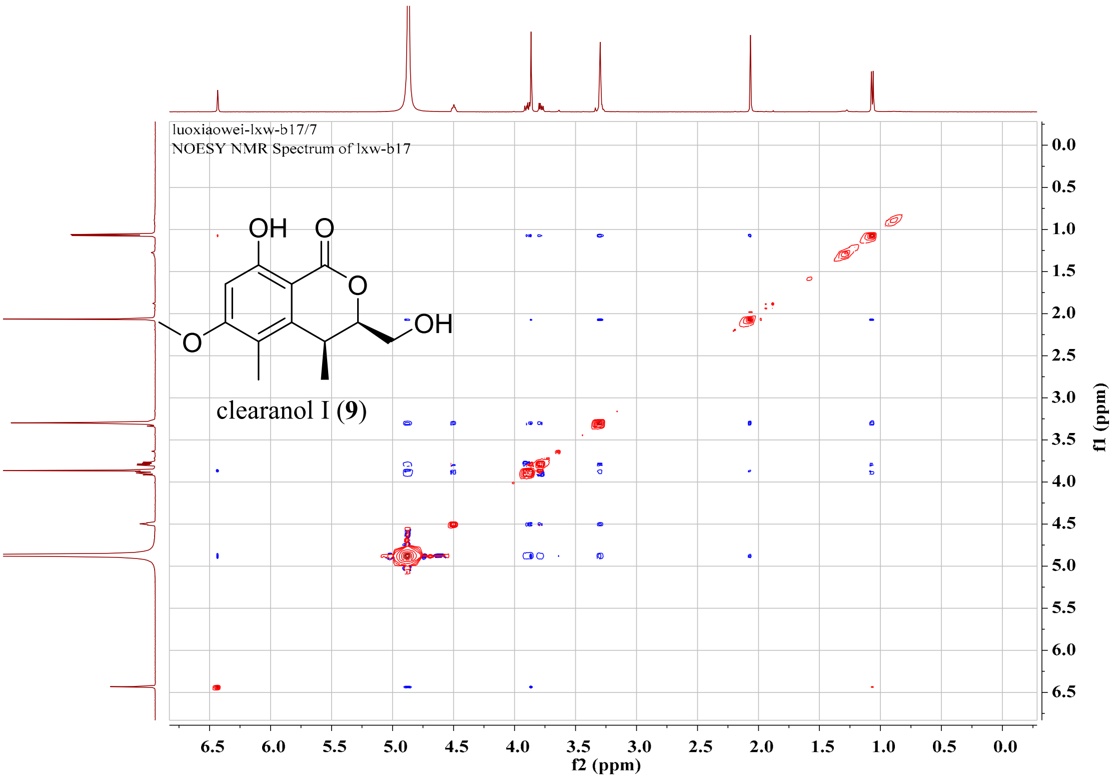

Figure S40. NOESY spectrum of clearanol I (**9**) (MeOD)

Mass Spectrum SmartFormula Report

Analysis Info

Analysis Name	D:\Data\MS\data\201605\luoxiaowei_lxw-b17_pos_31_01_1728.d	Acquisition Date	5/5/2016 4:13:04 PM
Method	LC_Direct Infusion_pos_100-1000mz.m	Operator	SCSIO
Sample Name	luoxiaowei_lxw-b17_pos	Instrument / Ser#	maXis 29
Comment			

Acquisition Parameter

Source Type	ESI	Ion Polarity	Positive	Set Nebulizer	0.4 Bar
Focus	Active	Set Capillary	4500 V	Set Dry Heater	180 °C
Scan Begin	100 m/z	Set End Plate Offset	-500 V	Set Dry Gas	4.0 l/min
Scan End	2000 m/z	Set Collision Cell RF	800.0 Vpp	Set Divert Valve	Waste

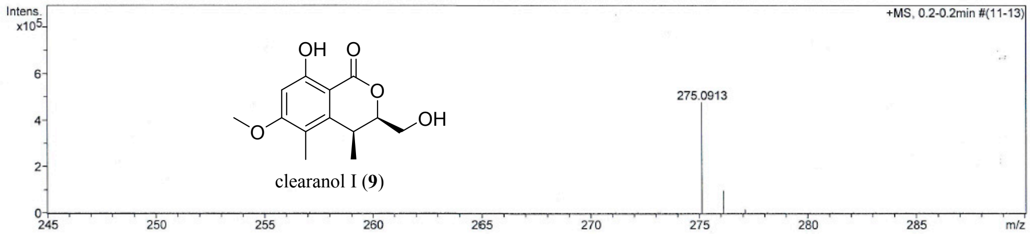

Meas. m/z	#	Formula	Score	m/z	err [ppm]	err [mDa]	mSigma	rdb	e⁻ Conf	N-Rule
253.1077	1	C 13 H 17 O 5	100.00	253.1071	-2.5	-0.6	103.1	5.5	even	ok
275.0913	1	C 13 H 16 Na O 5	19.76	275.0890	-8.3	-2.3	37.9	5.5	even	ok
527.1896	1	C 26 H 32 Na O 10	98.31	527.1888	-1.6	-0.8	87.6	10.5	even	ok

Figure S41. HRESIMS spectrum of clearanol I (**9**)

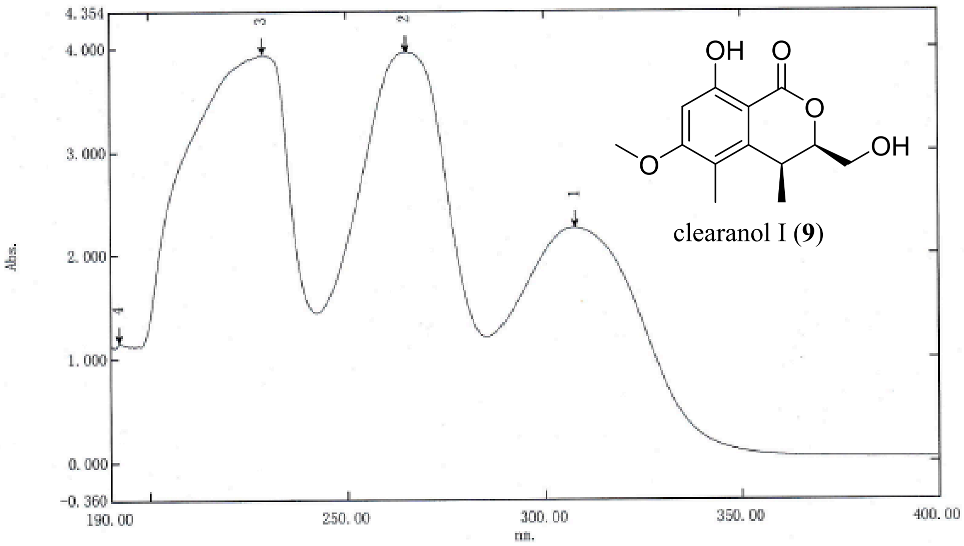

[测定属性]
波长范围 (nm.): 190.00 到 400.00
扫描速度: 中速
采样间隔: 0.2
自动采样间隔: 启用
扫描模式: 单个

[仪器属性]
仪器类型: UV-2600 系列
测定方式: 吸收值
狭缝宽: 2.0
积分时间: 0.1 秒.
光源转换波长: 323.0 nm
检测器单元: 直接
S/R 转换: 标准
阶梯校正: OFF

No.	波长(nm)	吸收值	描述
1	308.40	2.257	
2	265.60	3.958	
3	229.00	3.939	
4			

Figure S42. UV spectrum of clearanol I (**9**)

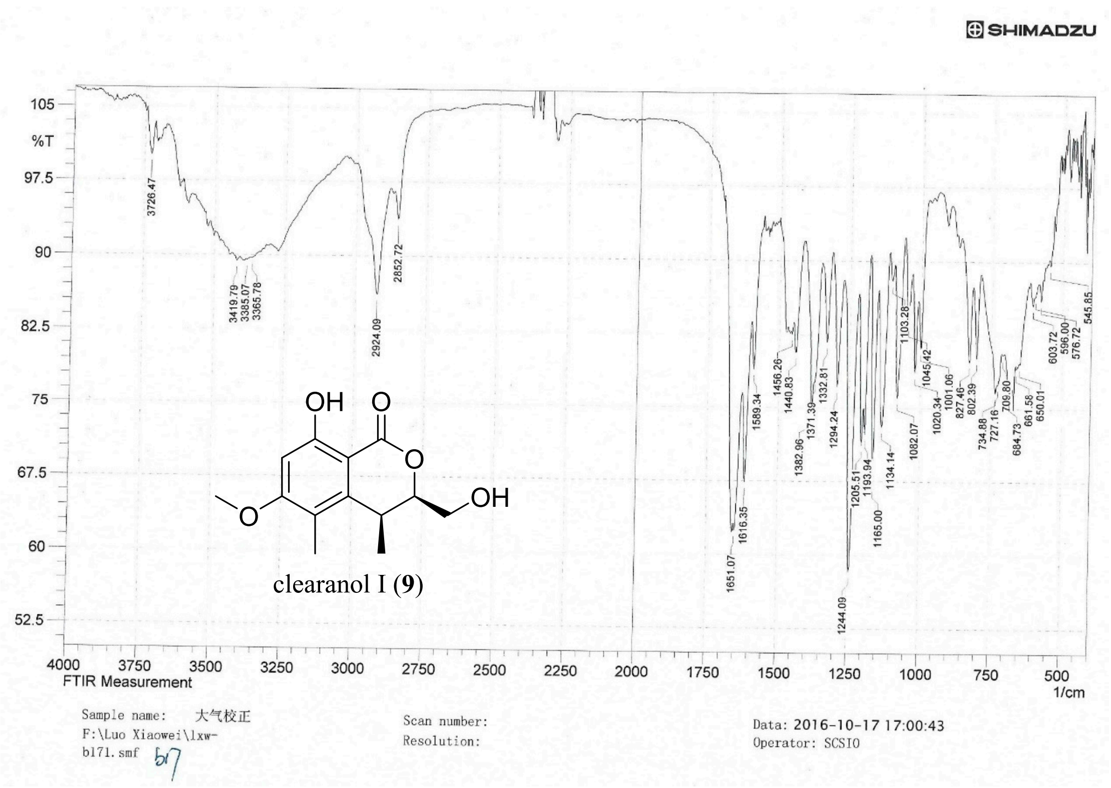

Figure S43. IR spectrum of clearanol I (**9**)

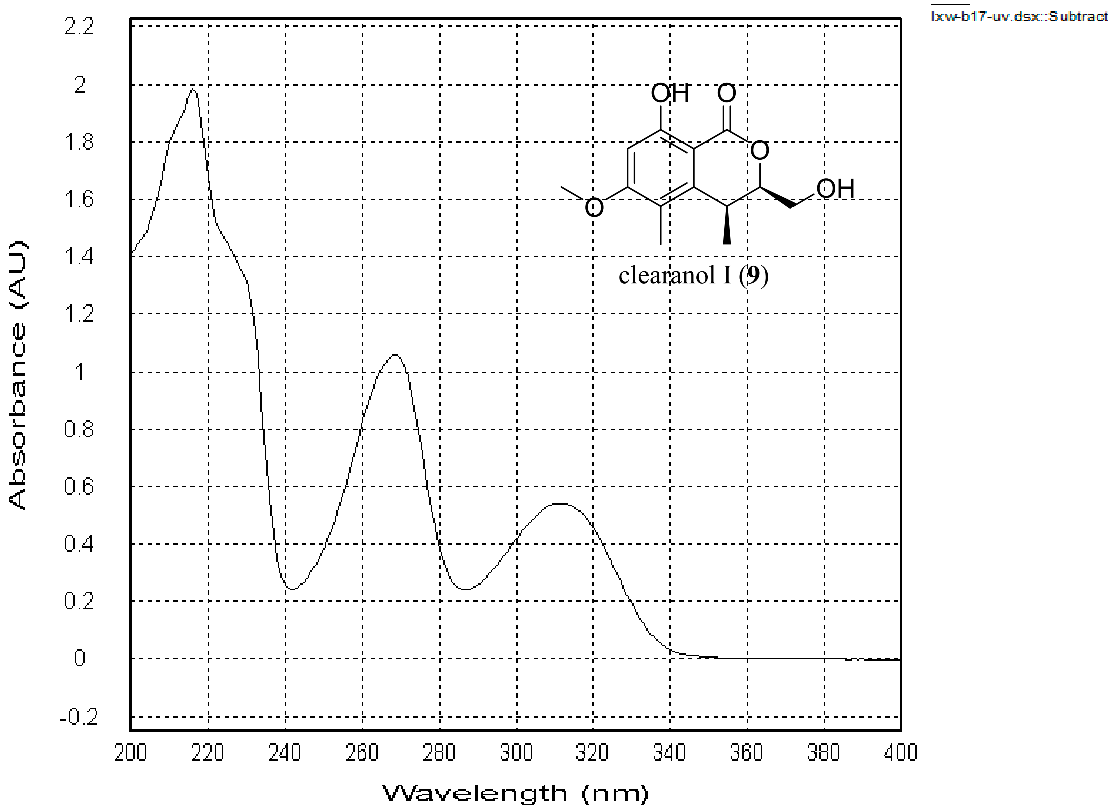

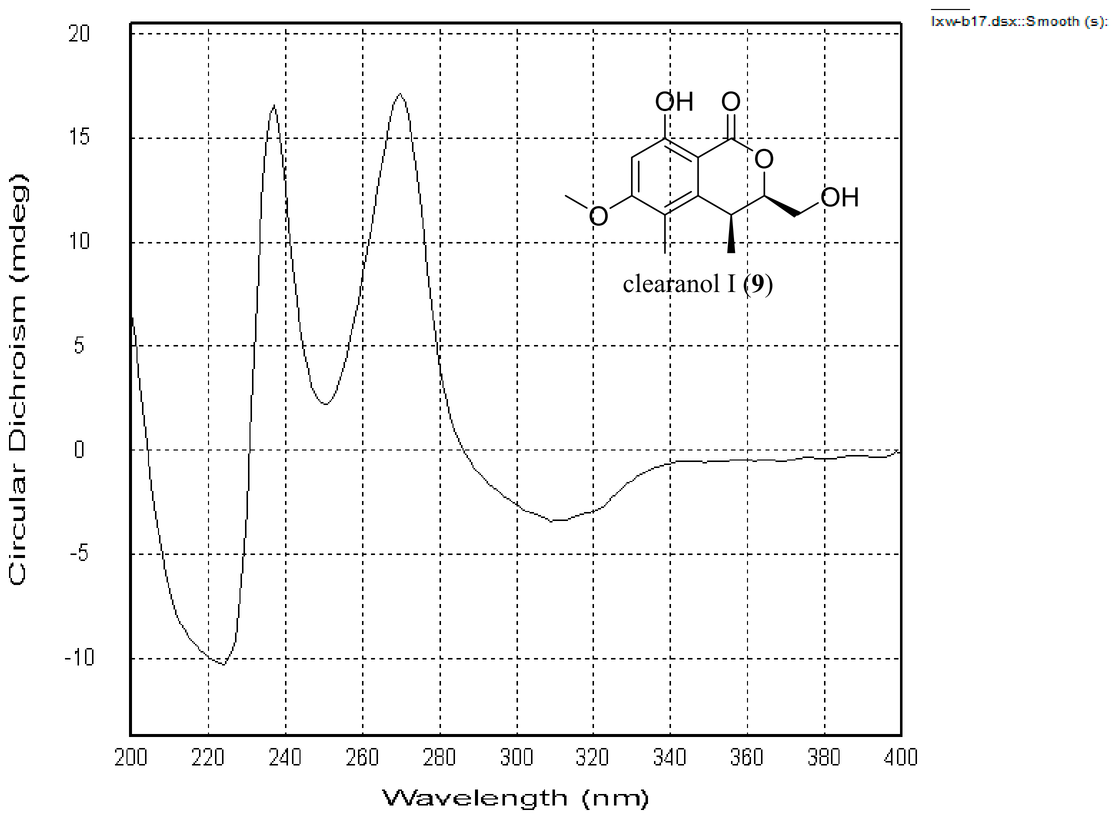

Figure S44. UV and CD spectrum of clearanol I (**9**)

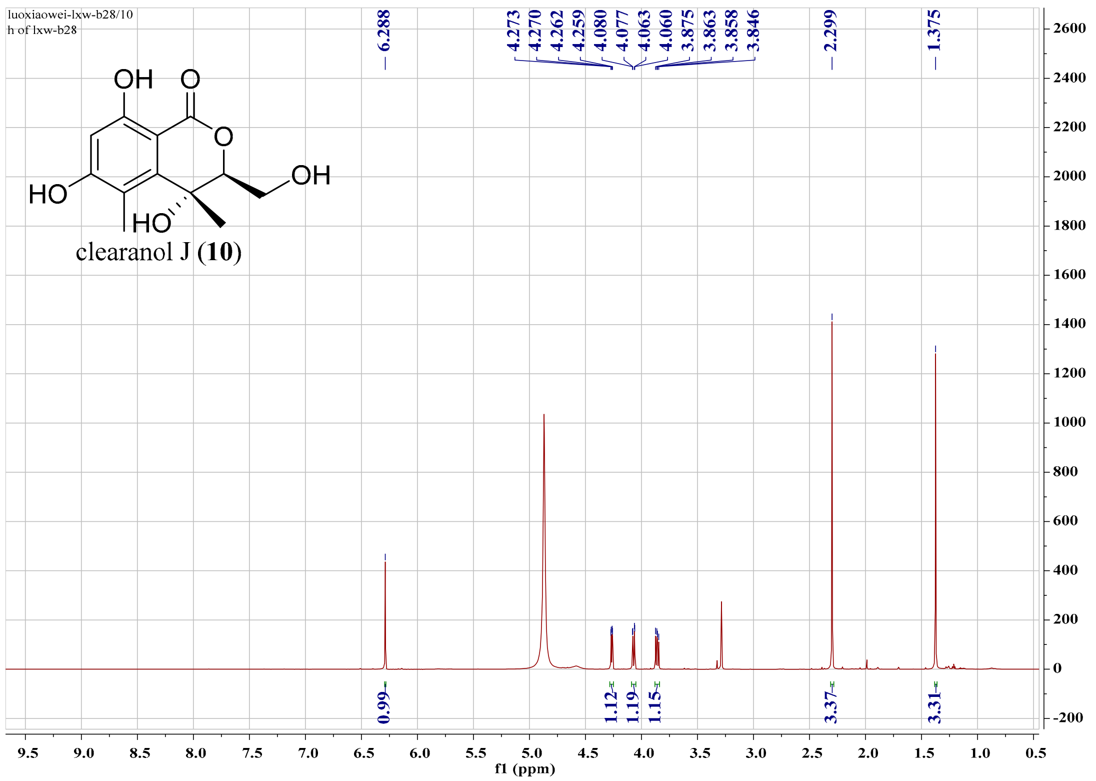

Figure S45. ^{1}H NMR spectrum of clearanol J (**10**) (MeOD)

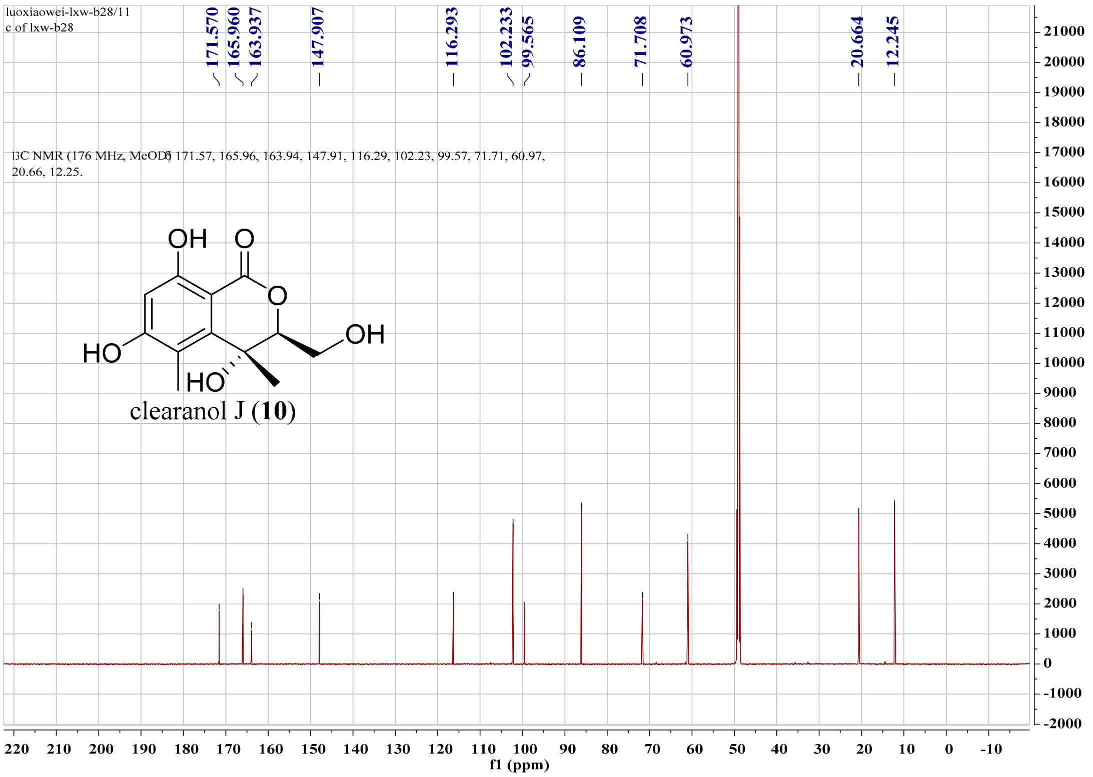

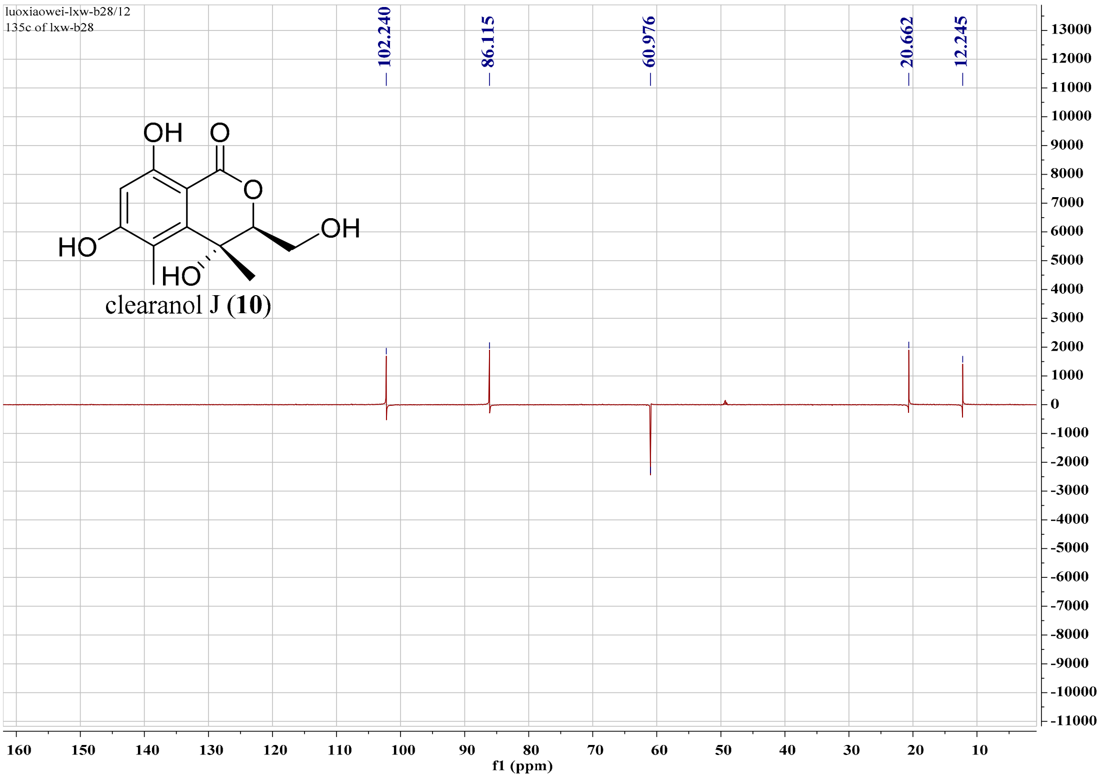

Figure S46. ^{13}C NMR and DEPT spectrum of clearanol J (**10**) (MeOD)

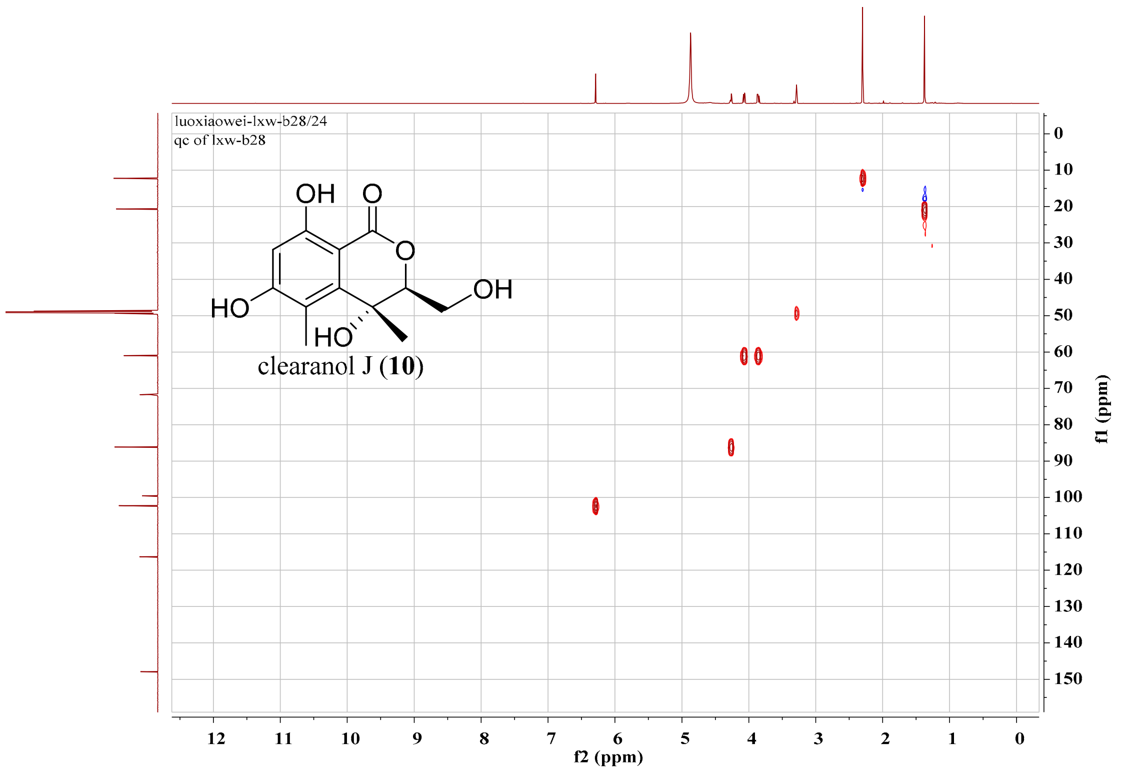

Figure S47. HSQC spectrum of clearanol J (**10**) (MeOD)

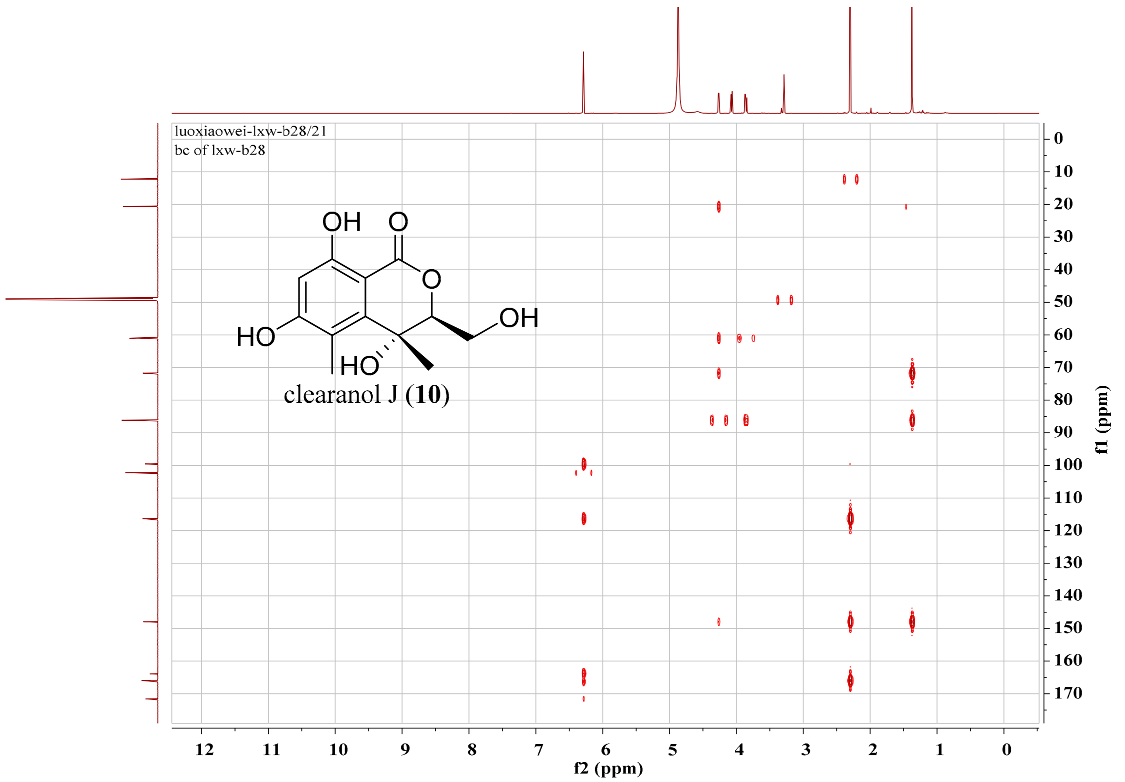

Figure S48. HMBC spectrum of clearanol J (**10**) (MeOD)

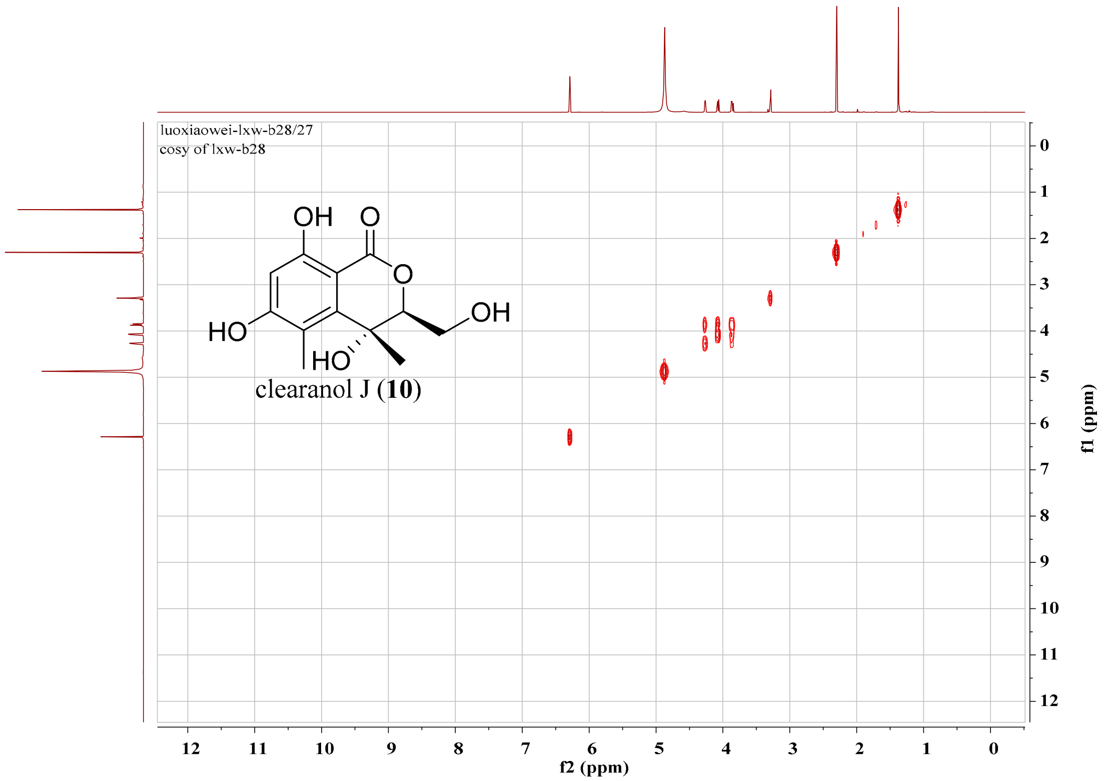

Figure S49. ^{1}H-^{1}H COSY spectrum of clearanol J (**10**) (MeOD)

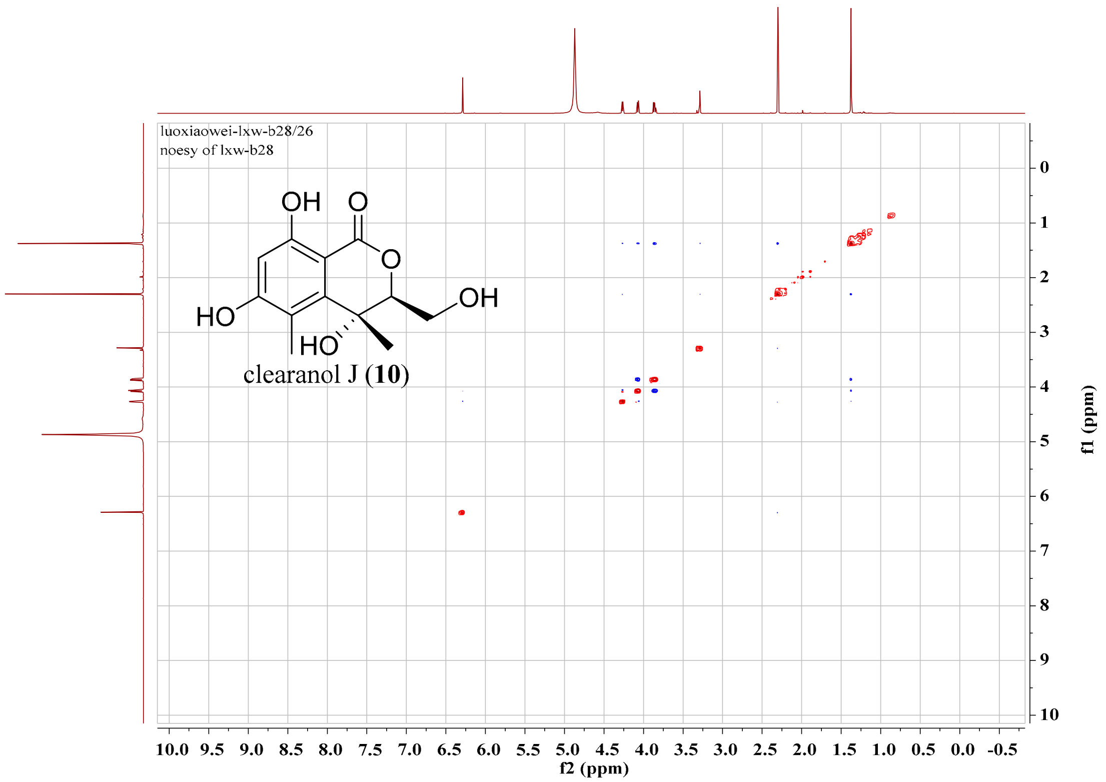

Figure S50. NOESY spectrum of clearanol J (**10**) (MeOD)

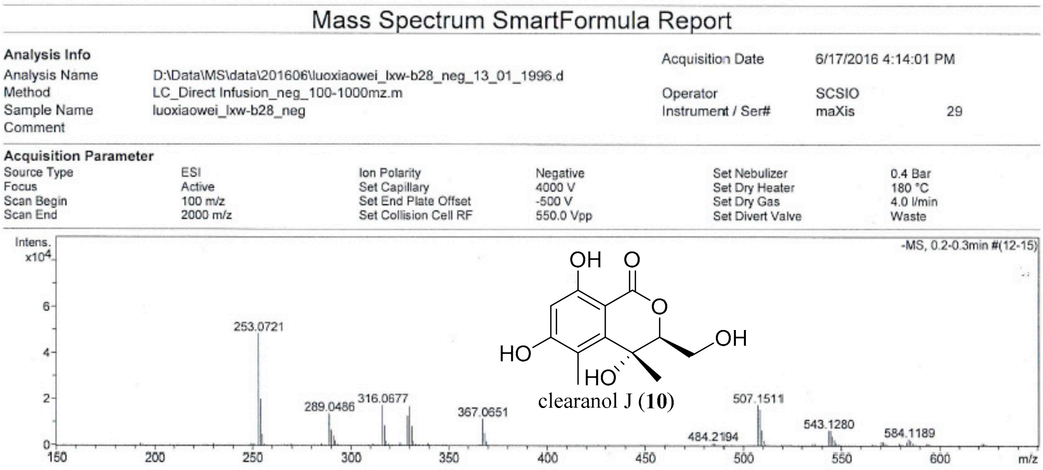

Meas. m/z	#	Formula	Score	m/z	err [ppm]	err [mDa]	mSigma	rdb	e⁻ Conf	N-Rule
253.0721	1	C 12 H 13 O 6	80.63	253.0718	-1.5	-0.4	163.1	6.5	even	ok
289.0486	1	C 12 H 14 Cl O 6	100.00	289.0484	-0.7	-0.2	165.7	5.5	even	ok
507.1511	1	C 24 H 27 O 12	0.02	507.1508	-0.6	-0.3	352.6	11.5	even	ok

Figure S51. HRESIMS spectrum of clearanol J (**10**)

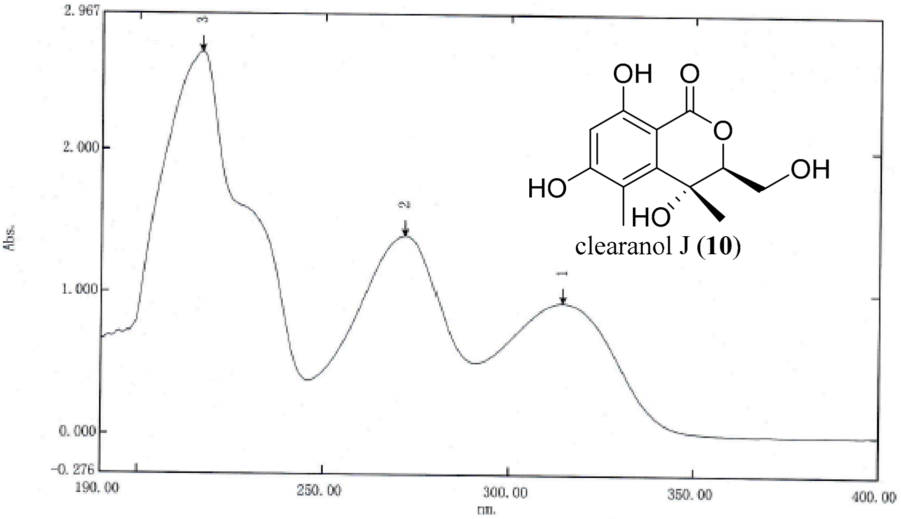

[测定属性]
波长范围 (nm.): 190.00 到 400.00
扫描速度: 中速
采样间隔: 0.2
自动采样间隔: 启用
扫描模式: 单个

[仪器属性]
仪器类型: UV-2600 系列
测定方式: 吸收值
狭缝宽: 2.0
积分时间: 0.1 秒.
光源转换波长: 323.0 nm
检测器单元: 直接
S/R 转换: 标准
阶梯校正: OFF

No.	波长(nm)	吸收值	描述
1	314.60	0.929	
2	271.80	1.401	
3	229.40	1.591	
4	216.80	2.697	
5	245.80	0.379	
6	290.00	0.510	
7			

Figure S52. UV spectrum of clearanol J (**10**)

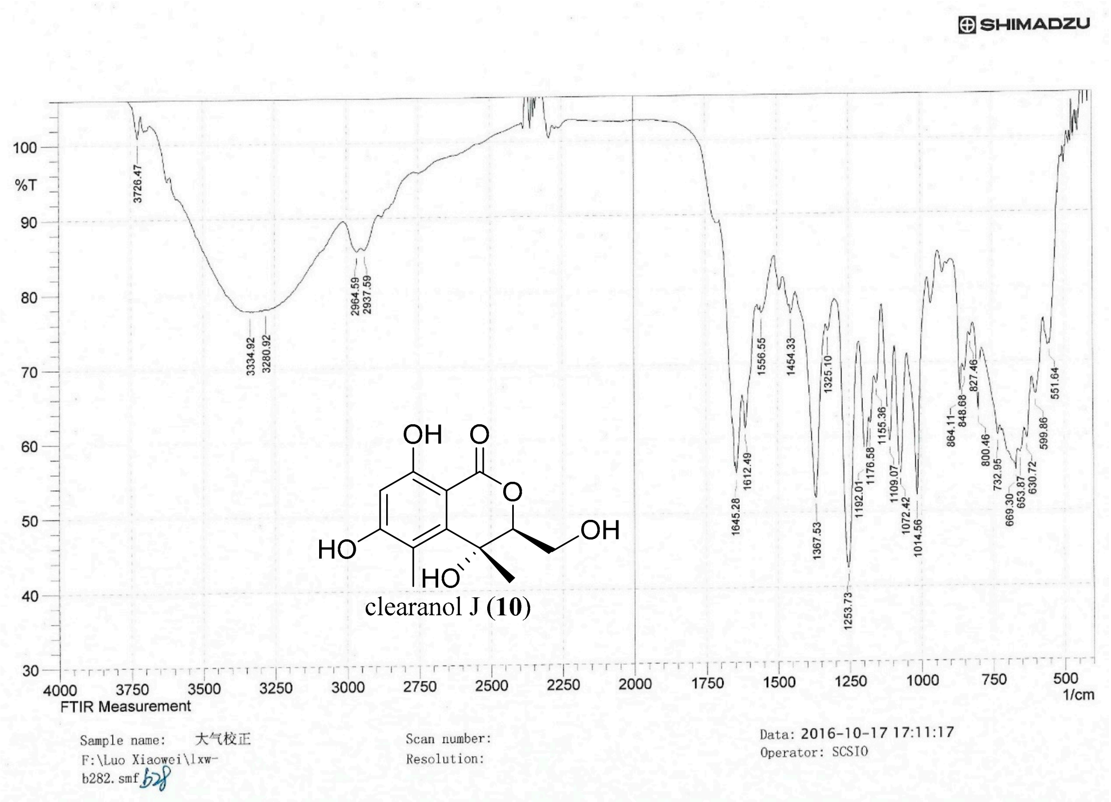

Figure S53. IR spectrum of clearanol J (**10**)

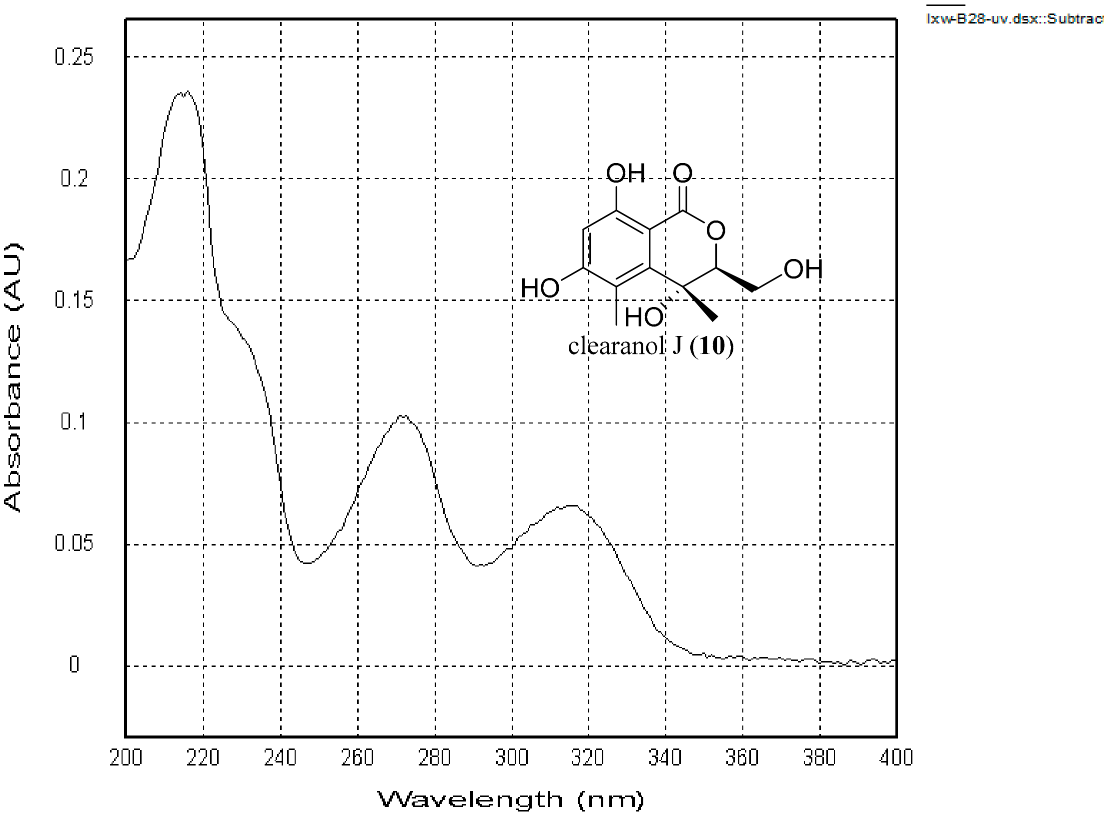

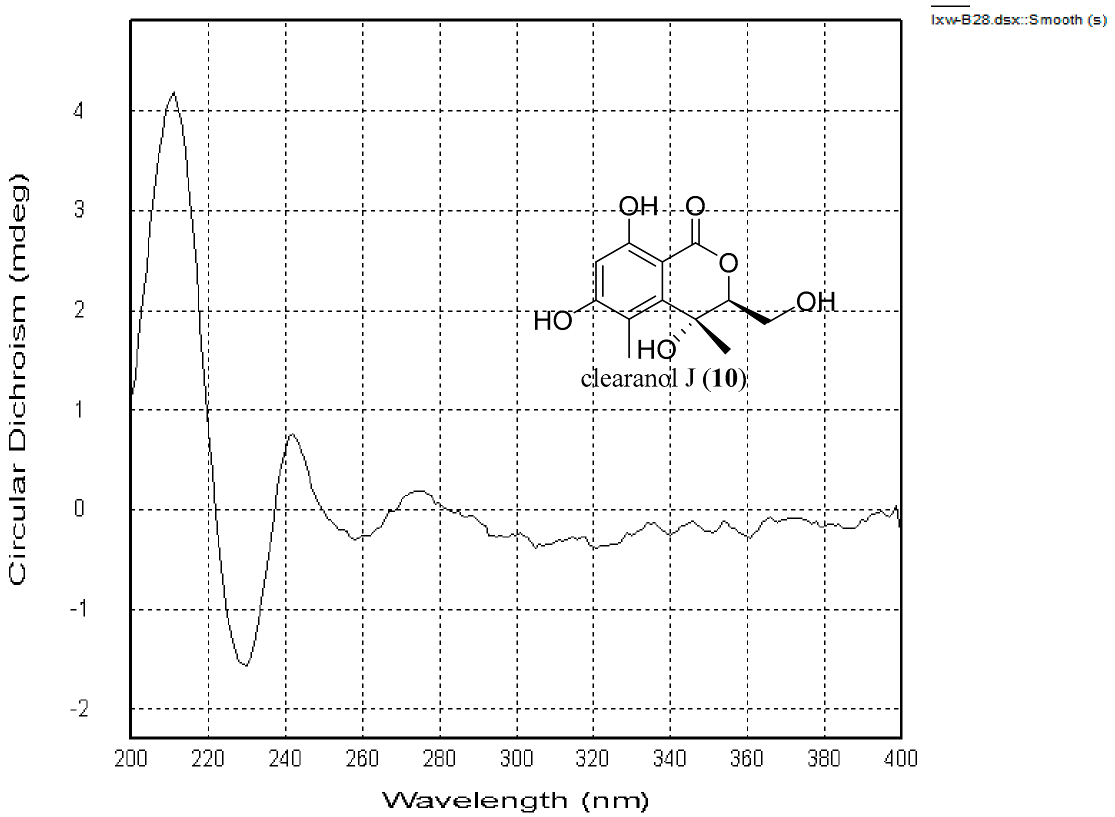

Figure S54. UV and CD spectrum of clearanol J (**10**)

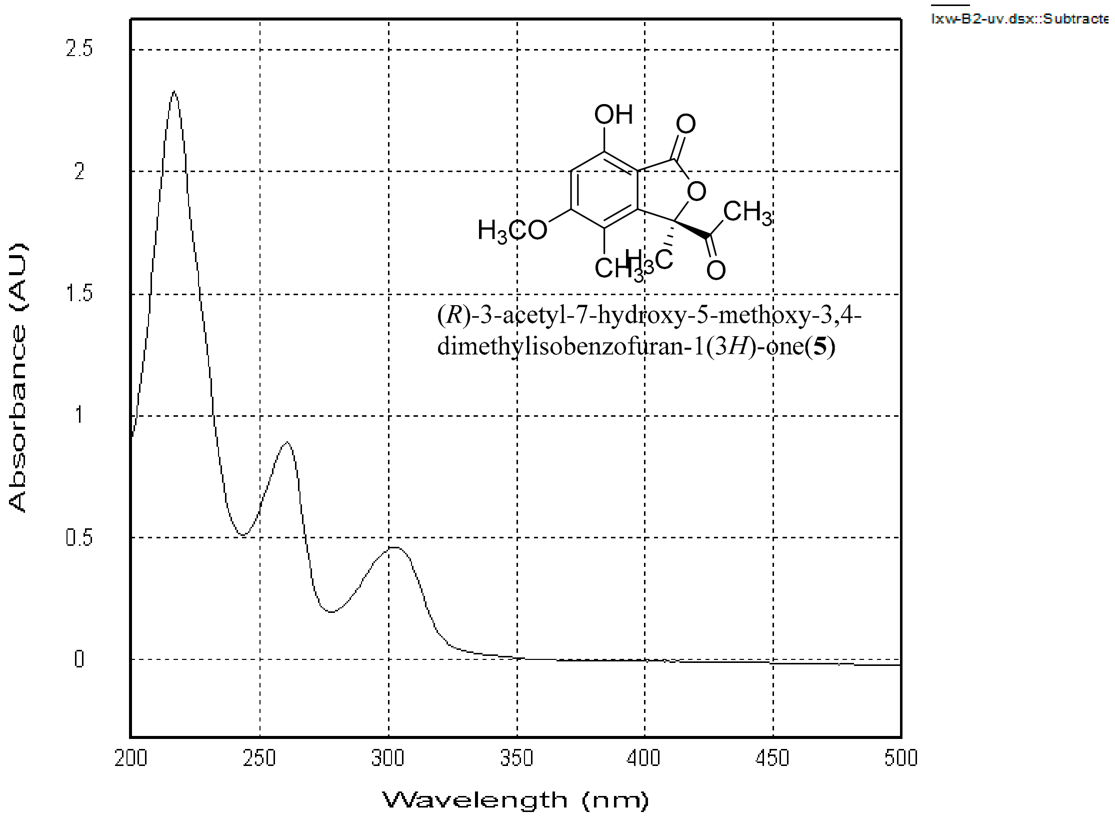

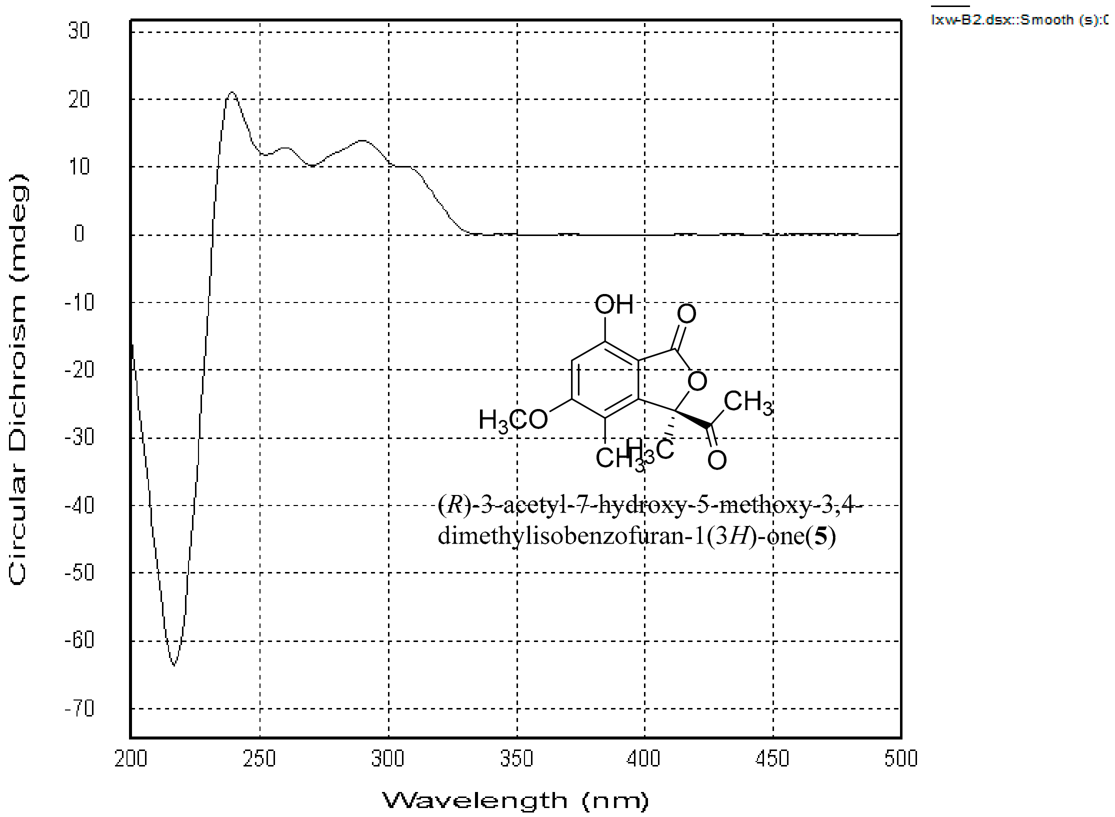

Figure S55. UV and CD spectrum of (*R*)-3-acetyl-7-hydroxy-5-methoxy-3,4-dimethylisobenzofuran-1(3*H*)-one (**5**)

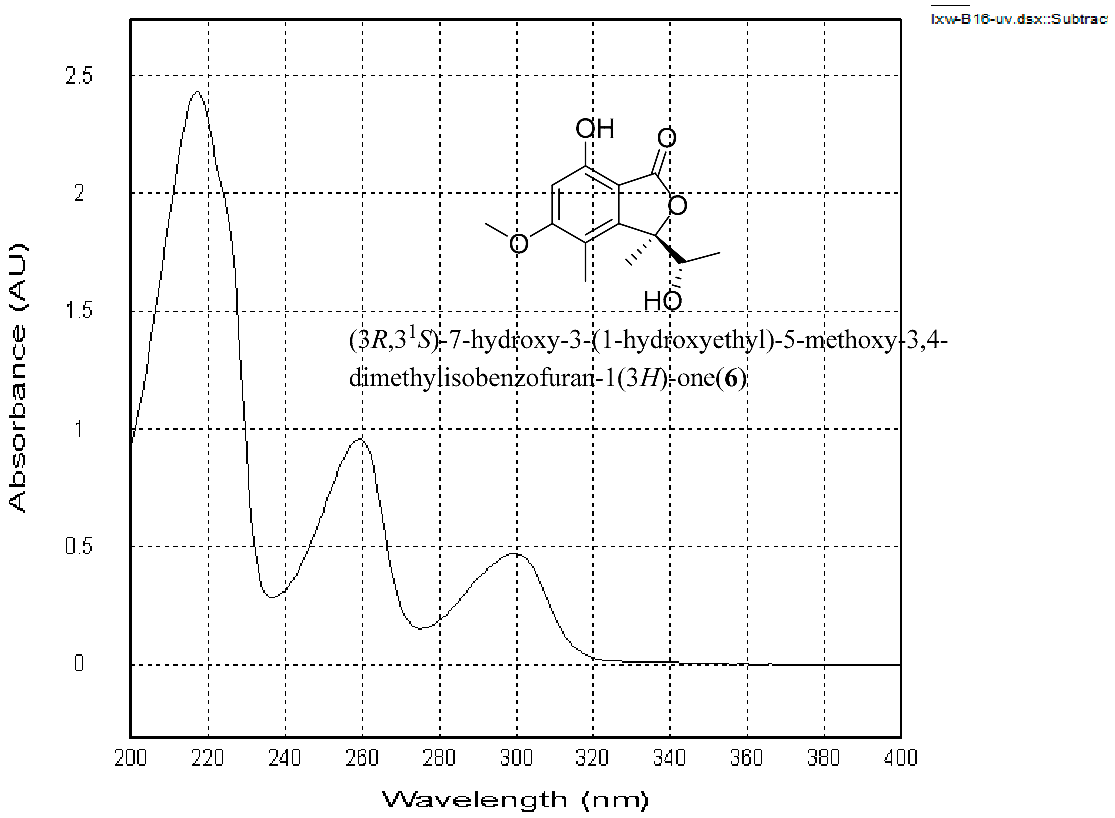

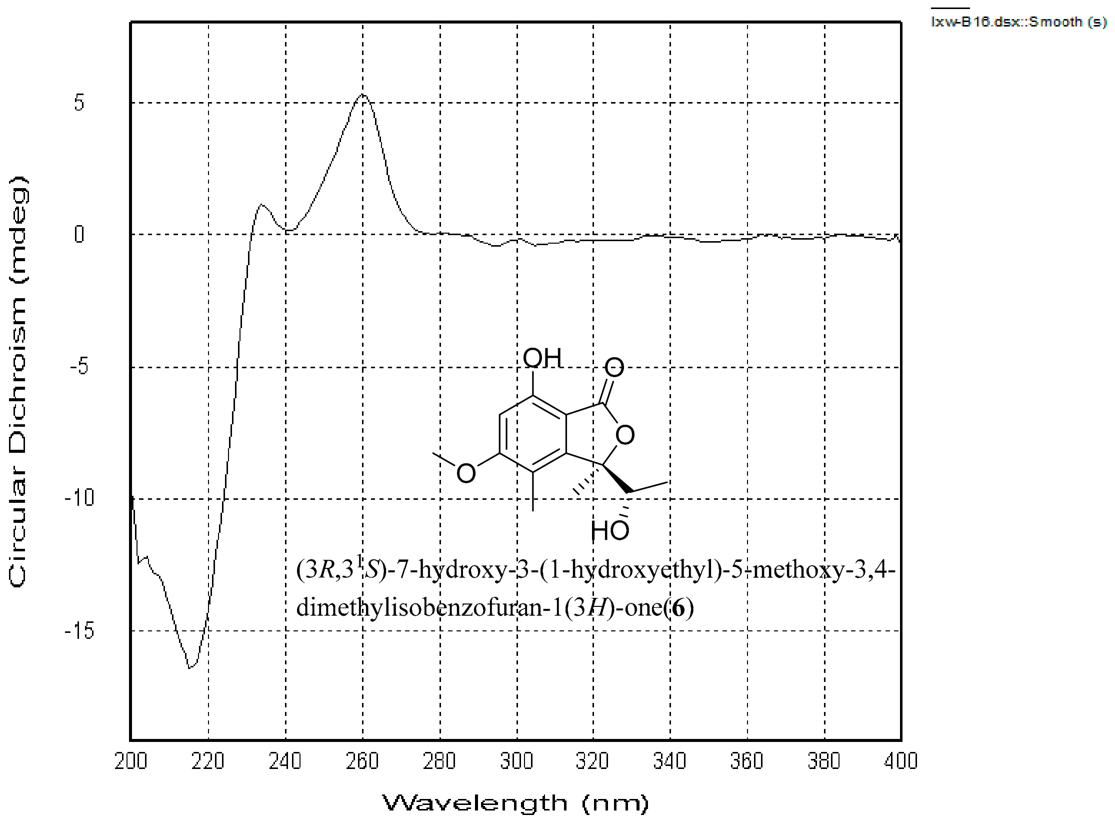

Figure S56. UV and CD spectrum of (3*R*,3[1]*S*)-7-hydroxy-3-(1-hydroxyethyl)-5-methoxy-3,4-dimethylisobenzofuran-1(3*H*)-one (**6**)

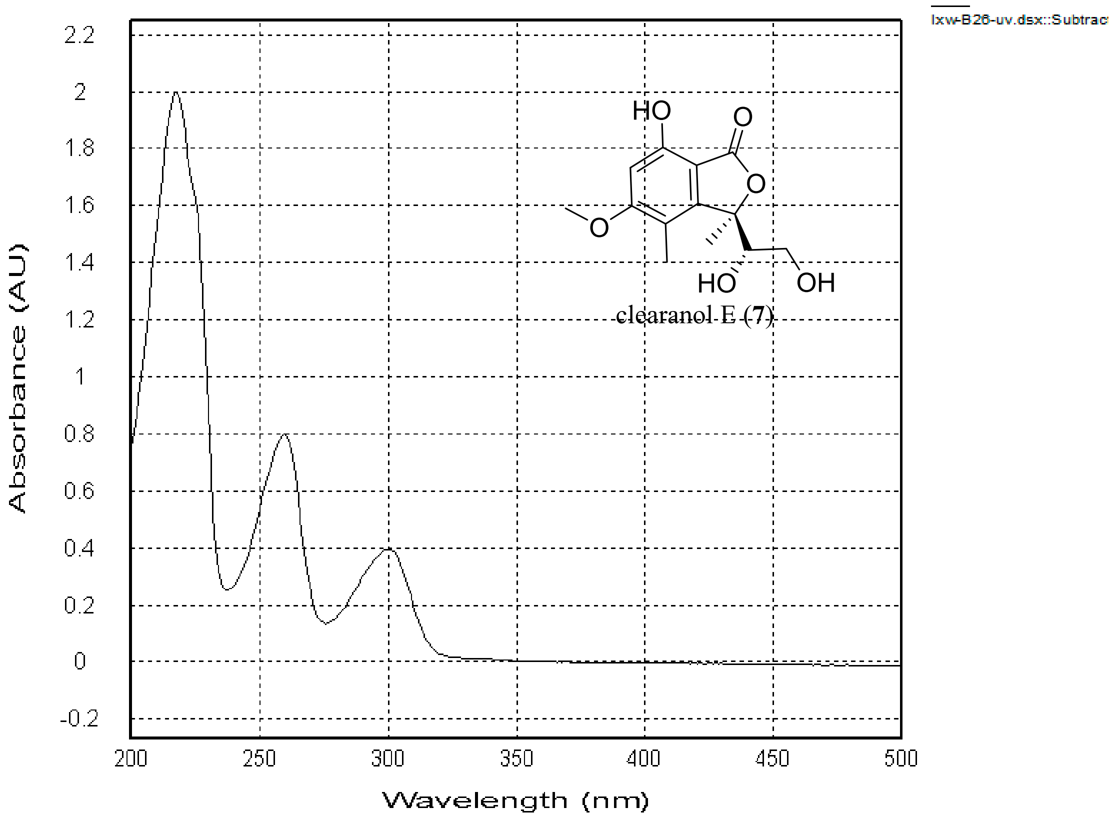

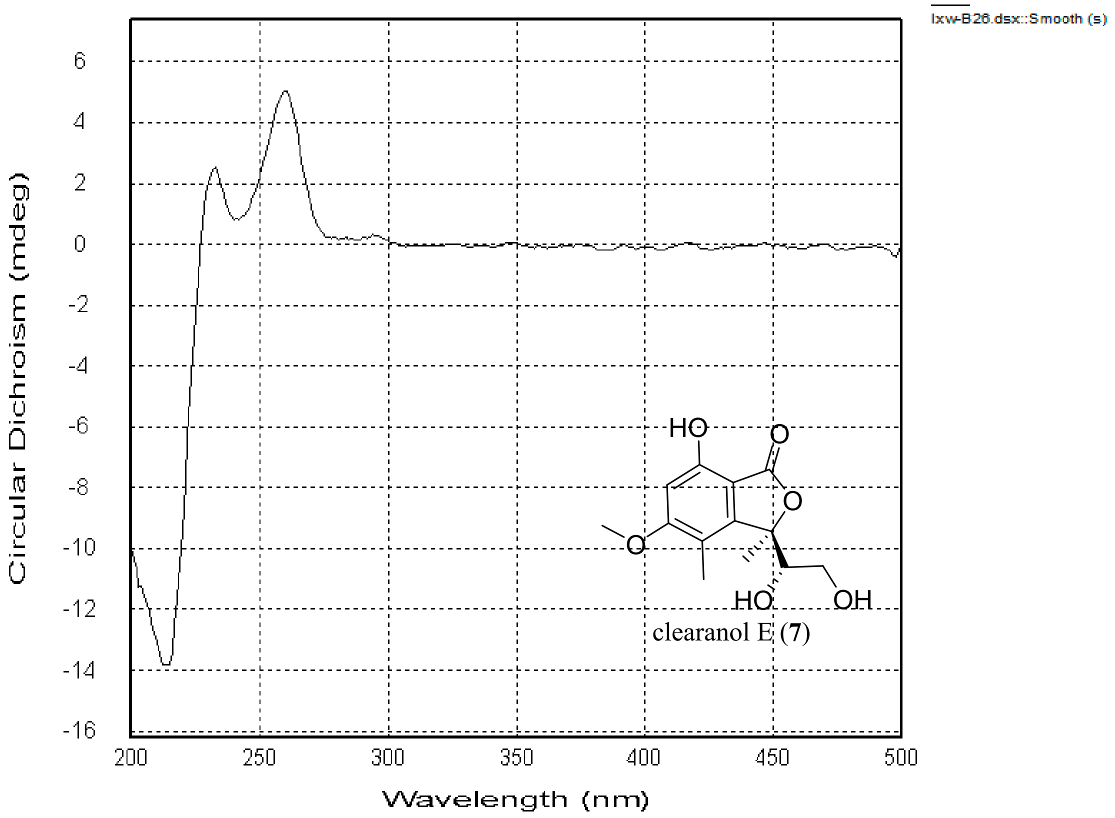

Figure S57. UV and CD spectrum of clearanol E (**7**)

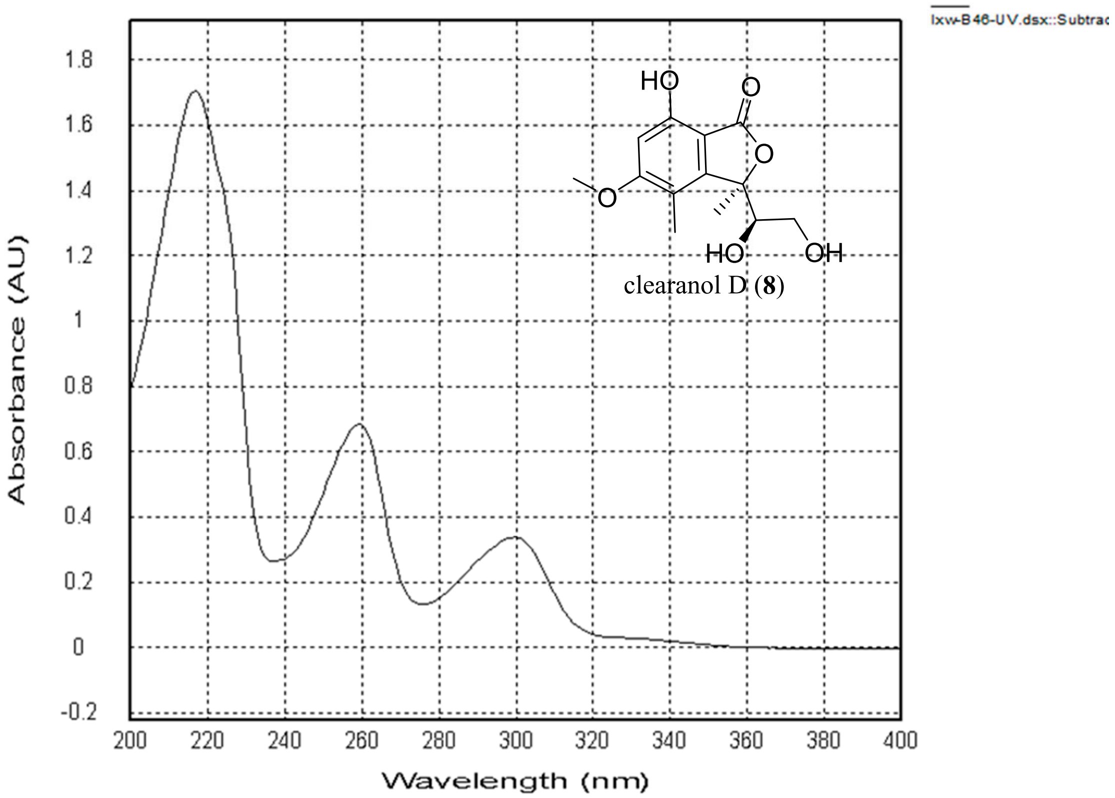

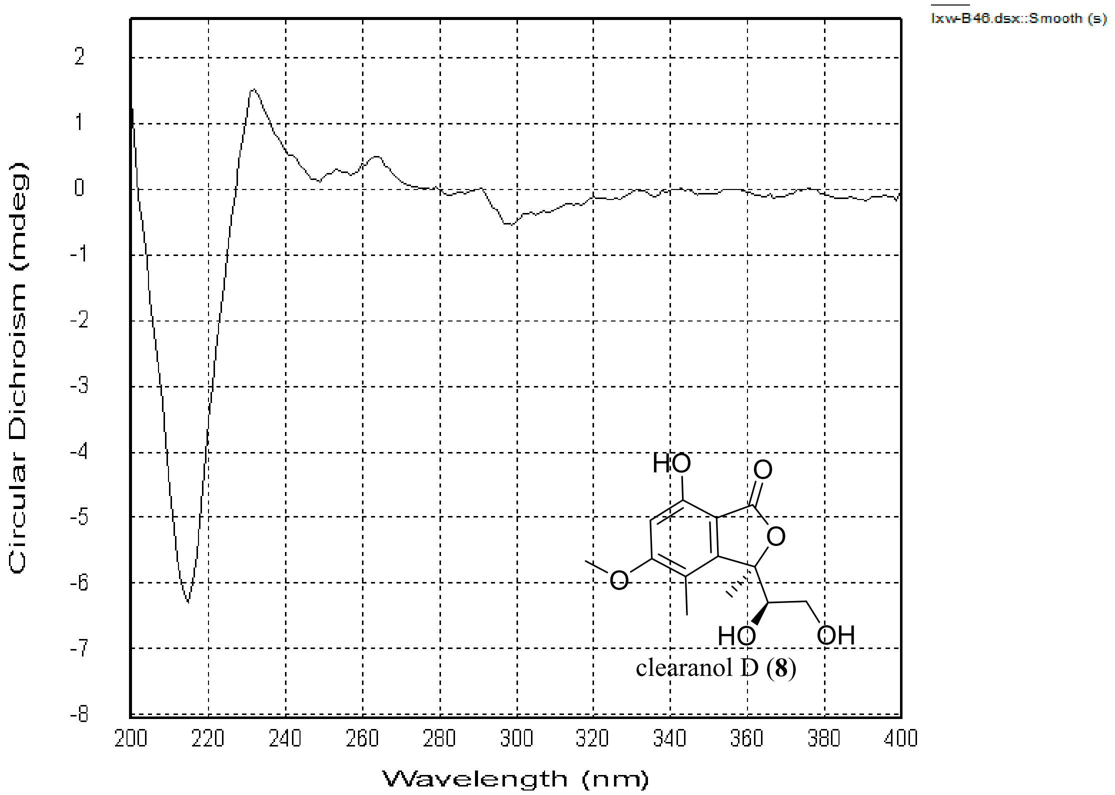

Figure S58. UV and CD spectrum of clearanol D (**8**)

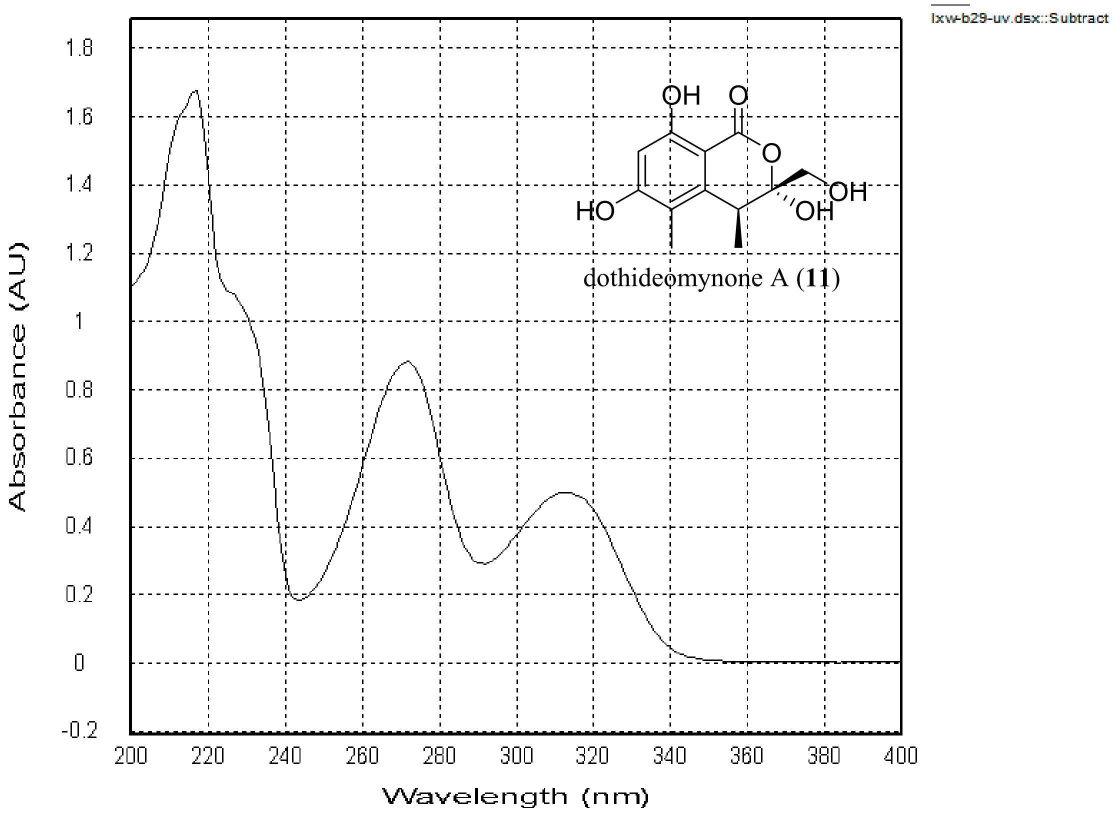

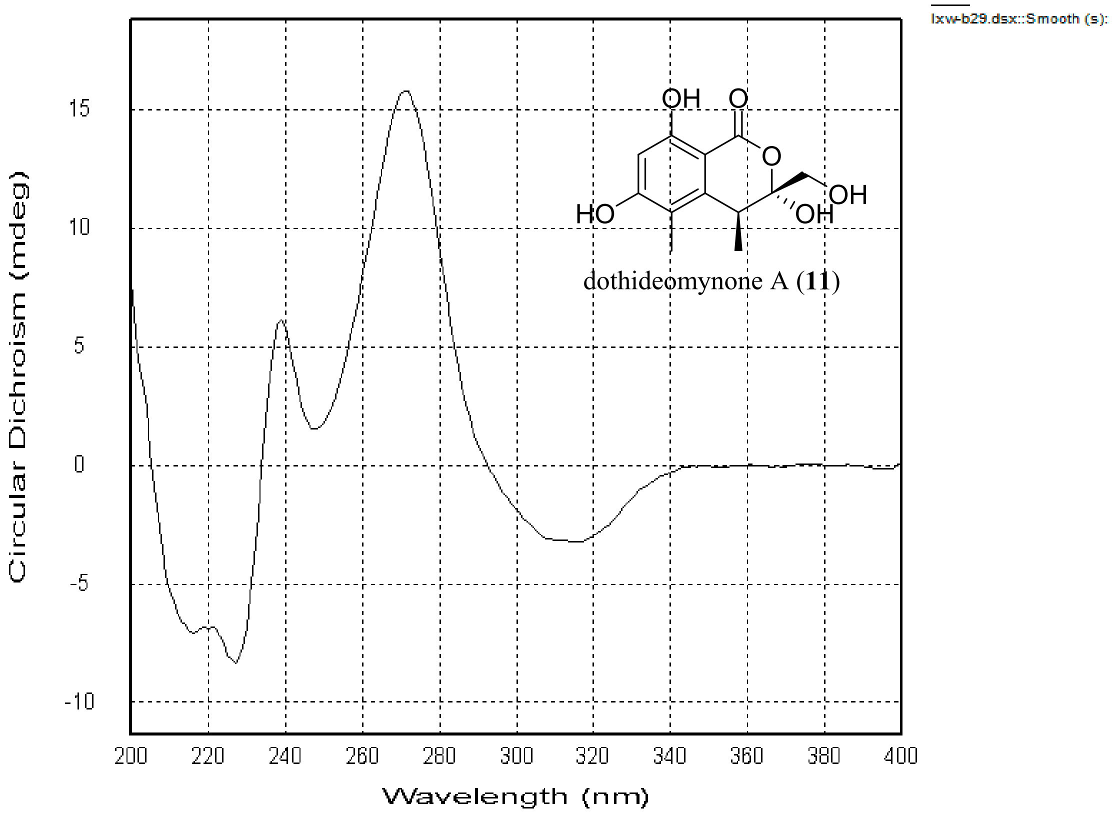

Figure S59. UV and CD spectrum of dothideomynone A (**11**)

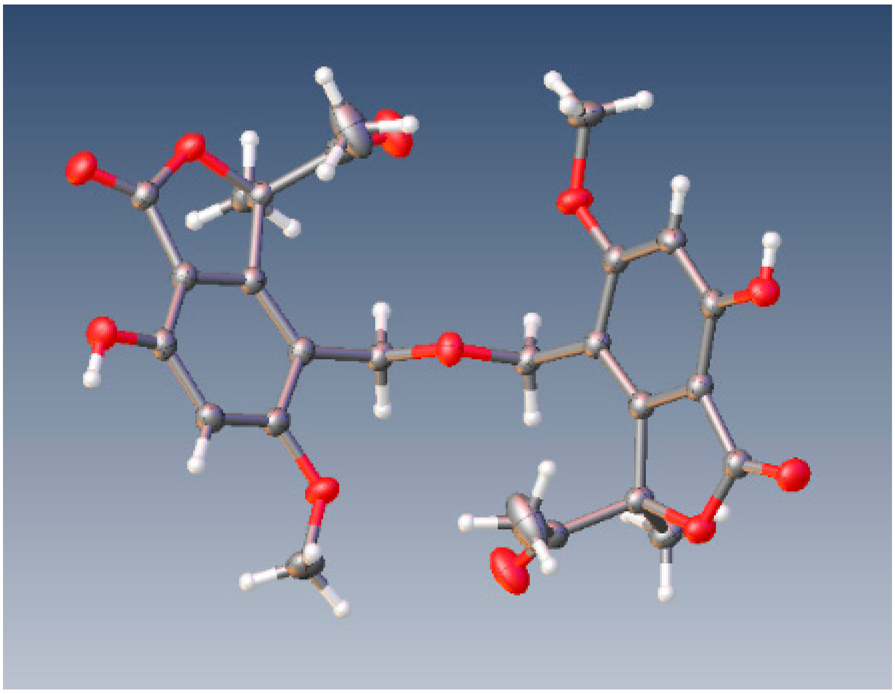

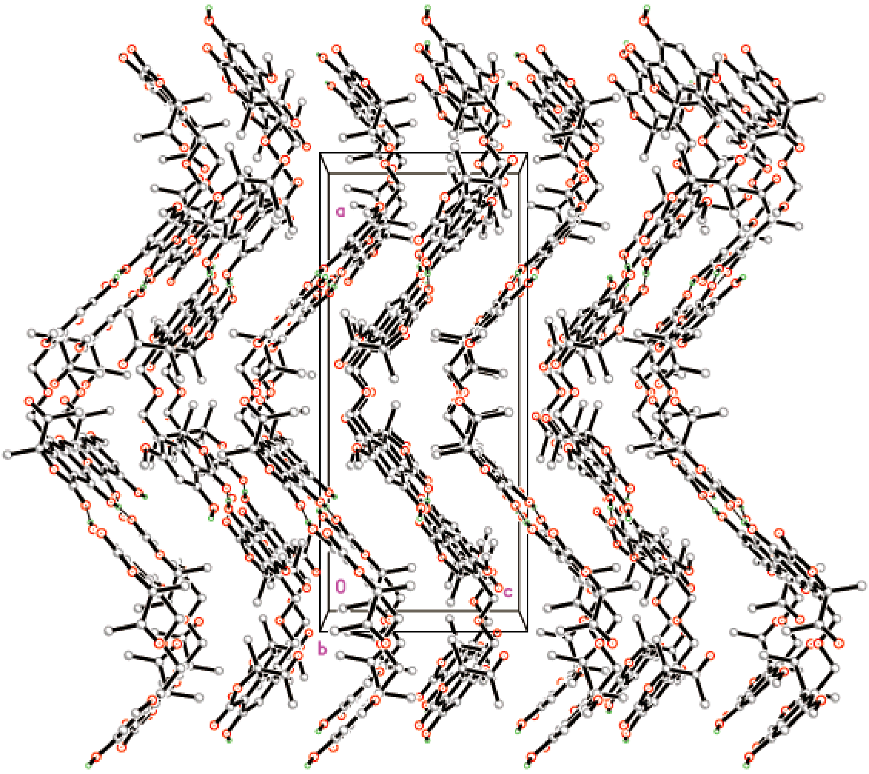
a
0
b
c

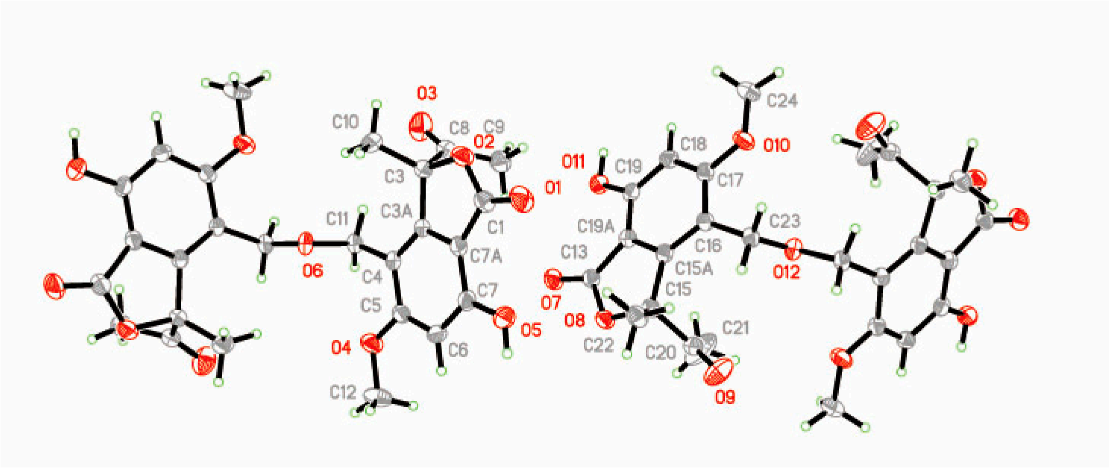

Figure S61. X-Ray structure of leptosphaerin J (**1**)

Table S1. Crystal data and structure refinement for leptosphaerin J (**1**)

Identification code	1606215	
Empirical formula	$C_{26}H_{26}O_{11}$	
Formula weight	514.47	
Temperature	150(2) K	
Wavelength	1.54184 Å	
Crystal system	Orthorhombic	
Space group	$P2_12_12$	
Unit cell dimensions	a = 23.08124(16) Å	α= 90°.
	b = 10.49438(8) Å	β= 90°.
	c = 10.03167(10) Å	γ = 90°.
Volume	2429.90(3) Å^3	
Z	4	
Density (calculated)	1.406 Mg/m^3	
Absorption coefficient	0.938 mm^{-1}	
F(000)	1080	
Crystal size	0.390 x 0.080 x 0.040 mm^3	
Theta range for data collection	3.830 to 74.081°.	
Index ranges	-28<=h<=28, -13<=k<=12, -11<=l<=12	
Reflections collected	23207	
Independent reflections	4841 [R(int) = 0.0236]	
Completeness to theta = 67.684°	99.9 %	
Absorption correction	Semi-empirical from equivalents	
Max. and min. transmission	1.00000 and 0.69119	
Refinement method	Full-matrix least-squares on F^2	
Data / restraints / parameters	4841 / 0 / 347	

Goodness-of-fit on F^2	1.031
Final R indices [I>2sigma(I)]	R1 = 0.0289, wR2 = 0.0758
R indices (all data)	R1 = 0.0297, wR2 = 0.0765
Absolute structure parameter	0.00(5)
Extinction coefficient	n/a
Largest diff. peak and hole	0.176 and -0.175 e.Å^{-3}

Table S2. Atomic coordinates (x 10^4) and equivalent isotropic displacement parameters (Å^{2}x 10^3) for leptosphaerin J (**1**). U (eq) is defined as one third of the trace of the orthogonalized U^{ij} tensor.

	x	y	z	U(eq)
C(1)	3297(1)	7145(2)	4488(2)	30(1)
C(3)	4108(1)	7062(2)	3109(2)	25(1)
C(3A)	3844(1)	8387(2)	3058(2)	22(1)
C(4)	4011(1)	9447(2)	2333(2)	22(1)
C(5)	3667(1)	10550(2)	2524(2)	24(1)
C(6)	3183(1)	10570(2)	3348(2)	26(1)
C(7)	3024(1)	9481(2)	4055(2)	26(1)
C(7A)	3363(1)	8395(2)	3899(2)	25(1)
C(8)	4025(1)	6364(2)	1778(2)	29(1)
C(9)	3417(1)	6060(2)	1358(3)	38(1)
C(10)	4726(1)	7013(2)	3616(2)	34(1)
C(11)	4535(1)	9514(2)	1453(2)	25(1)
C(12)	3523(1)	12737(2)	1941(2)	40(1)
O(1)	2926(1)	6728(2)	5227(2)	40(1)
O(2)	3737(1)	6387(1)	4062(2)	31(1)
O(3)	4437(1)	6108(2)	1099(2)	49(1)
O(4)	3852(1)	11586(1)	1840(2)	32(1)
O(5)	2564(1)	9435(2)	4870(2)	36(1)
O(6)	5000	10000	2245(2)	27(1)
C(13)	1898(1)	7766(2)	1403(2)	24(1)
C(15)	1148(1)	7784(2)	2950(2)	24(1)
C(15A)	1271(1)	6419(2)	2534(2)	22(1)
C(16)	1015(1)	5294(2)	2931(2)	22(1)
C(17)	1243(1)	4168(2)	2349(2)	24(1)
C(18)	1670(1)	4193(2)	1362(2)	26(1)

C(19)	1903(1)	5353(2)	946(2)	24(1)
C(19A)	1713(1)	6457(2)	1580(2)	23(1)
C(20)	532(1)	8199(2)	2504(2)	28(1)
C(21)	407(1)	8224(4)	1056(3)	63(1)
C(22)	1267(1)	8084(2)	4400(2)	30(1)
C(23)	503(1)	5207(2)	3850(2)	23(1)
C(24)	1185(1)	1889(2)	2256(2)	36(1)
O(7)	2290(1)	8207(1)	736(2)	32(1)
O(8)	1557(1)	8534(1)	2154(1)	27(1)
O(9)	181(1)	8488(2)	3322(2)	49(1)
O(10)	1008(1)	3081(1)	2822(2)	31(1)
O(11)	2290(1)	5470(1)	-48(2)	29(1)
O(12)	0	5000	3048(2)	24(1)

Table S3. Bond lengths [Å] and angles [°] for leptosphaerin J (**1**)

C(1)-O(1)	1.214(2)
C(1)-O(2)	1.360(2)
C(1)-C(7A)	1.447(3)
C(3)-O(2)	1.465(2)
C(3)-C(10)	1.516(2)
C(3)-C(3A)	1.519(2)
C(3)-C(8)	1.535(3)
C(3A)-C(4)	1.383(2)
C(3A)-C(7A)	1.393(2)
C(4)-C(5)	1.416(2)
C(4)-C(11)	1.499(2)
C(5)-O(4)	1.355(2)
C(5)-C(6)	1.390(3)
C(6)-C(7)	1.394(3)
C(6)-H(6)	0.9500
C(7)-O(5)	1.341(2)
C(7)-C(7A)	1.392(3)
C(8)-O(3)	1.199(3)
C(8)-C(9)	1.500(3)
C(9)-H(9A)	0.9800
C(9)-H(9B)	0.9800
C(9)-H(9C)	0.9800
C(10)-H(10A)	0.9800
C(10)-H(10B)	0.9800
C(10)-H(10C)	0.9800
C(11)-O(6)	1.430(2)
C(11)-H(11A)	0.9900
C(11)-H(11B)	0.9900
C(12)-O(4)	1.430(2)
C(12)-H(12A)	0.9800
C(12)-H(12B)	0.9800
C(12)-H(12C)	0.9800
O(5)-H(5)	0.90(3)
O(6)-C(11)#1	1.430(2)
C(13)-O(7)	1.216(2)
C(13)-O(8)	1.356(2)

C(13)-C(19A)	1.451(2)
C(15)-O(8)	1.466(2)
C(15)-C(22)	1.514(3)
C(15)-C(15A)	1.518(2)
C(15)-C(20)	1.551(3)
C(15A)-C(16)	1.379(2)
C(15A)-C(19A)	1.399(3)
C(16)-C(17)	1.420(2)
C(16)-C(23)	1.502(2)
C(17)-O(10)	1.349(2)
C(17)-C(18)	1.396(3)
C(18)-C(19)	1.395(3)
C(18)-H(18)	0.9500
C(19)-O(11)	1.344(2)
C(19)-C(19A)	1.392(3)
C(20)-O(9)	1.193(3)
C(20)-C(21)	1.482(3)
C(21)-H(21A)	0.9800
C(21)-H(21B)	0.9800
C(21)-H(21C)	0.9800
C(22)-H(22A)	0.9800
C(22)-H(22B)	0.9800
C(22)-H(22C)	0.9800
C(23)-O(12)	1.4291(19)
C(23)-H(23A)	0.9900
C(23)-H(23B)	0.9900
C(24)-O(10)	1.433(2)
C(24)-H(24A)	0.9800
C(24)-H(24B)	0.9800
C(24)-H(24C)	0.9800
O(11)-H(11)	0.90(3)
O(12)-C(23)#2	1.4291(19)

O(1)-C(1)-O(2)	120.55(18)
O(1)-C(1)-C(7A)	130.64(19)
O(2)-C(1)-C(7A)	108.80(16)
O(2)-C(3)-C(10)	108.34(16)
O(2)-C(3)-C(3A)	103.34(14)

C(10)-C(3)-C(3A)	114.83(15)
O(2)-C(3)-C(8)	105.32(14)
C(10)-C(3)-C(8)	113.11(16)
C(3A)-C(3)-C(8)	110.93(15)
C(4)-C(3A)-C(7A)	122.37(16)
C(4)-C(3A)-C(3)	129.93(16)
C(7A)-C(3A)-C(3)	107.69(15)
C(3A)-C(4)-C(5)	115.47(16)
C(3A)-C(4)-C(11)	124.92(16)
C(5)-C(4)-C(11)	119.51(16)
O(4)-C(5)-C(6)	122.76(16)
O(4)-C(5)-C(4)	114.33(16)
C(6)-C(5)-C(4)	122.91(16)
C(5)-C(6)-C(7)	120.08(16)
C(5)-C(6)-H(6)	120.0
C(7)-C(6)-H(6)	120.0
O(5)-C(7)-C(7A)	119.00(17)
O(5)-C(7)-C(6)	123.23(17)
C(7A)-C(7)-C(6)	117.77(17)
C(7)-C(7A)-C(3A)	121.37(17)
C(7)-C(7A)-C(1)	129.55(17)
C(3A)-C(7A)-C(1)	109.06(16)
O(3)-C(8)-C(9)	122.2(2)
O(3)-C(8)-C(3)	120.23(18)
C(9)-C(8)-C(3)	117.50(17)
C(8)-C(9)-H(9A)	109.5
C(8)-C(9)-H(9B)	109.5
H(9A)-C(9)-H(9B)	109.5
C(8)-C(9)-H(9C)	109.5
H(9A)-C(9)-H(9C)	109.5
H(9B)-C(9)-H(9C)	109.5
C(3)-C(10)-H(10A)	109.5
C(3)-C(10)-H(10B)	109.5
H(10A)-C(10)-H(10B)	109.5
C(3)-C(10)-H(10C)	109.5
H(10A)-C(10)-H(10C)	109.5
H(10B)-C(10)-H(10C)	109.5
O(6)-C(11)-C(4)	107.15(15)

O(6)-C(11)-H(11A)	110.3
C(4)-C(11)-H(11A)	110.3
O(6)-C(11)-H(11B)	110.3
C(4)-C(11)-H(11B)	110.3
H(11A)-C(11)-H(11B)	108.5
O(4)-C(12)-H(12A)	109.5
O(4)-C(12)-H(12B)	109.5
H(12A)-C(12)-H(12B)	109.5
O(4)-C(12)-H(12C)	109.5
H(12A)-C(12)-H(12C)	109.5
H(12B)-C(12)-H(12C)	109.5
C(1)-O(2)-C(3)	111.03(14)
C(5)-O(4)-C(12)	118.42(16)
C(7)-O(5)-H(5)	109(2)
C(11)-O(6)-C(11)#1	112.5(2)
O(7)-C(13)-O(8)	120.76(17)
O(7)-C(13)-C(19A)	130.35(18)
O(8)-C(13)-C(19A)	108.88(15)
O(8)-C(15)-C(22)	107.15(14)
O(8)-C(15)-C(15A)	103.68(14)
C(22)-C(15)-C(15A)	115.20(16)
O(8)-C(15)-C(20)	106.37(14)
C(22)-C(15)-C(20)	112.61(16)
C(15A)-C(15)-C(20)	110.96(15)
C(16)-C(15A)-C(19A)	122.30(17)
C(16)-C(15A)-C(15)	130.35(16)
C(19A)-C(15A)-C(15)	107.33(15)
C(15A)-C(16)-C(17)	115.74(16)
C(15A)-C(16)-C(23)	124.55(16)
C(17)-C(16)-C(23)	119.61(16)
O(10)-C(17)-C(18)	123.25(17)
O(10)-C(17)-C(16)	114.20(16)
C(18)-C(17)-C(16)	122.54(16)
C(19)-C(18)-C(17)	120.02(17)
C(19)-C(18)-H(18)	120.0
C(17)-C(18)-H(18)	120.0
O(11)-C(19)-C(19A)	118.19(16)
O(11)-C(19)-C(18)	123.90(17)

C(19A)-C(19)-C(18)	117.90(16)
C(19)-C(19A)-C(15A)	121.25(16)
C(19)-C(19A)-C(13)	129.74(17)
C(15A)-C(19A)-C(13)	109.00(15)
O(9)-C(20)-C(21)	122.5(2)
O(9)-C(20)-C(15)	119.70(18)
C(21)-C(20)-C(15)	117.82(18)
C(20)-C(21)-H(21A)	109.5
C(20)-C(21)-H(21B)	109.5
H(21A)-C(21)-H(21B)	109.5
C(20)-C(21)-H(21C)	109.5
H(21A)-C(21)-H(21C)	109.5
H(21B)-C(21)-H(21C)	109.5
C(15)-C(22)-H(22A)	109.5
C(15)-C(22)-H(22B)	109.5
H(22A)-C(22)-H(22B)	109.5
C(15)-C(22)-H(22C)	109.5
H(22A)-C(22)-H(22C)	109.5
H(22B)-C(22)-H(22C)	109.5
O(12)-C(23)-C(16)	107.60(14)
O(12)-C(23)-H(23A)	110.2
C(16)-C(23)-H(23A)	110.2
O(12)-C(23)-H(23B)	110.2
C(16)-C(23)-H(23B)	110.2
H(23A)-C(23)-H(23B)	108.5
O(10)-C(24)-H(24A)	109.5
O(10)-C(24)-H(24B)	109.5
H(24A)-C(24)-H(24B)	109.5
O(10)-C(24)-H(24C)	109.5
H(24A)-C(24)-H(24C)	109.5
H(24B)-C(24)-H(24C)	109.5
C(13)-O(8)-C(15)	110.95(14)
C(17)-O(10)-C(24)	119.02(15)
C(19)-O(11)-H(11)	107.8(18)
C(23)-O(12)-C(23)#2	111.41(19)

Symmetry transformations used to generate equivalent atoms:

#1 -x+1,-y+2,z #2 -x,-y+1,z

Table S4. Anisotropic displacement parameters (Å^{2}x 10^3) for leptosphaerin J (**1**) . The anisotropic displacement factor exponent takes the form: $-2\pi^2[h^2 a^{*2}U^{11} + ... + 2 h k a^* b^* U^{12}]$

	U^{11}	U^{22}	U^{33}	U^{23}	U^{13}	U^{12}
C(1)	31(1)	27(1)	30(1)	2(1)	2(1)	-4(1)
C(3)	23(1)	22(1)	31(1)	3(1)	0(1)	0(1)
C(3A)	19(1)	22(1)	24(1)	-1(1)	-2(1)	-2(1)
C(4)	19(1)	23(1)	23(1)	-1(1)	-2(1)	-3(1)
C(5)	25(1)	22(1)	26(1)	2(1)	-3(1)	-3(1)
C(6)	25(1)	24(1)	29(1)	-3(1)	-2(1)	3(1)
C(7)	21(1)	29(1)	30(1)	-3(1)	4(1)	-2(1)
C(7A)	23(1)	24(1)	27(1)	0(1)	1(1)	-4(1)
C(8)	31(1)	21(1)	35(1)	0(1)	1(1)	-1(1)
C(9)	36(1)	32(1)	46(1)	-4(1)	-9(1)	-3(1)
C(10)	28(1)	30(1)	45(1)	1(1)	-10(1)	4(1)
C(11)	20(1)	27(1)	27(1)	-1(1)	0(1)	-6(1)
C(12)	62(1)	24(1)	33(1)	6(1)	1(1)	8(1)
O(1)	42(1)	35(1)	44(1)	7(1)	15(1)	-8(1)
O(2)	35(1)	22(1)	35(1)	6(1)	5(1)	0(1)
O(3)	40(1)	59(1)	48(1)	-19(1)	10(1)	-3(1)
O(4)	38(1)	22(1)	38(1)	6(1)	4(1)	-1(1)
O(5)	29(1)	33(1)	46(1)	-1(1)	14(1)	1(1)
O(6)	20(1)	35(1)	26(1)	0	0	-7(1)
C(13)	24(1)	24(1)	25(1)	0(1)	1(1)	-1(1)
C(15)	25(1)	22(1)	24(1)	-1(1)	4(1)	-3(1)
C(15A)	20(1)	23(1)	21(1)	-1(1)	-1(1)	-1(1)
C(16)	22(1)	23(1)	22(1)	-1(1)	0(1)	-2(1)
C(17)	23(1)	23(1)	27(1)	0(1)	-3(1)	-2(1)
C(18)	25(1)	24(1)	29(1)	-5(1)	-1(1)	2(1)
C(19)	19(1)	28(1)	25(1)	-2(1)	0(1)	0(1)
C(19A)	22(1)	25(1)	22(1)	0(1)	1(1)	-2(1)
C(20)	28(1)	22(1)	33(1)	1(1)	2(1)	2(1)
C(21)	43(1)	109(3)	38(1)	-3(2)	-5(1)	28(2)
C(22)	34(1)	27(1)	28(1)	-4(1)	1(1)	-5(1)
C(23)	22(1)	24(1)	23(1)	1(1)	-2(1)	-4(1)
C(24)	42(1)	21(1)	44(1)	-4(1)	0(1)	-1(1)
O(7)	31(1)	29(1)	36(1)	2(1)	10(1)	-5(1)

O(8)	29(1)	22(1)	31(1)	0(1)	7(1)	-2(1)
O(9)	36(1)	69(1)	43(1)	-3(1)	8(1)	16(1)
O(10)	36(1)	19(1)	38(1)	-1(1)	6(1)	-3(1)
O(11)	28(1)	29(1)	32(1)	-4(1)	8(1)	0(1)
O(12)	20(1)	32(1)	21(1)	0	0	-2(1)

Table S5. Hydrogen coordinates (x 10^4) and isotropic displacement parameters (Å^{2}x 10 ^{3}) for leptosphaerin J(**1**).

	x	y	z	U(eq)
H(6)	2960	11327	3429	32
H(9A)	3196	6852	1264	57
H(9B)	3234	5520	2034	57
H(9C)	3424	5609	503	57
H(10A)	4743	7371	4518	51
H(10B)	4976	7511	3023	51
H(10C)	4859	6127	3635	51
H(11A)	4632	8657	1108	30
H(11B)	4460	10085	686	30
H(12A)	3705	13400	1395	60
H(12B)	3512	13015	2873	60
H(12C)	3128	12585	1624	60
H(5)	2369(13)	10180(30)	4810(30)	54
H(18)	1801	3420	974	31
H(21A)	-10	8105	913	95
H(21B)	620	7537	613	95
H(21C)	527	9047	684	95
H(22A)	1670	7873	4612	45
H(22B)	1007	7580	4966	45
H(22C)	1200	8993	4562	45
H(23A)	555	4494	4484	28
H(23B)	461	6007	4366	28
H(24A)	1094	1881	1302	53
H(24B)	978	1192	2700	53
H(24C)	1603	1778	2381	53
H(11)	2401(12)	4680(30)	-290(30)	44

Table S6. Torsion angles [°] for leptosphaerin J (**1**).

O(2)-C(3)-C(3A)-C(4)	179.58(17)
C(10)-C(3)-C(3A)-C(4)	61.8(3)
C(8)-C(3)-C(3A)-C(4)	-68.0(2)
O(2)-C(3)-C(3A)-C(7A)	-1.15(19)
C(10)-C(3)-C(3A)-C(7A)	-118.93(18)
C(8)-C(3)-C(3A)-C(7A)	111.26(17)
C(7A)-C(3A)-C(4)-C(5)	1.2(3)
C(3)-C(3A)-C(4)-C(5)	-179.66(17)
C(7A)-C(3A)-C(4)-C(11)	177.59(17)
C(3)-C(3A)-C(4)-C(11)	-3.2(3)
C(3A)-C(4)-C(5)-O(4)	177.79(15)
C(11)-C(4)-C(5)-O(4)	1.2(2)
C(3A)-C(4)-C(5)-C(6)	-1.9(3)
C(11)-C(4)-C(5)-C(6)	-178.54(17)
O(4)-C(5)-C(6)-C(7)	-178.20(18)
C(4)-C(5)-C(6)-C(7)	1.5(3)
C(5)-C(6)-C(7)-O(5)	179.80(18)
C(5)-C(6)-C(7)-C(7A)	-0.2(3)
O(5)-C(7)-C(7A)-C(3A)	179.49(17)
C(6)-C(7)-C(7A)-C(3A)	-0.5(3)
O(5)-C(7)-C(7A)-C(1)	0.9(3)
C(6)-C(7)-C(7A)-C(1)	-179.10(19)
C(4)-C(3A)-C(7A)-C(7)	0.0(3)
C(3)-C(3A)-C(7A)-C(7)	-179.35(17)
C(4)-C(3A)-C(7A)-C(1)	178.85(17)
C(3)-C(3A)-C(7A)-C(1)	-0.5(2)
O(1)-C(1)-C(7A)-C(7)	2.1(4)
O(2)-C(1)-C(7A)-C(7)	-179.2(2)
O(1)-C(1)-C(7A)-C(3A)	-176.7(2)
O(2)-C(1)-C(7A)-C(3A)	2.1(2)
O(2)-C(3)-C(8)-O(3)	-135.2(2)
C(10)-C(3)-C(8)-O(3)	-17.1(3)
C(3A)-C(3)-C(8)-O(3)	113.6(2)
O(2)-C(3)-C(8)-C(9)	46.5(2)

C(10)-C(3)-C(8)-C(9)	164.68(17)
C(3A)-C(3)-C(8)-C(9)	-64.6(2)
C(3A)-C(4)-C(11)-O(6)	-92.6(2)
C(5)-C(4)-C(11)-O(6)	83.72(19)
O(1)-C(1)-O(2)-C(3)	175.99(18)
C(7A)-C(1)-O(2)-C(3)	-2.9(2)
C(10)-C(3)-O(2)-C(1)	124.74(17)
C(3A)-C(3)-O(2)-C(1)	2.5(2)
C(8)-C(3)-O(2)-C(1)	-113.95(17)
C(6)-C(5)-O(4)-C(12)	-2.0(3)
C(4)-C(5)-O(4)-C(12)	178.33(17)
C(4)-C(11)-O(6)-C(11)#1	-161.51(16)
O(8)-C(15)-C(15A)-C(16)	-178.79(18)
C(22)-C(15)-C(15A)-C(16)	64.5(3)
C(20)-C(15)-C(15A)-C(16)	-65.0(3)
O(8)-C(15)-C(15A)-C(19A)	-0.34(19)
C(22)-C(15)-C(15A)-C(19A)	-117.07(17)
C(20)-C(15)-C(15A)-C(19A)	113.48(17)
C(19A)-C(15A)-C(16)-C(17)	2.5(3)
C(15)-C(15A)-C(16)-C(17)	-179.29(18)
C(19A)-C(15A)-C(16)-C(23)	-173.88(16)
C(15)-C(15A)-C(16)-C(23)	4.4(3)
C(15A)-C(16)-C(17)-O(10)	175.96(17)
C(23)-C(16)-C(17)-O(10)	-7.5(2)
C(15A)-C(16)-C(17)-C(18)	-4.7(3)
C(23)-C(16)-C(17)-C(18)	171.81(17)
O(10)-C(17)-C(18)-C(19)	-178.42(17)
C(16)-C(17)-C(18)-C(19)	2.3(3)
C(17)-C(18)-C(19)-O(11)	-176.07(17)
C(17)-C(18)-C(19)-C(19A)	2.4(3)
O(11)-C(19)-C(19A)-C(15A)	173.92(16)
C(18)-C(19)-C(19A)-C(15A)	-4.7(3)
O(11)-C(19)-C(19A)-C(13)	-4.9(3)
C(18)-C(19)-C(19A)-C(13)	176.53(19)
C(16)-C(15A)-C(19A)-C(19)	2.2(3)
C(15)-C(15A)-C(19A)-C(19)	-176.40(16)
C(16)-C(15A)-C(19A)-C(13)	-178.79(17)
C(15)-C(15A)-C(19A)-C(13)	2.6(2)

O(7)-C(13)-C(19A)-C(19)	-6.0(4)
O(8)-C(13)-C(19A)-C(19)	174.79(18)
O(7)-C(13)-C(19A)-C(15A)	175.1(2)
O(8)-C(13)-C(19A)-C(15A)	-4.1(2)
O(8)-C(15)-C(20)-O(9)	-130.1(2)
C(22)-C(15)-C(20)-O(9)	-13.0(3)
C(15A)-C(15)-C(20)-O(9)	117.8(2)
O(8)-C(15)-C(20)-C(21)	49.7(3)
C(22)-C(15)-C(20)-C(21)	166.8(2)
C(15A)-C(15)-C(20)-C(21)	-62.4(3)
C(15A)-C(16)-C(23)-O(12)	100.63(19)
C(17)-C(16)-C(23)-O(12)	-75.57(19)
O(7)-C(13)-O(8)-C(15)	-175.41(17)
C(19A)-C(13)-O(8)-C(15)	3.9(2)
C(22)-C(15)-O(8)-C(13)	120.00(17)
C(15A)-C(15)-O(8)-C(13)	-2.25(19)
C(20)-C(15)-O(8)-C(13)	-119.33(16)
C(18)-C(17)-O(10)-C(24)	-2.5(3)
C(16)-C(17)-O(10)-C(24)	176.82(17)
C(16)-C(23)-O(12)-C(23)#2	174.87(16)

Symmetry transformations used to generate equivalent atoms:

#1 -x+1,-y+2,z #2 -x,-y+1,z

Table S7. Hydrogen bonds for leptosphaerin J(**1**) [Å and °].

D-H...A	d(D-H)	d(H...A)	d(D...A)	<(DHA)
O(5)-H(5)...O(1)#3	0.90(3)	1.76(3)	2.660(2)	172(3)
O(11)-H(11)...O(7)#4	0.90(3)	1.76(3)	2.656(2)	173(3)

Symmetry transformations used to generate equivalent atoms:

#1 -x+1,-y+2,z #2 -x,-y+1,z #3 -x+1/2,y+1/2,-z+1

#4 -x+1/2,y-1/2,-z

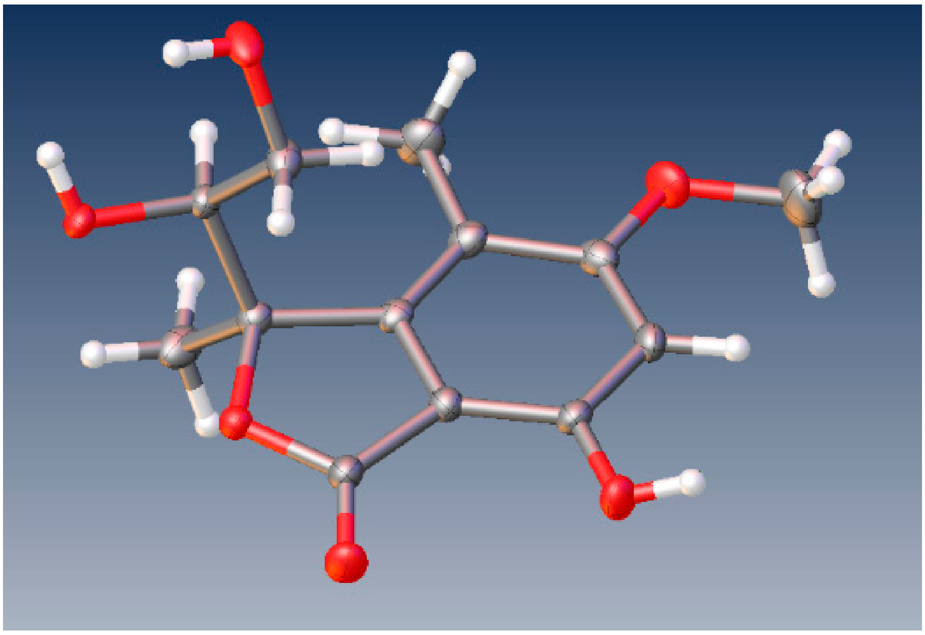

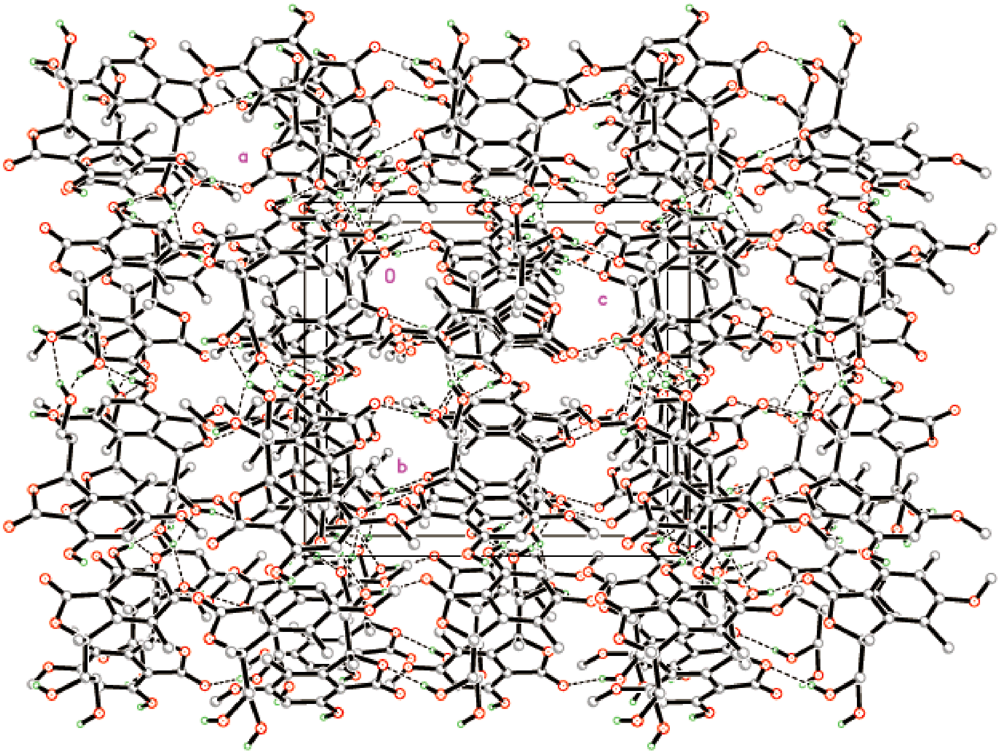
a
0
c
b

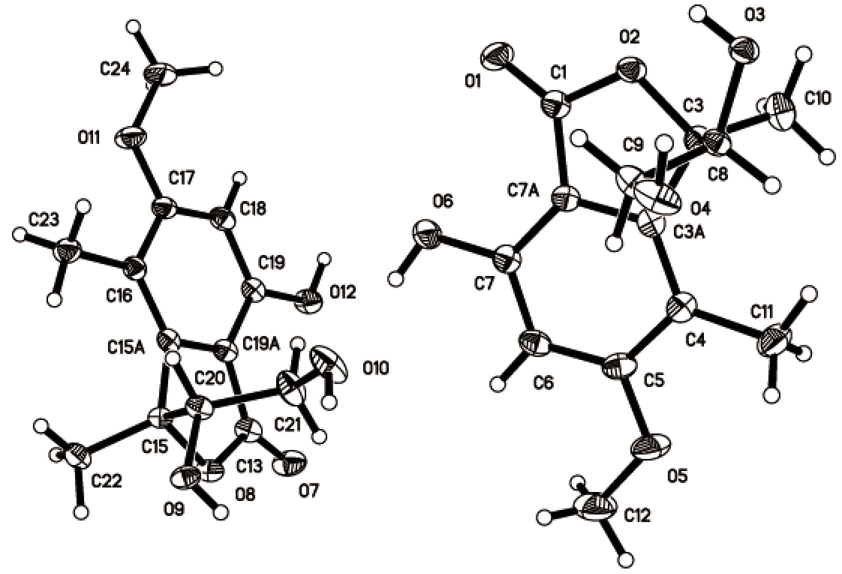

Figure S61. X-Ray structure of clearanol E (**7**)

Table S8. Crystal data and structure refinement for clearanol E (**7**)

Identification code	1606215	
Empirical formula	$C_{26}H_{26}O_{11}$	
Formula weight	514.47	
Temperature	150(2) K	
Wavelength	1.54184 Å	
Crystal system	Orthorhombic	
Space group	$P2_12_12$	
Unit cell dimensions	a = 23.08124(16) Å	α= 90°.
	b = 10.49438(8) Å	β= 90°.
	c = 10.03167(10) Å	γ = 90°.
Volume	2429.90(3) Å^3	
Z	4	
Density (calculated)	1.406 Mg/m^3	
Absorption coefficient	0.938 mm^{-1}	
F(000)	1080	
Crystal size	0.390 x 0.080 x 0.040 mm^3	
Theta range for data collection	3.830 to 74.081°.	
Index ranges	-28<=h<=28, -13<=k<=12, -11<=l<=12	
Reflections collected	23207	
Independent reflections	4841 [R(int) = 0.0236]	
Completeness to theta = 67.684°	99.9 %	

Absorption correction	Semi-empirical from equivalents
Max. and min. transmission	1.00000 and 0.69119
Refinement method	Full-matrix least-squares on F^2
Data / restraints / parameters	4841 / 0 / 347
Goodness-of-fit on F^2	1.031
Final R indices [I>2sigma(I)]	R1 = 0.0289, wR2 = 0.0758
R indices (all data)	R1 = 0.0297, wR2 = 0.0765
Absolute structure parameter	0.00(5)
Extinction coefficient	n/a
Largest diff. peak and hole	0.176 and -0.175 e.Å^{-3}

Table S9. Atomic coordinates (x 10^4) and equivalent isotropic displacement parameters ($Å^2$x 10^3) for clearanol E (**7**). U(eq) is defined as one third of the trace of the orthogonalized U^{ij} tensor.

	x	y	z	U(eq)
C(1)	3297(1)	7145(2)	4488(2)	30(1)
C(3)	4108(1)	7062(2)	3109(2)	25(1)
C(3A)	3844(1)	8387(2)	3058(2)	22(1)
C(4)	4011(1)	9447(2)	2333(2)	22(1)
C(5)	3667(1)	10550(2)	2524(2)	24(1)
C(6)	3183(1)	10570(2)	3348(2)	26(1)
C(7)	3024(1)	9481(2)	4055(2)	26(1)
C(7A)	3363(1)	8395(2)	3899(2)	25(1)
C(8)	4025(1)	6364(2)	1778(2)	29(1)
C(9)	3417(1)	6060(2)	1358(3)	38(1)
C(10)	4726(1)	7013(2)	3616(2)	34(1)
C(11)	4535(1)	9514(2)	1453(2)	25(1)
C(12)	3523(1)	12737(2)	1941(2)	40(1)
O(1)	2926(1)	6728(2)	5227(2)	40(1)
O(2)	3737(1)	6387(1)	4062(2)	31(1)
O(3)	4437(1)	6108(2)	1099(2)	49(1)
O(4)	3852(1)	11586(1)	1840(2)	32(1)
O(5)	2564(1)	9435(2)	4870(2)	36(1)
O(6)	5000	10000	2245(2)	27(1)
C(13)	1898(1)	7766(2)	1403(2)	24(1)
C(15)	1148(1)	7784(2)	2950(2)	24(1)
C(15A)	1271(1)	6419(2)	2534(2)	22(1)
C(16)	1015(1)	5294(2)	2931(2)	22(1)
C(17)	1243(1)	4168(2)	2349(2)	24(1)
C(18)	1670(1)	4193(2)	1362(2)	26(1)
C(19)	1903(1)	5353(2)	946(2)	24(1)
C(19A)	1713(1)	6457(2)	1580(2)	23(1)
C(20)	532(1)	8199(2)	2504(2)	28(1)
C(21)	407(1)	8224(4)	1056(3)	63(1)
C(22)	1267(1)	8084(2)	4400(2)	30(1)
C(23)	503(1)	5207(2)	3850(2)	23(1)
C(24)	1185(1)	1889(2)	2256(2)	36(1)
O(7)	2290(1)	8207(1)	736(2)	32(1)

O(8)	1557(1)	8534(1)	2154(1)	27(1)
O(9)	181(1)	8488(2)	3322(2)	49(1)
O(10)	1008(1)	3081(1)	2822(2)	31(1)
O(11)	2290(1)	5470(1)	-48(2)	29(1)
O(12)	0	5000	3048(2)	24(1)

Table S10. Bond lengths [Å] and angles [°] for clearanol E (**7**)

C(1)-O(1)	1.214(2)
C(1)-O(2)	1.360(2)
C(1)-C(7A)	1.447(3)
C(3)-O(2)	1.465(2)
C(3)-C(10)	1.516(2)
C(3)-C(3A)	1.519(2)
C(3)-C(8)	1.535(3)
C(3A)-C(4)	1.383(2)
C(3A)-C(7A)	1.393(2)
C(4)-C(5)	1.416(2)
C(4)-C(11)	1.499(2)
C(5)-O(4)	1.355(2)
C(5)-C(6)	1.390(3)
C(6)-C(7)	1.394(3)
C(6)-H(6)	0.9500
C(7)-O(5)	1.341(2)
C(7)-C(7A)	1.392(3)
C(8)-O(3)	1.199(3)
C(8)-C(9)	1.500(3)
C(9)-H(9A)	0.9800
C(9)-H(9B)	0.9800
C(9)-H(9C)	0.9800
C(10)-H(10A)	0.9800
C(10)-H(10B)	0.9800
C(10)-H(10C)	0.9800
C(11)-O(6)	1.430(2)
C(11)-H(11A)	0.9900
C(11)-H(11B)	0.9900
C(12)-O(4)	1.430(2)
C(12)-H(12A)	0.9800

C(12)-H(12B)	0.9800
C(12)-H(12C)	0.9800
O(5)-H(5)	0.90(3)
O(6)-C(11)#1	1.430(2)
C(13)-O(7)	1.216(2)
C(13)-O(8)	1.356(2)
C(13)-C(19A)	1.451(2)
C(15)-O(8)	1.466(2)
C(15)-C(22)	1.514(3)
C(15)-C(15A)	1.518(2)
C(15)-C(20)	1.551(3)
C(15A)-C(16)	1.379(2)
C(15A)-C(19A)	1.399(3)
C(16)-C(17)	1.420(2)
C(16)-C(23)	1.502(2)
C(17)-O(10)	1.349(2)
C(17)-C(18)	1.396(3)
C(18)-C(19)	1.395(3)
C(18)-H(18)	0.9500
C(19)-O(11)	1.344(2)
C(19)-C(19A)	1.392(3)
C(20)-O(9)	1.193(3)
C(20)-C(21)	1.482(3)
C(21)-H(21A)	0.9800
C(21)-H(21B)	0.9800
C(21)-H(21C)	0.9800
C(22)-H(22A)	0.9800
C(22)-H(22B)	0.9800
C(22)-H(22C)	0.9800
C(23)-O(12)	1.4291(19)
C(23)-H(23A)	0.9900
C(23)-H(23B)	0.9900
C(24)-O(10)	1.433(2)
C(24)-H(24A)	0.9800
C(24)-H(24B)	0.9800
C(24)-H(24C)	0.9800
O(11)-H(11)	0.90(3)
O(12)-C(23)#2	1.4291(19)

O(1)-C(1)-O(2)	120.55(18)
O(1)-C(1)-C(7A)	130.64(19)
O(2)-C(1)-C(7A)	108.80(16)
O(2)-C(3)-C(10)	108.34(16)
O(2)-C(3)-C(3A)	103.34(14)
C(10)-C(3)-C(3A)	114.83(15)
O(2)-C(3)-C(8)	105.32(14)
C(10)-C(3)-C(8)	113.11(16)
C(3A)-C(3)-C(8)	110.93(15)
C(4)-C(3A)-C(7A)	122.37(16)
C(4)-C(3A)-C(3)	129.93(16)
C(7A)-C(3A)-C(3)	107.69(15)
C(3A)-C(4)-C(5)	115.47(16)
C(3A)-C(4)-C(11)	124.92(16)
C(5)-C(4)-C(11)	119.51(16)
O(4)-C(5)-C(6)	122.76(16)
O(4)-C(5)-C(4)	114.33(16)
C(6)-C(5)-C(4)	122.91(16)
C(5)-C(6)-C(7)	120.08(16)
C(5)-C(6)-H(6)	120.0
C(7)-C(6)-H(6)	120.0
O(5)-C(7)-C(7A)	119.00(17)
O(5)-C(7)-C(6)	123.23(17)
C(7A)-C(7)-C(6)	117.77(17)
C(7)-C(7A)-C(3A)	121.37(17)
C(7)-C(7A)-C(1)	129.55(17)
C(3A)-C(7A)-C(1)	109.06(16)
O(3)-C(8)-C(9)	122.2(2)
O(3)-C(8)-C(3)	120.23(18)
C(9)-C(8)-C(3)	117.50(17)
C(8)-C(9)-H(9A)	109.5
C(8)-C(9)-H(9B)	109.5
H(9A)-C(9)-H(9B)	109.5
C(8)-C(9)-H(9C)	109.5
H(9A)-C(9)-H(9C)	109.5
H(9B)-C(9)-H(9C)	109.5
C(3)-C(10)-H(10A)	109.5

C(3)-C(10)-H(10B)	109.5
H(10A)-C(10)-H(10B)	109.5
C(3)-C(10)-H(10C)	109.5
H(10A)-C(10)-H(10C)	109.5
H(10B)-C(10)-H(10C)	109.5
O(6)-C(11)-C(4)	107.15(15)
O(6)-C(11)-H(11A)	110.3
C(4)-C(11)-H(11A)	110.3
O(6)-C(11)-H(11B)	110.3
C(4)-C(11)-H(11B)	110.3
H(11A)-C(11)-H(11B)	108.5
O(4)-C(12)-H(12A)	109.5
O(4)-C(12)-H(12B)	109.5
H(12A)-C(12)-H(12B)	109.5
O(4)-C(12)-H(12C)	109.5
H(12A)-C(12)-H(12C)	109.5
H(12B)-C(12)-H(12C)	109.5
C(1)-O(2)-C(3)	111.03(14)
C(5)-O(4)-C(12)	118.42(16)
C(7)-O(5)-H(5)	109(2)
C(11)-O(6)-C(11)#1	112.5(2)
O(7)-C(13)-O(8)	120.76(17)
O(7)-C(13)-C(19A)	130.35(18)
O(8)-C(13)-C(19A)	108.88(15)
O(8)-C(15)-C(22)	107.15(14)
O(8)-C(15)-C(15A)	103.68(14)
C(22)-C(15)-C(15A)	115.20(16)
O(8)-C(15)-C(20)	106.37(14)
C(22)-C(15)-C(20)	112.61(16)
C(15A)-C(15)-C(20)	110.96(15)
C(16)-C(15A)-C(19A)	122.30(17)
C(16)-C(15A)-C(15)	130.35(16)
C(19A)-C(15A)-C(15)	107.33(15)
C(15A)-C(16)-C(17)	115.74(16)
C(15A)-C(16)-C(23)	124.55(16)
C(17)-C(16)-C(23)	119.61(16)
O(10)-C(17)-C(18)	123.25(17)
O(10)-C(17)-C(16)	114.20(16)

C(18)-C(17)-C(16)	122.54(16)
C(19)-C(18)-C(17)	120.02(17)
C(19)-C(18)-H(18)	120.0
C(17)-C(18)-H(18)	120.0
O(11)-C(19)-C(19A)	118.19(16)
O(11)-C(19)-C(18)	123.90(17)
C(19A)-C(19)-C(18)	117.90(16)
C(19)-C(19A)-C(15A)	121.25(16)
C(19)-C(19A)-C(13)	129.74(17)
C(15A)-C(19A)-C(13)	109.00(15)
O(9)-C(20)-C(21)	122.5(2)
O(9)-C(20)-C(15)	119.70(18)
C(21)-C(20)-C(15)	117.82(18)
C(20)-C(21)-H(21A)	109.5
C(20)-C(21)-H(21B)	109.5
H(21A)-C(21)-H(21B)	109.5
C(20)-C(21)-H(21C)	109.5
H(21A)-C(21)-H(21C)	109.5
H(21B)-C(21)-H(21C)	109.5
C(15)-C(22)-H(22A)	109.5
C(15)-C(22)-H(22B)	109.5
H(22A)-C(22)-H(22B)	109.5
C(15)-C(22)-H(22C)	109.5
H(22A)-C(22)-H(22C)	109.5
H(22B)-C(22)-H(22C)	109.5
O(12)-C(23)-C(16)	107.60(14)
O(12)-C(23)-H(23A)	110.2
C(16)-C(23)-H(23A)	110.2
O(12)-C(23)-H(23B)	110.2
C(16)-C(23)-H(23B)	110.2
H(23A)-C(23)-H(23B)	108.5
O(10)-C(24)-H(24A)	109.5
O(10)-C(24)-H(24B)	109.5
H(24A)-C(24)-H(24B)	109.5
O(10)-C(24)-H(24C)	109.5
H(24A)-C(24)-H(24C)	109.5
H(24B)-C(24)-H(24C)	109.5
C(13)-O(8)-C(15)	110.95(14)

C(17)-O(10)-C(24)	119.02(15)
C(19)-O(11)-H(11)	107.8(18)
C(23)-O(12)-C(23)#2	111.41(19)

Symmetry transformations used to generate equivalent atoms:

#1 -x+1,-y+2,z #2 -x,-y+1,z

Table S11. Anisotropic displacement parameters (A^2 x 10^3) for clearanol E (**7**).The anisotropic displacement factor exponent takes the form: -2 pi^2 [h^2 a*^2 U11 + ... + 2 h k a* b* U12]

	U^{11}	U^{22}	U^{33}	U^{23}	U^{13}	U^{12}
C(1)	31(1)	27(1)	30(1)	2(1)	2(1)	-4(1)
C(3)	23(1)	22(1)	31(1)	3(1)	0(1)	0(1)
C(3A)	19(1)	22(1)	24(1)	-1(1)	-2(1)	-2(1)
C(4)	19(1)	23(1)	23(1)	-1(1)	-2(1)	-3(1)
C(5)	25(1)	22(1)	26(1)	2(1)	-3(1)	-3(1)
C(6)	25(1)	24(1)	29(1)	-3(1)	-2(1)	3(1)
C(7)	21(1)	29(1)	30(1)	-3(1)	4(1)	-2(1)
C(7A)	23(1)	24(1)	27(1)	0(1)	1(1)	-4(1)
C(8)	31(1)	21(1)	35(1)	0(1)	1(1)	-1(1)
C(9)	36(1)	32(1)	46(1)	-4(1)	-9(1)	-3(1)
C(10)	28(1)	30(1)	45(1)	1(1)	-10(1)	4(1)
C(11)	20(1)	27(1)	27(1)	-1(1)	0(1)	-6(1)
C(12)	62(1)	24(1)	33(1)	6(1)	1(1)	8(1)
O(1)	42(1)	35(1)	44(1)	7(1)	15(1)	-8(1)
O(2)	35(1)	22(1)	35(1)	6(1)	5(1)	0(1)
O(3)	40(1)	59(1)	48(1)	-19(1)	10(1)	-3(1)
O(4)	38(1)	22(1)	38(1)	6(1)	4(1)	-1(1)
O(5)	29(1)	33(1)	46(1)	-1(1)	14(1)	1(1)
O(6)	20(1)	35(1)	26(1)	0	0	-7(1)
C(13)	24(1)	24(1)	25(1)	0(1)	1(1)	-1(1)
C(15)	25(1)	22(1)	24(1)	-1(1)	4(1)	-3(1)
C(15A)	20(1)	23(1)	21(1)	-1(1)	-1(1)	-1(1)
C(16)	22(1)	23(1)	22(1)	-1(1)	0(1)	-2(1)
C(17)	23(1)	23(1)	27(1)	0(1)	-3(1)	-2(1)
C(18)	25(1)	24(1)	29(1)	-5(1)	-1(1)	2(1)
C(19)	19(1)	28(1)	25(1)	-2(1)	0(1)	0(1)
C(19A)	22(1)	25(1)	22(1)	0(1)	1(1)	-2(1)

C(20)	28(1)	22(1)	33(1)	1(1)	2(1)	2(1)
C(21)	43(1)	109(3)	38(1)	-3(2)	-5(1)	28(2)
C(22)	34(1)	27(1)	28(1)	-4(1)	1(1)	-5(1)
C(23)	22(1)	24(1)	23(1)	1(1)	-2(1)	-4(1)
C(24)	42(1)	21(1)	44(1)	-4(1)	0(1)	-1(1)
O(7)	31(1)	29(1)	36(1)	2(1)	10(1)	-5(1)
O(8)	29(1)	22(1)	31(1)	0(1)	7(1)	-2(1)
O(9)	36(1)	69(1)	43(1)	-3(1)	8(1)	16(1)
O(10)	36(1)	19(1)	38(1)	-1(1)	6(1)	-3(1)
O(11)	28(1)	29(1)	32(1)	-4(1)	8(1)	0(1)
O(12)	20(1)	32(1)	21(1)	0	0	-2(1)

Table S12. Hydrogen coordinates (x 10^4) and isotropic displacement parameters (A^2 x 10^3) for clearanol E (**7**)

	x	y	z	U(eq)
H(6)	2960	11327	3429	32
H(9A)	3196	6852	1264	57
H(9B)	3234	5520	2034	57
H(9C)	3424	5609	503	57
H(10A)	4743	7371	4518	51
H(10B)	4976	7511	3023	51
H(10C)	4859	6127	3635	51
H(11A)	4632	8657	1108	30
H(11B)	4460	10085	686	30
H(12A)	3705	13400	1395	60
H(12B)	3512	13015	2873	60
H(12C)	3128	12585	1624	60
H(5)	2369(13)	10180(30)	4810(30)	54
H(18)	1801	3420	974	31
H(21A)	-10	8105	913	95
H(21B)	620	7537	613	95
H(21C)	527	9047	684	95
H(22A)	1670	7873	4612	45
H(22B)	1007	7580	4966	45

H(22C)	1200	8993	4562	45
H(23A)	555	4494	4484	28
H(23B)	461	6007	4366	28
H(24A)	1094	1881	1302	53
H(24B)	978	1192	2700	53
H(24C)	1603	1778	2381	53
H(11)	2401(12)	4680(30)	-290(30)	44

Table S13. Torsion angles [°] for clearanol E (**7**)

O(2)-C(3)-C(3A)-C(4)	179.58(17)
C(10)-C(3)-C(3A)-C(4)	61.8(3)
C(8)-C(3)-C(3A)-C(4)	-68.0(2)
O(2)-C(3)-C(3A)-C(7A)	-1.15(19)
C(10)-C(3)-C(3A)-C(7A)	-118.93(18)
C(8)-C(3)-C(3A)-C(7A)	111.26(17)
C(7A)-C(3A)-C(4)-C(5)	1.2(3)
C(3)-C(3A)-C(4)-C(5)	-179.66(17)
C(7A)-C(3A)-C(4)-C(11)	177.59(17)
C(3)-C(3A)-C(4)-C(11)	-3.2(3)
C(3A)-C(4)-C(5)-O(4)	177.79(15)
C(11)-C(4)-C(5)-O(4)	1.2(2)
C(3A)-C(4)-C(5)-C(6)	-1.9(3)
C(11)-C(4)-C(5)-C(6)	-178.54(17)
O(4)-C(5)-C(6)-C(7)	-178.20(18)
C(4)-C(5)-C(6)-C(7)	1.5(3)
C(5)-C(6)-C(7)-O(5)	179.80(18)
C(5)-C(6)-C(7)-C(7A)	-0.2(3)
O(5)-C(7)-C(7A)-C(3A)	179.49(17)
C(6)-C(7)-C(7A)-C(3A)	-0.5(3)
O(5)-C(7)-C(7A)-C(1)	0.9(3)
C(6)-C(7)-C(7A)-C(1)	-179.10(19)
C(4)-C(3A)-C(7A)-C(7)	0.0(3)
C(3)-C(3A)-C(7A)-C(7)	-179.35(17)
C(4)-C(3A)-C(7A)-C(1)	178.85(17)
C(3)-C(3A)-C(7A)-C(1)	-0.5(2)
O(1)-C(1)-C(7A)-C(7)	2.1(4)

O(2)-C(1)-C(7A)-C(7)	-179.2(2)
O(1)-C(1)-C(7A)-C(3A)	-176.7(2)
O(2)-C(1)-C(7A)-C(3A)	2.1(2)
O(2)-C(3)-C(8)-O(3)	-135.2(2)
C(10)-C(3)-C(8)-O(3)	-17.1(3)
C(3A)-C(3)-C(8)-O(3)	113.6(2)
O(2)-C(3)-C(8)-C(9)	46.5(2)
C(10)-C(3)-C(8)-C(9)	164.68(17)
C(3A)-C(3)-C(8)-C(9)	-64.6(2)
C(3A)-C(4)-C(11)-O(6)	-92.6(2)
C(5)-C(4)-C(11)-O(6)	83.72(19)
O(1)-C(1)-O(2)-C(3)	175.99(18)
C(7A)-C(1)-O(2)-C(3)	-2.9(2)
C(10)-C(3)-O(2)-C(1)	124.74(17)
C(3A)-C(3)-O(2)-C(1)	2.5(2)
C(8)-C(3)-O(2)-C(1)	-113.95(17)
C(6)-C(5)-O(4)-C(12)	-2.0(3)
C(4)-C(5)-O(4)-C(12)	178.33(17)
C(4)-C(11)-O(6)-C(11)#1	-161.51(16)
O(8)-C(15)-C(15A)-C(16)	-178.79(18)
C(22)-C(15)-C(15A)-C(16)	64.5(3)
C(20)-C(15)-C(15A)-C(16)	-65.0(3)
O(8)-C(15)-C(15A)-C(19A)	-0.34(19)
C(22)-C(15)-C(15A)-C(19A)	-117.07(17)
C(20)-C(15)-C(15A)-C(19A)	113.48(17)
C(19A)-C(15A)-C(16)-C(17)	2.5(3)
C(15)-C(15A)-C(16)-C(17)	-179.29(18)
C(19A)-C(15A)-C(16)-C(23)	-173.88(16)
C(15)-C(15A)-C(16)-C(23)	4.4(3)
C(15A)-C(16)-C(17)-O(10)	175.96(17)
C(23)-C(16)-C(17)-O(10)	-7.5(2)
C(15A)-C(16)-C(17)-C(18)	-4.7(3)
C(23)-C(16)-C(17)-C(18)	171.81(17)
O(10)-C(17)-C(18)-C(19)	-178.42(17)
C(16)-C(17)-C(18)-C(19)	2.3(3)
C(17)-C(18)-C(19)-O(11)	-176.07(17)
C(17)-C(18)-C(19)-C(19A)	2.4(3)
O(11)-C(19)-C(19A)-C(15A)	173.92(16)

C(18)-C(19)-C(19A)-C(15A)	-4.7(3)
O(11)-C(19)-C(19A)-C(13)	-4.9(3)
C(18)-C(19)-C(19A)-C(13)	176.53(19)
C(16)-C(15A)-C(19A)-C(19)	2.2(3)
C(15)-C(15A)-C(19A)-C(19)	-176.40(16)
C(16)-C(15A)-C(19A)-C(13)	-178.79(17)
C(15)-C(15A)-C(19A)-C(13)	2.6(2)
O(7)-C(13)-C(19A)-C(19)	-6.0(4)
O(8)-C(13)-C(19A)-C(19)	174.79(18)
O(7)-C(13)-C(19A)-C(15A)	175.1(2)
O(8)-C(13)-C(19A)-C(15A)	-4.1(2)
O(8)-C(15)-C(20)-O(9)	-130.1(2)
C(22)-C(15)-C(20)-O(9)	-13.0(3)
C(15A)-C(15)-C(20)-O(9)	117.8(2)
O(8)-C(15)-C(20)-C(21)	49.7(3)
C(22)-C(15)-C(20)-C(21)	166.8(2)
C(15A)-C(15)-C(20)-C(21)	-62.4(3)
C(15A)-C(16)-C(23)-O(12)	100.63(19)
C(17)-C(16)-C(23)-O(12)	-75.57(19)
O(7)-C(13)-O(8)-C(15)	-175.41(17)
C(19A)-C(13)-O(8)-C(15)	3.9(2)
C(22)-C(15)-O(8)-C(13)	120.00(17)
C(15A)-C(15)-O(8)-C(13)	-2.25(19)
C(20)-C(15)-O(8)-C(13)	-119.33(16)
C(18)-C(17)-O(10)-C(24)	-2.5(3)
C(16)-C(17)-O(10)-C(24)	176.82(17)
C(16)-C(23)-O(12)-C(23)#2	174.87(16)

Symmetry transformations used to generate equivalent atoms:

#1 -x+1,-y+2,z #2 -x,-y+1,z

Table S14. Hydrogen bonds for clearanol E (**7**) [Å and °].

D-H...A	d(D-H)	d(H...A)	d(D...A)	<(DHA)
O(5)-H(5)...O(1)#3	0.90(3)	1.76(3)	2.660(2)	172(3)
O(11)-H(11)...O(7)#4	0.90(3)	1.76(3)	2.656(2)	173(3)

Symmetry transformations used to generate equivalent atoms:

#1 -x+1,-y+2,z   #2 -x,-y+1,z   #3 -x+1/2,y+1/2,-z+1

#4 -x+1/2,y-1/2,-z

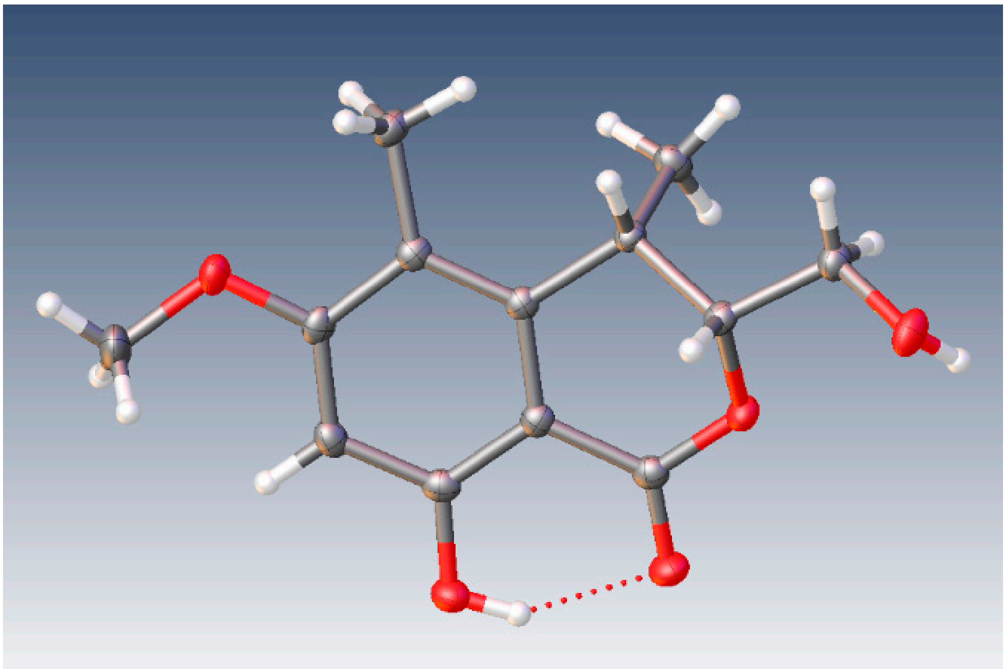

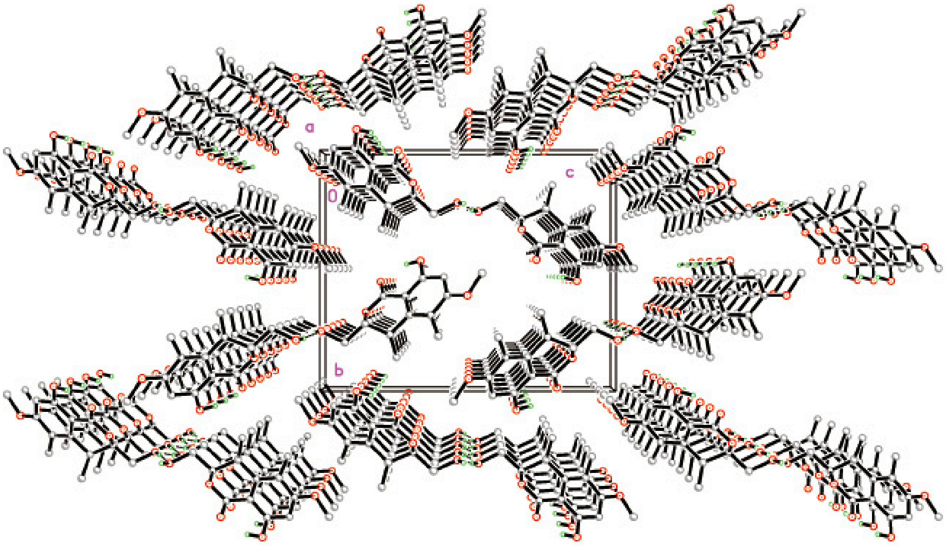

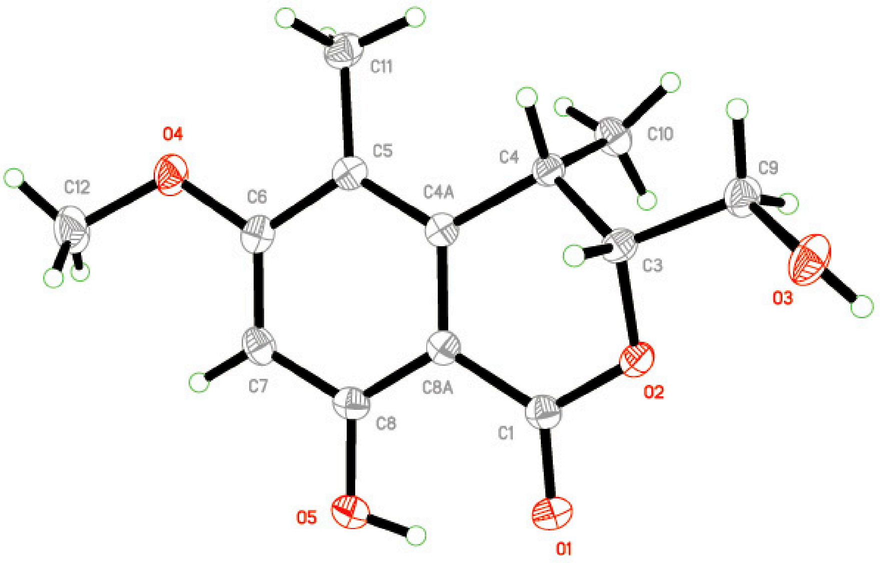

Figure S62. X-Ray structure of clearanol I (**9**)

Table S15. Crystal data and structure refinement for clearanol I (**9**)

Identification code	1606225	
Empirical formula	$C_{13}H_{16}O_5$	
Formula weight	252.26	
Temperature	150(2) K	
Wavelength	1.54184 Å	
Crystal system	Orthorhombic	
Space group	$P2_12_12_1$	
Unit cell dimensions	a = 4.91205(6) Å	α= 90°.
	b = 13.99641(14) Å	β= 90°.
	c = 17.18834(19) Å	γ = 90°.
Volume	1181.72(2) Å^3	
Z	4	
Density (calculated)	1.418 Mg/m^3	
Absorption coefficient	0.914 mm^{-1}	
F(000)	536	
Crystal size	0.360 x 0.320 x 0.240 mm^3	
Theta range for data collection	4.073 to 70.583°.	
Index ranges	-5<=h<=5, -16<=k<=16, -20<=l<=20	
Reflections collected	8470	
Independent reflections	2216 [R(int) = 0.0181]	
Completeness to theta = 67.684°	99.8 %	

Absorption correction	Semi-empirical from equivalents
Max. and min. transmission	1.00000 and 0.79385
Refinement method	Full-matrix least-squares on F^2
Data / restraints / parameters	2216 / 0 / 174
Goodness-of-fit on F^2	1.059
Final R indices [I>2sigma(I)]	R1 = 0.0256, wR2 = 0.0656
R indices (all data)	R1 = 0.0259, wR2 = 0.0658
Absolute structure parameter	-0.03(5)
Extinction coefficient	n/a
Largest diff. peak and hole	0.127 and -0.156 e.$Å^{-3}$

Table S16. Atomic coordinates (x 10^4) and equivalent isotropic displacement parameters (A^2 x 10^3) for clearanol I (**9**). U(eq) is defined as one third of the trace of the orthogonalized Uij tensor.

	x	y	z	U(eq)
C(1)	1572(3)	5986(1)	2284(1)	18(1)
C(3)	4842(3)	7056(1)	1735(1)	17(1)
C(4)	5504(3)	7477(1)	2531(1)	16(1)
C(4A)	5122(3)	6713(1)	3148(1)	15(1)
C(5)	6579(3)	6723(1)	3838(1)	17(1)
C(6)	6190(4)	5948(1)	4356(1)	18(1)
C(7)	4234(4)	5252(1)	4232(1)	20(1)
C(8)	2674(3)	5282(1)	3558(1)	18(1)
C(8A)	3180(3)	5990(1)	2994(1)	17(1)
C(9)	4871(4)	7790(1)	1090(1)	21(1)
C(10)	3871(4)	8382(1)	2722(1)	21(1)
C(11)	8548(4)	7498(1)	4070(1)	22(1)
C(12)	7992(5)	5094(1)	5441(1)	34(1)
O(1)	-354(3)	5440(1)	2170(1)	24(1)
O(2)	2123(2)	6628(1)	1723(1)	20(1)
O(3)	4808(3)	7356(1)	338(1)	25(1)
O(4)	7880(3)	5946(1)	4982(1)	24(1)
O(5)	743(3)	4606(1)	3465(1)	23(1)

Table S17. Bond lengths[Å] and angles [°] for clearanol I (**9**).

C(1)-O(1)	1.232(2)
C(1)-O(2)	1.3446(19)
C(1)-C(8A)	1.454(2)
C(3)-O(2)	1.4644(19)
C(3)-C(9)	1.511(2)
C(3)-C(4)	1.525(2)
C(3)-H(3A)	1.0000
C(4)-C(4A)	1.517(2)
C(4)-C(10)	1.535(2)
C(4)-H(4)	1.0000
C(4A)-C(5)	1.385(2)

C(4A)-C(8A)	1.415(2)
C(5)-C(6)	1.415(2)
C(5)-C(11)	1.507(2)
C(6)-O(4)	1.359(2)
C(6)-C(7)	1.385(2)
C(7)-C(8)	1.390(2)
C(7)-H(7)	0.9500
C(8)-O(5)	1.349(2)
C(8)-C(8A)	1.409(2)
C(9)-O(3)	1.4277(19)
C(9)-H(9A)	0.9900
C(9)-H(9B)	0.9900
C(10)-H(10A)	0.9800
C(10)-H(10B)	0.9800
C(10)-H(10C)	0.9800
C(11)-H(11A)	0.9800
C(11)-H(11B)	0.9800
C(11)-H(11C)	0.9800
C(12)-O(4)	1.432(2)
C(12)-H(12A)	0.9800
C(12)-H(12B)	0.9800
C(12)-H(12C)	0.9800
O(3)-H(3)	0.83(3)
O(5)-H(5)	0.88(3)
O(1)-C(1)-O(2)	117.05(14)
O(1)-C(1)-C(8A)	123.56(14)
O(2)-C(1)-C(8A)	119.35(14)
O(2)-C(3)-C(9)	106.04(13)
O(2)-C(3)-C(4)	111.43(12)
C(9)-C(3)-C(4)	113.24(12)
O(2)-C(3)-H(3A)	108.7
C(9)-C(3)-H(3A)	108.7
C(4)-C(3)-H(3A)	108.7
C(4A)-C(4)-C(3)	109.21(12)
C(4A)-C(4)-C(10)	111.58(13)
C(3)-C(4)-C(10)	113.51(13)
C(4A)-C(4)-H(4)	107.4

C(3)-C(4)-H(4)	107.4
C(10)-C(4)-H(4)	107.4
C(5)-C(4A)-C(8A)	121.05(14)
C(5)-C(4A)-C(4)	121.83(14)
C(8A)-C(4A)-C(4)	117.12(13)
C(4A)-C(5)-C(6)	117.43(14)
C(4A)-C(5)-C(11)	124.36(14)
C(6)-C(5)-C(11)	118.20(14)
O(4)-C(6)-C(7)	122.95(14)
O(4)-C(6)-C(5)	114.61(14)
C(7)-C(6)-C(5)	122.44(15)
C(6)-C(7)-C(8)	119.29(15)
C(6)-C(7)-H(7)	120.4
C(8)-C(7)-H(7)	120.4
O(5)-C(8)-C(7)	117.78(14)
O(5)-C(8)-C(8A)	122.35(15)
C(7)-C(8)-C(8A)	119.86(15)
C(8)-C(8A)-C(4A)	119.51(14)
C(8)-C(8A)-C(1)	118.67(14)
C(4A)-C(8A)-C(1)	121.73(14)
O(3)-C(9)-C(3)	112.04(13)
O(3)-C(9)-H(9A)	109.2
C(3)-C(9)-H(9A)	109.2
O(3)-C(9)-H(9B)	109.2
C(3)-C(9)-H(9B)	109.2
H(9A)-C(9)-H(9B)	107.9
C(4)-C(10)-H(10A)	109.5
C(4)-C(10)-H(10B)	109.5
H(10A)-C(10)-H(10B)	109.5
C(4)-C(10)-H(10C)	109.5
H(10A)-C(10)-H(10C)	109.5
H(10B)-C(10)-H(10C)	109.5
C(5)-C(11)-H(11A)	109.5
C(5)-C(11)-H(11B)	109.5
H(11A)-C(11)-H(11B)	109.5
C(5)-C(11)-H(11C)	109.5
H(11A)-C(11)-H(11C)	109.5
H(11B)-C(11)-H(11C)	109.5

O(4)-C(12)-H(12A)	109.5
O(4)-C(12)-H(12B)	109.5
H(12A)-C(12)-H(12B)	109.5
O(4)-C(12)-H(12C)	109.5
H(12A)-C(12)-H(12C)	109.5
H(12B)-C(12)-H(12C)	109.5
C(1)-O(2)-C(3)	116.54(12)
C(9)-O(3)-H(3)	110.7(19)
C(6)-O(4)-C(12)	117.46(14)
C(8)-O(5)-H(5)	105.2(17)

Symmetry transformations used to generate equivalent atoms:

Table S18. Anisotropic displacement parameters (A^2 x 10^3) for clearanol I (**9**). The anisotropic displacement factor exponent takes the form: -2 pi^2 [h^2 a*^2 U11 + ... + 2 h k a* b* U12]

	U^{11}	U^{22}	U^{33}	U^{23}	U^{13}	U^{12}
C(1)	17(1)	16(1)	21(1)	-2(1)	1(1)	2(1)
C(3)	14(1)	19(1)	18(1)	-1(1)	0(1)	1(1)
C(4)	15(1)	18(1)	16(1)	0(1)	-1(1)	-1(1)
C(4A)	14(1)	16(1)	15(1)	-2(1)	2(1)	2(1)
C(5)	15(1)	19(1)	16(1)	-2(1)	2(1)	1(1)
C(6)	20(1)	22(1)	13(1)	-3(1)	1(1)	4(1)
C(7)	24(1)	18(1)	17(1)	2(1)	3(1)	2(1)
C(8)	16(1)	16(1)	22(1)	-3(1)	3(1)	1(1)
C(8A)	17(1)	17(1)	17(1)	-1(1)	1(1)	2(1)
C(9)	22(1)	24(1)	16(1)	1(1)	0(1)	-1(1)
C(10)	26(1)	18(1)	21(1)	0(1)	3(1)	2(1)
C(11)	22(1)	26(1)	17(1)	-2(1)	-1(1)	-3(1)
C(12)	50(1)	29(1)	22(1)	6(1)	-11(1)	1(1)
O(1)	21(1)	23(1)	27(1)	0(1)	-6(1)	-5(1)
O(2)	18(1)	23(1)	18(1)	2(1)	-5(1)	-4(1)
O(3)	22(1)	38(1)	14(1)	-1(1)	-1(1)	1(1)
O(4)	31(1)	24(1)	16(1)	0(1)	-6(1)	1(1)
O(5)	23(1)	19(1)	28(1)	3(1)	-2(1)	-4(1)

Table S19. Hydrogen coordinates (x 10^4) and isotropic displacement parameters (A^2 x 10^3) for clearanol I (**9**).

	x	y	z	U(eq)
H(3A)	6212	6550	1612	20
H(4)	7474	7656	2529	20
H(7)	3961	4759	4604	24
H(9A)	3275	8216	1147	25
H(9B)	6532	8186	1136	25
H(10A)	1937	8269	2617	32
H(10B)	4520	8912	2399	32

H(10C)	4113	8544	3272	32
H(11A)	8553	8000	3671	32
H(11B)	10380	7228	4118	32
H(11C)	7989	7772	4570	32
H(12A)	8244	4540	5099	51
H(12B)	6289	5022	5732	51
H(12C)	9521	5134	5806	51
H(3)	3280(60)	7407(19)	141(15)	44(7)
H(5)	-40(60)	4740(18)	3015(16)	52(7)

Table S20. Torsion angles [°] for clearanol I (**9**).

O(2)-C(3)-C(4)-C(4A)	54.41(16)
C(9)-C(3)-C(4)-C(4A)	173.85(14)
O(2)-C(3)-C(4)-C(10)	-70.78(16)
C(9)-C(3)-C(4)-C(10)	48.67(18)
C(3)-C(4)-C(4A)-C(5)	152.08(14)
C(10)-C(4)-C(4A)-C(5)	-81.62(18)
C(3)-C(4)-C(4A)-C(8A)	-28.66(19)
C(10)-C(4)-C(4A)-C(8A)	97.64(16)
C(8A)-C(4A)-C(5)-C(6)	4.3(2)
C(4)-C(4A)-C(5)-C(6)	-176.42(14)
C(8A)-C(4A)-C(5)-C(11)	-175.91(15)
C(4)-C(4A)-C(5)-C(11)	3.3(2)
C(4A)-C(5)-C(6)-O(4)	173.39(14)
C(11)-C(5)-C(6)-O(4)	-6.4(2)
C(4A)-C(5)-C(6)-C(7)	-6.8(2)
C(11)-C(5)-C(6)-C(7)	173.41(15)
O(4)-C(6)-C(7)-C(8)	-177.01(15)
C(5)-C(6)-C(7)-C(8)	3.2(2)
C(6)-C(7)-C(8)-O(5)	-178.50(15)
C(6)-C(7)-C(8)-C(8A)	2.9(2)
O(5)-C(8)-C(8A)-C(4A)	176.23(14)
C(7)-C(8)-C(8A)-C(4A)	-5.2(2)
O(5)-C(8)-C(8A)-C(1)	-0.4(2)
C(7)-C(8)-C(8A)-C(1)	178.13(14)
C(5)-C(4A)-C(8A)-C(8)	1.5(2)

C(4)-C(4A)-C(8A)-C(8)	-177.78(13)
C(5)-C(4A)-C(8A)-C(1)	178.02(14)
C(4)-C(4A)-C(8A)-C(1)	-1.2(2)
O(1)-C(1)-C(8A)-C(8)	5.6(2)
O(2)-C(1)-C(8A)-C(8)	-176.68(14)
O(1)-C(1)-C(8A)-C(4A)	-170.98(15)
O(2)-C(1)-C(8A)-C(4A)	6.8(2)
O(2)-C(3)-C(9)-O(3)	-69.61(17)
C(4)-C(3)-C(9)-O(3)	167.90(14)
O(1)-C(1)-O(2)-C(3)	-161.10(13)
C(8A)-C(1)-O(2)-C(3)	21.02(19)
C(9)-C(3)-O(2)-C(1)	-176.20(13)
C(4)-C(3)-O(2)-C(1)	-52.56(16)
C(7)-C(6)-O(4)-C(12)	12.7(2)
C(5)-C(6)-O(4)-C(12)	-167.50(16)

Symmetry transformations used to generate equivalent atoms:

Table S21. Hydrogen bonds for clearanol I (**9**) [Å and °].

D-H...A	d(D-H)	d(H...A)	d(D...A)	<(DHA)
O(3)-H(3)...O(3)#1	0.83(3)	1.92(3)	2.7471(10)	175(3)
O(5)-H(5)...O(1)	0.88(3)	1.76(3)	2.5710(17)	151(3)

Symmetry transformations used to generate equivalent atoms:

#1 x-1/2,-y+3/2,-z

The physicochemical data of the known compounds

(*R*)-3-acetyl-7-hydroxy-5-methoxy-3,4-dimethylisobenzofuran-1(3*H*)-one (**5**): pale yellow oil; $[\alpha]_D^{25} = + 108.4°$ (*c* 0.15, MeOH) ; ^{1}H NMR (500 MHz, $CDCl_3$): δ_H 6.48 (1H, s, H-6), 3.87 (3H, s, H-12), 2.05 (3H, s, H-9), 2.03 (3H, s, H-11), 1.77 (3H, s, H-10). ^{13}C NMR (125 MHz, $CDCl_3$): δ_C 202.9 (qC, C-8), 171.6 (qC, C-1), 165.9 (qC, C-5), 156.7 (qC, C-7),146.3 (qC, C-3a), 114.5 (qC, C-4), 102.3 (qC, C-7a), 99.2 (CH, C-6), 91.6 (qC, C-3), 56.5 (CH_3, C-12), 24.2 (CH_3, C-9), 20.2 (CH_3, C-10), 10.1 (CH_3, C-11).

(3*R*,3^1*S*)-7-hydroxy-3-(1-hydroxyethyl)-5-methoxy-3,4-dimethylisobenzofuran-1(3*H*)-one (**6**) : pale yellow oil; LRESIMS $[M + H]^+$ *m/z* 253.1; $[2M + H]^+$ *m/z* 505.2; $[M - H]^-$ *m/z* 252.1; $[\alpha]_D^{25} = - 35.4°$ (*c* 0.68, MeOH) ; ^{1}H NMR (500 MHz, CD_3OD): δ_H 6.14 (1H, s, H-6), 3.92(1H, q, *J* = 6.5 Hz , H-9), 3.50 (3H, s, H-12), 0.50 (3H, *J* = 6.5 Hz , H-9), 1.72 (3H, s, H-11), 1.37 (3H, s, H-10). ^{13}C NMR (125 MHz, CD_3OD): δ_C 171.6 (qC, C-1), 166.0 (qC, C-5), 157.6 (qC, C-7),152.3 (qC, C-3a), 112.4 (qC, C-4), 104.3 (qC, C-7a), 99.4 (CH, C-6), 91.3 (qC, C-3), 70.7 (CH, C-8), 56.7 (CH_3, C-12), 17.2 (CH_3, C-9), 21.4 (CH_3, C-10), 11.3 (CH_3, C-11).

Clearanol E (**7**): white crystalline solid; LRESIMS $[M + H]^+$ *m/z* 269.1; $[M + Na]^+$ *m/z* 291.1; $[2M + Na]^+$ *m/z* 559.3; $[M - H]^-$ *m/z* 267.3; $[2M - H]^-$ *m/z* 535.2; $[\alpha]_D^{25} = -$ 20.9° (*c* 0.34, MeOH) ; ^{1}H NMR (700 MHz, $CDCl_3$): δ_H 6.43 (1H, s, H-6), 4.14(1H, dd, *J* = 3.5, 7.7 Hz , H-8), 3.37 (1H, dd, *J* = 11.9, 7.7 Hz , H-9a), 3.14 (1H, dd, *J* = 11.9, 3.5 Hz , H-9b), 3.87 (3H, s, H-12), 2.13 (3H, s, H-11), 1.80 (3H, s, H-10). ^{13}C NMR (175 MHz, $CDCl_3$): δ_C 171.2 (qC, C-1), 165.7 (qC, C-5), 156.7 (qC, C-7),149.1 (qC, C-3a), 112.8(qC, C-4), 102.8 (qC, C-7a), 98.6 (CH, C-6), 90.7 (qC, C-3), 74.6 (CH, C-8), 56.4 (CH_3, C-12), 62.4 (CH_2, C-9), 21.7 (CH_3, C-10), 11.5 (CH_3, C-11).

Clearanol D (**8**): white crystalline solid; LRESIMS $[M + H]^+$ *m/z* 269.1; $[M + Na]^+$ *m/z* 291.1; $[M - H]^-$ *m/z* 267.3; $[2M - H]^-$ *m/z* 535.2; $[2M + Na]^+$ *m/z* 559.3; $[\alpha]_D^{25} = +$ 3.8° (*c* 0.13, MeOH) ; ^{1}H NMR (700 MHz, CD_3OD): δ_H 6.46 (1H, s, H-6), 4.13(1H, dd, *J* = 3.5, 7.7 Hz , H-8), 3.85 (1H, dd, *J* = 10.2, 3.5 Hz , H-9a), 3.54 (1H, dd, *J* = 10.2, 7.7 Hz , H-9b), 3.86 (3H, s, H-12), 2.18 (3H, s, H-11), 1.67 (3H, s, H-10). ^{13}C NMR (175 MHz, CD_3OD): δ_C 171.8 (qC, C-1), 166.2 (qC, C-5), 157.7 (qC, C-7),152.9 (qC, C-3a), 113.2(qC, C-4), 105.2 (qC, C-7a), 99.4 (CH, C-6), 90.4 (qC, C-3), 75.5 (CH, C-8), 56.7 (CH_3, C-12), 63.7 (CH_2, C-9), 21.9 (CH_3, C-10), 11.2 (CH_3, C-11).

Dothideomynone A (**11**): pale yellow gun; LRESIMS $[M + H]^+$ *m/z* 255.1; $[M + Na]^+$ *m/z* 277.1; $[2M + Na]^+$ *m/z* 531.2; $[M - H]^-$ *m/z* 253.1, $[2M - H]^-$ *m/z* 507.2; $[\alpha]_D^{25} =$ +88.2° (*c* 0.13, MeOH) ; ^{1}H NMR (700 MHz, CD_3OD): δ_H 6.25 (1H, s, H-7), 3.79 (2H, overlapped , H-9), 2.06 (3H, s, H-11), 1.14 (3H, s, H-10). ^{13}C NMR (175 MHz, CD_3OD): δ_C 170.6 (qC, C-1), 164.7(qC, C-6), 163.4(qC, C-8),145.2 (qC, C-4a), 115.4(qC, C-5), 105.4 (qC, C-3), 101.1 (CH, C-7),99.9 (qC, C-8a), 65.4 (CH_2, C-9),

36.0 (CH, C-4),16.4 (CH_3, C-10), 10.0 (CH_3, C-11).

3,8-dihydroxy-3-hydroxymethyl-6-methoxy-4,5-dimethylisochromen-l-one (**12**): pale yellow oil; LRESIMS $[M + H]^+$ *m/z* 269.2; $[M + Na]^+$ *m/z* 291.1; $[M - H]^-$ *m/z* 267.1; $[\alpha]_D^{25} = + 58.8°$ (*c* 0.40, MeOH) ; ^1H NMR (700 MHz, CD_3OD): δ_H 6.25 (1H, s, H-7), 3.79 (2H, overlapped , H-9), 2.06 (3H, s, H-11), 1.14 (3H, s, H-10). ^{13}C NMR (175 MHz, CD_3OD): δ_C 170.5 (qC, C-1), 165.8(qC, C-6), 163.7(qC, C-8),144.2 (qC, C-4a), 116.3(qC, C-5), 105.2 (qC, C-3), 97.4 (CH, C-7),100.4 (qC, C-8a), 65.2 (CH_2, C-9), 56.2 (CH_3, C-12),36.4 (CH, C-4),16.4 (CH_3, C-10), 10.0 (CH_3, C-11).

(*R*)-4,8-dihydroxy-6-methoxy-4,5-dimethyl-3-methyleneisochromen-1-one (**13**): pale yellow oil; $[\alpha]_D^{25} = + 70.7°$ (*c* 0.12, MeOH) ; ^1H NMR (500 MHz, $CDCl_3$): δ_H 11.32(1H, br s, 8-OH), 6.40 (1H, s, H-7), 5.08 (1H, d, *J* = 2.0 Hz , H-9a), 4.94 (1H, d, *J* = 2.0 Hz , H-9b), 3.84 (3H, s, H-12), 2.36 (3H, s, H-11), 1.70 (3H, s, H-10). ^{13}C NMR (125 MHz, $CDCl_3$): δ_C 166.5 (qC, C-1), 165.8(qC, C-6), 163.2(qC, C-8), 160.7 (qC, C-3), 142.5(qC, C-4a), 116.5 (qC, C-5), 98.6 (CH, C-7), 98.2 (qC, C-8a), 95.5 (CH_2, C-9), 56.0 (CH_3, C-12), 72.1 (qC, C-4), 29.1 (CH_3, C-10), 12.0 (CH_3, C-11).

6, 8-dihydroxy-3,4-dimethylisocoumarin (**14**): white amorphous power; HRESIMS ($[M - H]^-$ *m/z* 205.0509, calcd for $C_{11}H_9O_4^-$, 205.0501). ^1H NMR (500 MHz, DMSO-*d6*): δ_H 11.32(1H, br s, 8-OH), 6.24 (1H, s, H-5), 6.29 (1H, s, H-7), 2.23 (3H, s, H-9), 1.99 (3H, s, H-10). ^{13}C NMR (125 MHz, DMSO-*d6*): δ_C 165.2 (qC, C-1), 168.0(qC, C-6), 163.1(qC, C-8), 149.4 (qC, C-3), 140.3(qC, C-4), 108.3 (qC, C-4), 101.3 (CH, C-7), 101.5 (CH, C-5), 96.7 (qC, C-8a), 16.8 (CH_3, C-9), 12.1 (CH_3, C-10).

Acremonone F (**15**): pale yellow gun; ^1H NMR (700 MHz, CD_3OD): δ_H 6.45(1H, s, H-7), 4.55 (2H, s, H-9), 4.74(2H, s, H-10), 2.50 (3H, s, H-11). ^{13}C NMR (175 MHz, CD_3OD): δ_C 167.8 (qC, C-1), 166.2(qC, C-6), 163.5(qC, C-8), 156.5 (qC, C-3), 138.5(qC, C-4a), 116.8 (qC, C-4), 114.1 (qC, C-5), 102.8 (CH, C-7),100.9 (qC, C-8a), 60.0 (CH_2, C-9), 57.6 (CH_2, C-10), 12.1 (CH_3, C-11).

Acremonone G (**16**): pale yellow oil; LRESIMS $[M - H]^-$ *m/z* 221.1;^1H NMR (700 MHz, CD_3OD): δ_H 6.47(1H, d, *J* = 2.0 Hz, H-6), 6.37(1H, d, *J* = 2.0 Hz, H-8), 4.47 (2H, s, H-11), 2.17 (3H, s, H-12). ^{13}C NMR (175 MHz, CD_3OD): δ_C 167.9 (qC, C-1), 167.3 (qC, C-7), 165.2 (qC, C-9), 152.1 (qC, C-3), 141.8(qC, C-5), 112.5(qC, C-4), 103.1(CH, C-8),102.7 (CH, C-6), 99.9(qC, C-10), 59.6 (CH_2, C-11), 12.0 (CH_3, C-12).

The strain's (*Leptosphaeria* sp. SCSIO 41005) ITS sequence of the rDNA
GGCCTTTCTTTATGAGAGAGTTGAGGTGGTTGAGTATCTCGCCCCTCAATTC
TCGCTGTATTTTACCCTTGTTTTTCTCA
TACTATTATTTCCTCGGCAGGCCAGCCTGCCGGGTGAAACAACTTCAAACC
TGTTTAATTTTCAATCAGCGTCTGAACAA
ATTAATAATTACAACTTTCAACAACGGATCTCTTGGTTCTGGCATCGATGAA

GAACGCAGCGAAATGCGATAAGTAGTGT
GAATTGCAGAATTCAGTGAATCATCGAATCTTTGAACGCACATTGCGCCCC
TTGGTATTCCATGGGGCATGCCTGTTCGA
GCGTCATTTGTACCTTCAAGCTCTGCTTGGTGTTGGGTGTTTGTCCTTGCTC
TAGTGGCGGGACTCGCCTTAAAGTAATT
GGCAGCCAGTGTTTTGGTTTTGAAGCGCAGCACAAGTCGCGATTCAAGTCT
ATACGCTAGTTTCCACAAGTCTTTTATCA
CTTTTGACCTCGGATCAGGTAGGGATACCCGCT
